# Antibody escape drives emergence of diverse spike haplotypes resembling variants of concern in persistent SARS-CoV-2 infections

**DOI:** 10.1016/j.xcrm.2026.102587

**Published:** 2026-02-02

**Authors:** Luke B. Snell, Suzanne Pickering, Adela Alcolea-Medina, Helena Winstone, Jeffrey Seow, Carl Graham, Lorcan O’Connell, Rahul Batra, Michael H. Malim, Katie J. Doores, Gaia Nebbia, Jonathan D. Edgeworth, Stuart J.D. Neil, Rui P. Galão

**Affiliations:** 1 Department of Infectious Diseases, School of Immunology and Microbial Sciences, King’s College London, London, UK; 2 Centre for Clinical Infection and Diagnostics Research, Department of Infectious Diseases, Guy’s & St Thomas’ NHS Foundation Trust, London, UK

**Keywords:** SARS-CoV-2, persistent infection, immune evasion, variants of concern, full-spike haplotypes

## Abstract

Evolution of SARS-CoV-2 in long-term persistent infections is hypothesized to be a major source of variants of concern (VOCs). However, linking intra-host variants into haplotypes that reflect viral subpopulations is limited by commonly used genomic sequencing techniques. We develop sequencing and analysis methods for identifying full-length spike haplotypes and analyze their diversification during persistent infections in individuals with inherited or acquired immunodeficiencies. This reveals accelerated evolutionary rates, with mutations frequently emerging at VOC-associated sites that confer escape from neutralizing antibodies, often undergoing strong positive selection. In a single infection lasting over 500 days from the first wave of the pandemic, we detail the evolution of spike as it acquires mechanisms to evade both autologous and heterologous neutralizing antibodies, redolent of Omicron variants. This evidence reinforces the argument for persistent infections being the source of immune-evasive variants, underscoring their impact on the evolutionary trajectory of SARS-CoV-2.

## Highlights

• Divergent intra-host spike haplotypes accumulate in persistent SARS-CoV-2 infections
• Positively selected mutations accrue at sites associated with variants of concern
• Spike evolves global neutralization escape over a 506-day wave 1 chronic infection
• These mutations are redolent of Omicron, 3 months before the emergence of BA.1

## Graphical abstract

**Figure:**
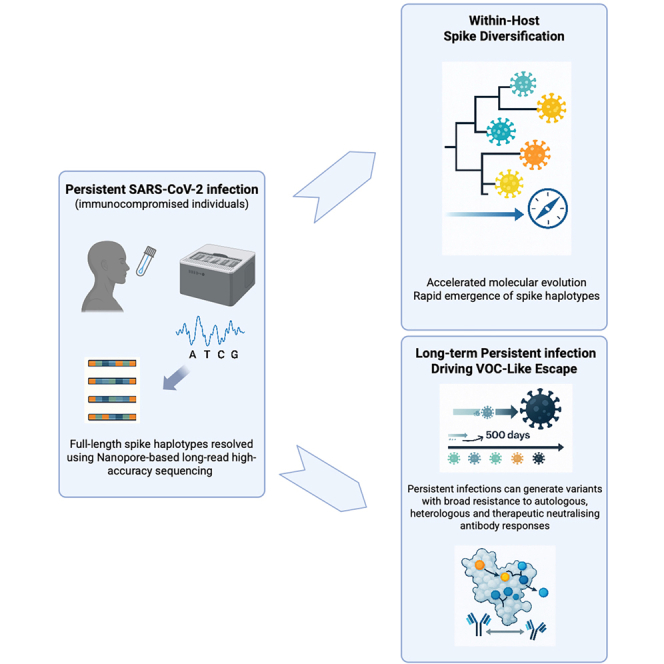

## Teaser

Snell et al. develop sequencing methodology to identify full-length spike haplotypes and show that persistent SARS-CoV-2 infections drive divergent haplotype emergence, accelerating viral evolution and promoting immune escape. Their work highlights how long-term infections in immunocompromised individuals can generate variants with enhanced resistance to neutralizing antibodies, shaping SARS-CoV-2’s evolutionary trajectory.

## Introduction

The emergence and evolution of SARS-CoV-2 variants of concern (VOCs) have resulted in successive waves of infection during the pandemic, frequently linked to highly divergent lineages, often described as “saltatory.”^1^ These lineages, responsible for rapid selective sweeps^2^ typified by the Alpha and Omicron waves,^3^ evolve mutations that enhance receptor binding,^4^^,^^5^ immune evasion,^6^^,^^7^^,^^8^^,^^9^^,^^10^ or replication efficiency.^11^^,^^12^ As a result, they exhibit increased transmissibility^13^^,^^14^ and altered disease severity,^15^^,^^16^ posing significant challenges to public health measures and vaccine effectiveness.^17^

Three main theories have been proposed to explain the sudden emergence of highly divergent VOCs. The first theory suggests that mutations gradually accumulate during cryptic chains of acute transmission. However, intermediate sequences leading to VOCs like Alpha or Omicron are rarely identified in global sequencing data.^18^ The second hypothesis involves cross-species “spillback” transmission events, where the virus adapts to a different host before re-entering humans, as observed in mink.^19^ The third and increasingly supported theory points to long-term persistent (LTP) infections, which occur primarily, but not exclusively, in immunocompromised individuals as a key factor in VOC evolution (reviewed by Markov et al., Sigal et al. and Machkovech et al.^20^^,^^21^^,^^22^). These prolonged infections may create a unique environment for viral evolution, enabling sustained replication, immune-driven selection pressures, and adaptations.^1^^,^^23^^,^^24^ LTP infections have been observed in individuals with either inherited or acquired immunodeficiencies, with the latter arising from disease-related immunosuppression or iatrogenic causes, such as medical therapy.^25^^,^^26^^,^^27^ Such cases often involve hematological malignancies or treatment with CD20-depleting therapies.^27^

Acute infections present strong purifying selection and narrow transmission bottlenecks that typically limit viral diversity,^28^^,^^29^ while chronic infections have been suggested to enable ongoing viral replication and the emergence of highly mutated immune escape variants.^30^ Additionally, therapeutic interventions such as monoclonal antibody and antiviral treatments may further drive viral adaptation within these immunocompromised hosts.^23^^,^^31^ This is mostly evident in the viral spike protein (S), which frequently exhibits multiple amino acid changes in domains responsible for host cell recognition and infection^25^^,^^32^ and is the primary site of diversity generation during LTP infections.^21^^,^^33^ Most of these mutations cluster in key antigenic regions of spike such as the N-terminal domain (NTD) and the receptor-binding domain (RBD) and are linked to increased resistance to immune defenses, particularly evasion from neutralizing antibodies.^34^

While persistent infections are recognized as potential reservoirs for VOC emergence, key questions remain about the mechanisms underlying intra-host viral evolution. The absence of transmission bottlenecks in immunocompromised individuals allows for greater intra-host diversity.^35^ However, not all persistent infections result in significant genetic divergence, as some cases show little to no accumulation of mutations.^36^ This highlights the need for advanced genomic approaches to better understand the natural history of persistent infections, the occurrence of adaptive mutations, the selective pressures driving these changes, and whether intra-host variation during particularly lengthy persistent infections contributes to the emergence of VOCs.

Previous genome sequencing strategies for studying the dynamic nature of SARS-CoV-2 intra-host variation during persistent infections have faced limitations.^37^^,^^38^ Short-read technologies fail to link mutations into haplotypes,^35^ while long-read sequencing approaches have historically suffered from high error rates, making them inaccurate for resolving intra-host variants.^37^^,^^38^ Finally, previous attempts to study the emergence of spike haplotypes in SARS-CoV-2 excluded genuine low-abundance variants that have not undergone clonal expansion, potentially missing intra-host evolutionary events.^39^ To overcome these challenges, we developed a long-read high-accuracy genome sequencing workflow, achieving 99.9% of base-calling accuracy, specifically optimized for haplotyping intra-host spike populations by capturing the entire spike gene in a single read. In this study, we applied this workflow to individuals with persistent SARS-CoV-2 infections to investigate intra-host spike variation and evolution as a potential driver of VOC emergence. We then examined the interaction between antibody response and intra-host viral evolution, providing insights into the evolutionary pathways that may contribute to the emergence of future SARS-CoV-2 variants.

## Results

### High-accuracy long-read sequencing workflow defines diversity of SARS-CoV-2 full-spike haplotypes in long-term persistent infections

**Figure 1:**
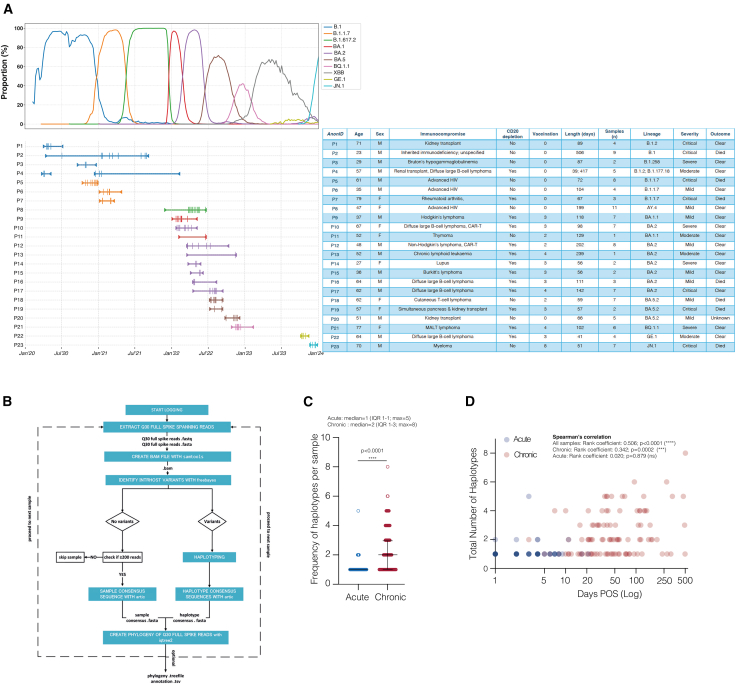
Newly developed high-accuracy long-read sequencing workflow shows diversity of SARS-CoV-2 spike haplotypes in chronic infections (A) Top panel: the prevalence of major SARS-CoV-2 lineages and their descendants in England, UK, from March 2020 to January 2024, based on GISAID data. Bottom panel: case timelines for patients with persistent SARS-CoV-2 infection, where symptom onset dates and final positive sample dates are marked with inverted triangles, and successfully sequenced samples are represented by vertical lines. Cases are color matched to the identity of the original infecting viral lineage. Table: overview of cases with persistent infection included in this study (see also Table S1). (B) Simplified diagram showing schematic of the HaploVar v1.0 bioinformatic workflow (see also Figure S1). (C) Number of haplotypes with a frequency greater than 5% in each sample from acutely infected individuals (blue, *n* = 69) and chronically infected patients (red, *n* = 115). Error bars represent mean with interquartile range across biological samples, and dots represent individual values for each sample. ∗∗∗∗*p* < 0.0001 as determined by Mann-Whitney test. (D) Scatterplot showing the number of days post onset of symptoms (POS; log) at which samples were taken against the number of spike haplotypes with a frequency greater than 5% in each sample. Data points are colored to represent acute (blue) and chronic (red) infections. Correlation between variables determined by Spearman’s; ∗∗∗∗*p* < 0.0001 and ∗∗∗*p* < 0.001.

To investigate the dynamic nature of SARS-CoV-2 intra-host variation during such prolonged infections, we identified 23 chronically infected patients (defined as individuals with continuous positive PCR tests for more than 30 days)^22^ with diverse underlying immunocompromised states such as immunosuppressive therapies, advanced HIV, autoimmune disorders, or cancer-related immunodeficiencies. These patients exhibited varied histories of vaccination and/or SARS-CoV-2 treatments (Figure 1A; Table S1). For this study, we collected 123 convenience longitudinal nasal and throat swab samples between April 2020 and January 2024 from these immunocompromised patients. Patients were persistently infected with various SARS-CoV-2 variants for durations ranging from 39 to 506 days (median = 93.5 days; IQR 58–139 days), with a median of 5 samples per patient successfully sequenced (IQR 2–7 samples; Figure 1A). 69 samples collected during the same period from individuals with acute SARS-CoV-2 infections were used as the control group (Table S2). The lineage distribution among the acute samples included 1 A, 16 B.1, 11 Alpha, 3 Delta, 3 BA.2, 1 BA.4, 14 BA.5, and 20 XBB lineages.

Our initial analyses employed conventional variant calling based on whole-genome sequencing of SARS-CoV-2 using both short-read ARTIC and long-read Midnight protocols. While this approach successfully produced high-quality consensus sequences for several samples, later time points revealed numerous positions with mixed base calls, precluding unambiguous amino acid assignment (data not shown). Strikingly, these ambiguous sites were non-random, occurring at residues predicted to be under antibody-mediated selective pressure (e.g., H655Y), indicating the coexistence of genetically distinct viral haplotypes within individual samples. Consequently, a haplotyping-based approach became necessary for further analysis. To resolve low-frequency intra-host single nucleotide variants (iSNVs), we developed a high-accuracy nanopore-based long-read workflow capable of spanning the entire SARS-CoV-2 spike coding region. This workflow produces Q30 reads using R10.4.1 flow cells (Oxford Nanopore Technologies), giving a 99.9% base-calling accuracy comparable to Illumina short-read technology, while simultaneously enabling haplotyping through its long-read capabilities.

The bioinformatic workflow “HaploVar 1.0” was designed to extract Q30 reads spanning the entirety of the spike gene sequence before the identification of intra-host variants (see STAR Methods) by haplotyping and using the ARTIC pipeline to generate consensus sequences of identified haplotypes. Maximum likelihood and molecular clock phylogenies were constructed from the haplotype consensus sequences (Figures 1B and S1).

To test the performance of the haplotyping workflow in identifying and quantifying intra-host variants, triplicate tests using artificial mixtures containing 10^4^ copies of viral RNA from Alpha and Beta lineages demonstrated that the workflow consistently detected single integer minority populations (Figure S2A). Furthermore, haplotypes above 5% were reproducibly identified with as few as 200 Q30 full-spike reads upon down-sampling of sequencing data from clinical specimens (Table S3), in accordance with others that also use a depth of 200 reads for calling intra-host variants.^35^ The lower limit of detection of the workflow for achieving the threshold of ≥200 Q30 reads required for successful haplotyping was 100 viral genome RNA copies (Figure S2B). Replicate sequencing of chronic clinical samples showed that haplotypes comprising more than 5% of the total were reproducibly detected (Figure S2C; Table S4). Therefore, a 5% minimum frequency threshold was applied for reporting of haplotypes throughout the study, which is similar to the 3% thresholds applied in Illumina sequencing.^36^

This high-accuracy workflow was then employed to explore how SARS-CoV-2 intra-host variation evolved during the course of acute and chronic infections in our panel of infected individuals (Figure 1A; Table S2). Of the 219 samples analyzed using the spike haplotype workflow, 69 of 96 acute samples and 115 of 123 chronic samples met quality control (QC) criteria. The Ct values for samples that passed and failed QC were both normally distributed (Shapiro-Wilk test: *p* = 0.227 and *p* = 0.907, respectively), with samples passing QC having significantly lower Ct values compared to those that failed (median = 18.0, SD = 4.1 vs. median = 23.0, SD = 4.4; *t* test: *p* < 0.0001; Figure S3A). For acute samples, the median number of haplotypes exceeding 5% frequency per sample was 1 (IQR: 1–1, max: 5), while chronic samples had a median of 2 haplotypes (IQR: 1–4, max: 8) among samples collected at least 30 days post-symptom onset, indicative of chronic infection (Figure 1C).

**Figure 2:**
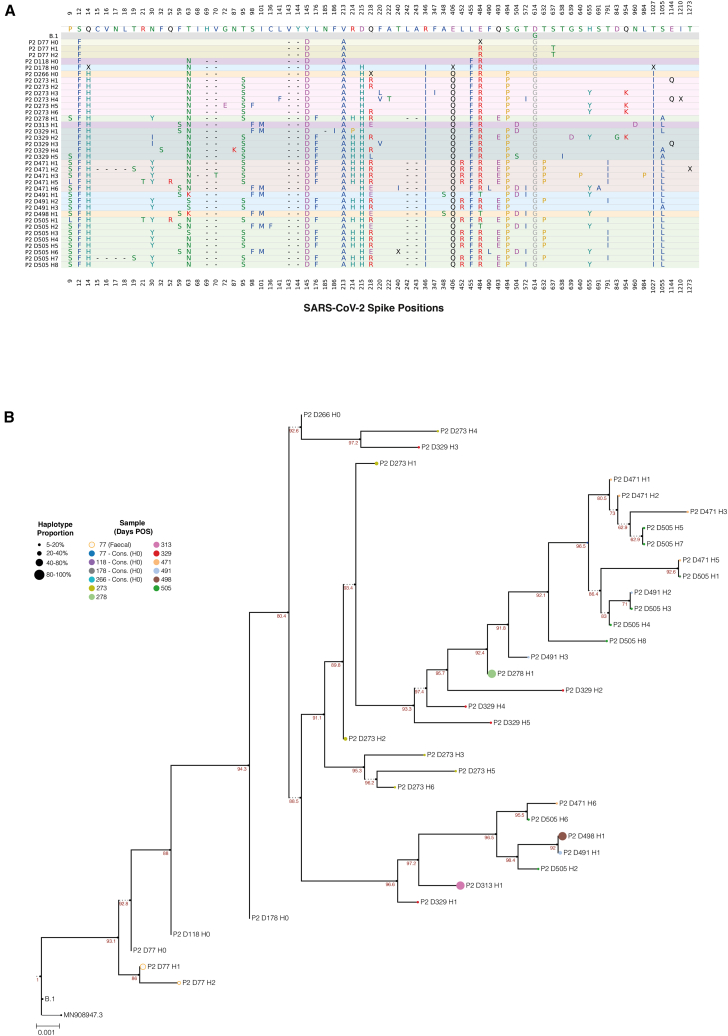
Example of intra-host evolution of full-length spikes during SARS-CoV-2 chronic infection (A) Non-synonymous mutations identified in haplotypes from longitudinal samples collected from patient 2. Note that this reflects the mutations detected at each sampling point, not necessarily the time of their emergence. Samples are displayed chronologically from the earliest to the latest, with each sample background uniquely shaded. Haplotype notation included the patient identifier (P), day of sampling post-onset of infection (D), and the haplotype rank in the sample (H). Haplotypes are ordered from highest to lowest proportion. For positions with non-synonymous mutations, the amino acid residue from the ancestral SARS-CoV-2 reference sequence (Genbank: MN908947.3) is listed first, followed by the corresponding residue(s) in the infecting variant. Non-synonymous mutations present in the infecting variant that remain unchanged during chronic infection are highlighted in gray. H0 notations on D77, D118, D178, and D266 reflect spike consensus obtained from whole-genome sequencing, as not enough material was available for haplotyping. Positions not called are represented by an “X”. (B) A divergence phylogeny of each haplotype representing at least 5% of total haplotypes identified at each longitudinally collected sample from a single persistent infection (P2), with the infecting variant designated as the outgroup. A maximum likelihood codon phylogeny version of this tree is shown in Figure S4B. H0 notations as in (A), where consensus sequences were added to the tree for completion. Tip labels are color-coded based on their corresponding sample time points, and their sizes are scaled to reflect the relative proportion of the haplotype within each sample. Branches are labeled with SH-like approximate likelihood ratio test results, with branches below 70% support collapsed.

Correlations were assessed between the number of haplotypes and the number of Q30 full-spike reads or Ct values to ensure that haplotype counts were not an artifact of the sequencing workflow. As expected, there was no significant correlation between the number of Q30 full-spike reads and haplotypes (Spearman’s rank coefficient (r) = −0.119; *p* = 0.107; Figure S3B), ruling out sequencing depth as a confounding factor. Similarly, no correlation was observed between the number of haplotypes and the Ct value of the sample (r = 0.114; *p* = 0.134; Figure S3C). Most importantly, the number of haplotypes in each sample showed a significant correlation with days post onset of symptoms (r = 0.506; *p* < 0.0001), which was naturally most prominent in samples from chronic infections (r = 0.342; *p* = 0.0002) when compared with acute samples (r = 0.020; *p* = 0.879; Figure 1D). As the final step in the workflow, spike haplotypes with a minimum 5% representation were used to construct divergence and molecular clock phylogenies. A representative analysis for patient 2 is illustrated in Figures 2A and 2B, while similar analyses for all patient samples are provided in Figures S4 and S5. In this case, we included spike sequences for D77, D118, D178, and D266, representing consensus sequences derived from whole-genome sequencing (WGS), as there was insufficient material for haplotyping. Notably, several positions were ambiguous in these WGS data. Subsequent haplotype analysis at later time points revealed that these positions harbored intra-host diversity (e.g., Q218R/E/L or E484R/T), explaining the inability to resolve them with WGS. In summary, we developed a high-accuracy long-read workflow able to resolve intra-host spike haplotypes present at low frequency, while demonstrating not only that diversity correlates with the length of infection but also that this is a highly dynamic process.

### Analysis of spike haplotypes from chronic infections reveals accelerated evolution rates, signatures of positive selection, and changes in residues associated with VOCs

**Figure 3:**
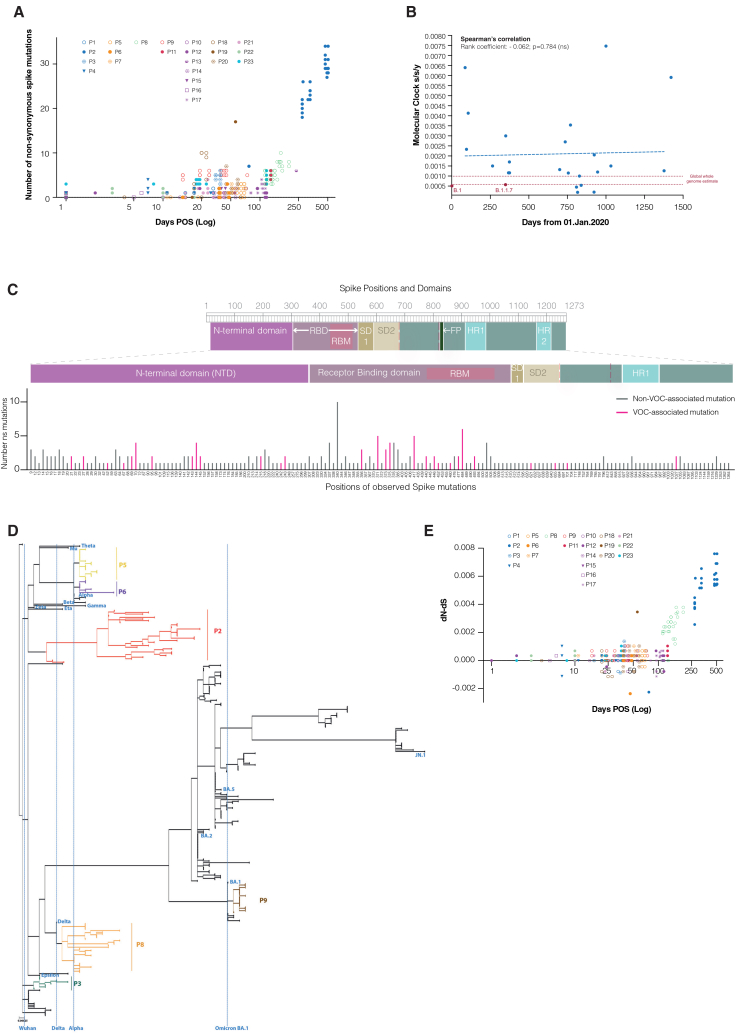
Evolution on spike haplotypes during long-term infections is characterized by changes in VOC-associated residues, accelerated evolutionary rates, and events of positive selection (A) Scatterplot depicting the number of non-synonymous spike mutations found in each spike haplotype against longitudinal sampling defined as days post-onset of symptoms (log). Individual patients have unique markers. (B) Scatterplot illustrating the correlation between the molecular clock (s/s/y) evolutionary rates estimated for the infecting variant spike of each patient (blue) or selected VOC spikes (red) against the date of infection, measured in days from January 01, 2020. Evolutionary rate intervals estimated for global whole genomes are represented by red dashed lines. Correlation determined by Spearman’s; ns > 0.05. See also Table S4. (C) Graphical representation of SARS-CoV-2 spike gene and corresponding protein domains (top panel). Number of non-synonymous mutations observed at each amino acid position in the spike protein from all patients (*n* = 23). Patients with multiple non-synonymous mutations at the same position are counted more than once. Positions without any mutations identified are absent. Bars are colored pink where the position corresponds to a lineage-defining mutation in VOC or a VOC-associated mutation (bottom panel). (D) Divergence phylogeny of spike haplotypes retrieved longitudinally from persistent infections and global VOC spikes. Selected patients are colored, and certain VOC lineages are labeled. The ancestral SARS-CoV-2 reference sequence (Genbank: MN908947.3) is designated as the outgroup. Branches with SH-like approximate likelihood ratio test results below 70% are collapsed. (E) Scatterplot depicting the difference of non-synonymous substitutions to synonymous substitutions (dN-dS) calculated for each haplotype retrieved from each persistent infection compared to the most abundant haplotype in the patient’s first successfully sequenced sample, plotted against the number of days post onset of symptoms (log) at which longitudinal sampling occurred. Individual patients have unique markers. See also Table S2.

Haplotypes present in samples were analyzed for mutations in the spike gene relative to the SARS-CoV-2 reference sequence and the infecting lineage (Figure 3A). The rate of spike-specific mutations in our cohort of persistently infected individuals exceeded that observed in the global population, with molecular clock analysis revealing cases such as P2, P4, P11, and P23 where evolutionary rates were considerably higher than those of contemporaneous VOCs (Table S5). There was no correlation between the rate of spike evolution and the timing of chronic infections during the pandemic (r = −0.062, *p* = 0.784) (Figure 3B). This suggests that the evolutionary patterns of chronic spikes have remained relatively consistent over time and are faster than those seen in acute infection.

Analysis of all spike haplotype populations identified in our panel of chronic patients revealed that non-synonymous mutations were primarily concentrated in highly antigenic regions such as the RBD and, to a lesser extent, the N-terminal domain (NTD; Figure 3C). Additionally, haplotypes were analyzed for the presence of lineage-defining VOC mutations or mutations at spike positions that are altered in VOCs (“VOC-associated mutations”). For this evaluation, lineages assigned a Greek letter by the World Health Organization were included for comparison, along with subsequent lineages of significant epidemiological importance (Tables 1 and S2). While a few cases showed no VOC lineage-defining mutations (P1, P13, P16, P18, P22, and P23), the majority acquired multiple VOC-defining mutations (e.g., P2: *n* = 7; P6, P7: *n* = 4; P8, P9: *n* = 5) and/or VOC-associated mutations (e.g., P2: *n* = 11; P20: *n* = 4; Table 1; Figure 3C; Table S2). Notably, a comparison with global SARS-CoV-2 spike diversity revealed extensive intra-host evolution in persistent infections, with some branch lengths exceeding the divergence observed between the Wuhan reference sequence and extant VOCs (Figure 3D). This is particularly evident with P2, where the patient was initially infected with the B.1 lineage and, during the length of a single chronic infection, viral spikes subsequently evolved a genomic distance nearing that of an Omicron BA.1 spike and exceeding that of a BA.2 spike (Figures 2A and 3D).

**Table 1:** List of VOC-defining and VOC-like mutations observed in spike haplotypes from patients with long-term persistent infections

Anonymized ID	Haplotypes (number)	dN/dS	VOC-like mutations	VOC-lineage-defining mutations
P1	7	0.57	–	–
P2	32	2.21	L8H, T19S, T95S, V213A, D215H, R346I, E484R, E484T, F490L, Q493E, Q954K	Q52R (Eta), 69/70- (Alpha), 143/144- (BA.1), 242/243- (Beta), L455F (XBB), L452R (Delta), H655Y (Omicron), T1027I (Gamma)
P3	8	0.72	A243V	69/70- (Alpha), T1027I (Gamma)
P4	9	1.15; 14.1	–	E484K (Beta)
P5	19	1.68	Y144V, W152L	–
P6	8	0.58	–	A67V (Eta), T95I (BA.1), D215G (Mu), E484K (Beta)
P7	5	23.5	–	S371F (BA.2), N440K (BA.2), E484Q (Kappa), A701V (Beta)
P8	30	0.84	L8K, S477I, D796H	K417T (Gamma), N440K (BA.2), S477N (BA.1), Q493E (KP.3), P621S (JN.1)
P9	18	27.2	S371I, N764E	S371L (BA.1), S365F (BA.1), K417N (Beta), K417T (Gamma), N460K (JN.1)
P10	10	1.39	K417I	K356T (JN.1)
P11	5	36.4	–	S371L (BA.1), S365F (BA.1), K417N (Beta)
P12	12	0.71	K356R	–
P13	1	N/A	–	–
P14	3	15.2	D796H	–
P15	2	N/A	Y248H	–
P16	6	0.43	–	–
P17	20	1.22	R346I, K356R, E484V	–
P18	14	0.43	–	–
P19	2	N/A	A570T	144- (BA.1)
P20	19	2.49	E156K, S371-, S373-, S375A, S375S, T376P/T	144- (BA.1)
P21	11	1.14	–	–
P22	9	37.1	–	–
P23	14	24.2	–	–

Abbreviations: N/A, not available.

Haplotypes were further analyzed for evidence of positive selection driving their evolution. Overall, 14 of 21 cases (67%) where spike dN/dS values could be calculated over the course of infection showed dN/dS > 1, consistent with positive selection (Table 1). Longitudinal analysis revealed that 65% (156/240) of haplotypes had a positive dN–dS value, indicating positive selection, while 24% had no mutations (Figure 3E; Table S2). Phylogenetic analysis identified 36 spike positions under episodic diversifying selection across 11 patients (Table 2). Sixteen of these positions were associated with VOCs, suggesting frequent emergence and positive selection of VOC-like mutations in persistent infections. Notably, 1/36 position (P330S) was previously linked to chronic infections,^40^ while 5/36 positions (P337, E340, A344, G446, and G476) were associated with resistance to monoclonal antibodies.

**Table 2:** Results of phylogenetic testing to identify amino acid positions under episodic diversifying selection overall, at specific time points, and sites evolving differentially over time

Anonymized ID	Episodic diversifying selection overall	Episodic diversifying selection at specific time point	Sites evolving differentially between time points
P1	nil	nil	nil
P2	9, 30, 63, 95, 218, 220, 240, 452, 484, 632, 637, 1,055	637, 960 (d77), 218 (d273)	9, 63, 218, 1,055
P3	1,253	nil	243, 1,027
P4	nil	nil	Nil
P5	nil	nil	Nil
P6	nil	nil	64, 95
P7	371	nil	Nil
P8	337, 446S/446V, 476, 477, 489	337, 446S, 446V (d142)	18, 337
P9	nil	nil	337, 340, 460
P10	nil	385 (d43)	385
P11	936, 987	936, 987 (d129)	936
P12	nil	385 (d1)	340, 385
P13	fail	fail	fail
P14	796	nil	nil
P15	515	515 (d0)	515
P16	nil	nil	515
P17	nil	344 (d95), 337 (d104)	51
P18	200	115 (d21)	200
P19	nil	nil	nil
P20	375	156, 323 (d59)	156, 505, 1,020
P21	330	nil	330
P22	nil	nil	nil
P23	376	376	nil

Positions are colored if VOC associated (purple), reported as conferring resistance to monoclonal antibodies (blue), or previously described to be associated with persistent infections (green).

**Figure 4:**
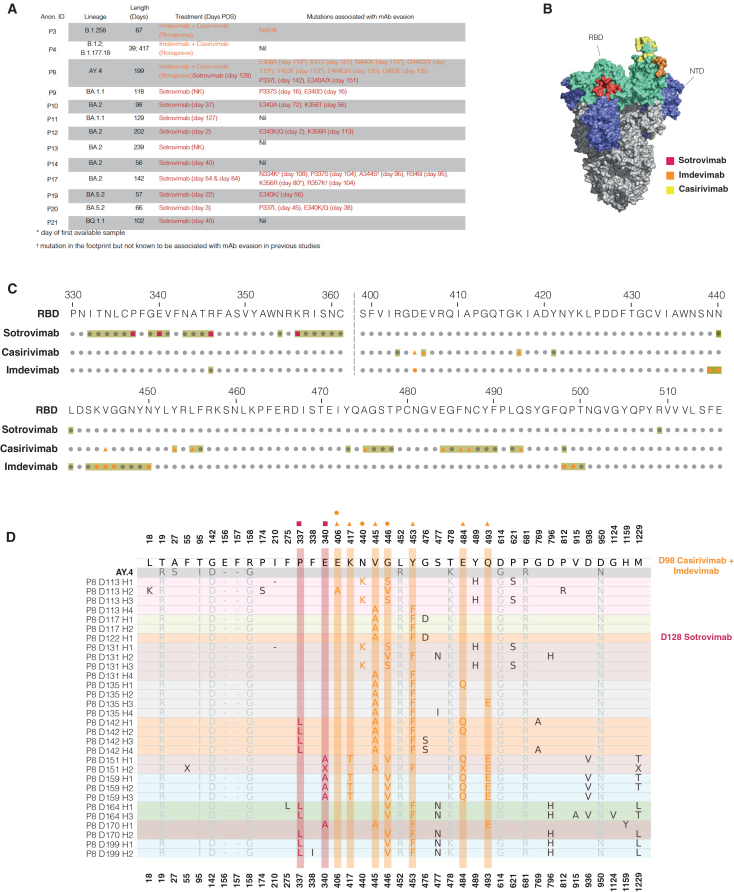
Mutations linked to monoclonal antibody evasion can rapidly emerge following treatment in chronically infected patients (A) Details of clinical cases involving long-term persistently infected patients treated with sotrovimab (red) and/or combinations of imdevimab and casirivimab (orange), along with identified mutations linked to evasion from these monoclonal antibodies (mAb). Mutations present on the day of the first available sample are indicated with (∗), while those observed within the mAb-binding footprint but not yet associated with mAb evasion are marked with (†). (B) Structure of full spike (S) of SARS-CoV-2 showing binding footprints for sotrovimab (red) and casirivimab (REGN10933, yellow) together with imdevimab (REGN10987, orange). RBD and NTD domains of spike are labeled in cyan and blue, respectively. mAb footprints for sotrovimab were determined using PDB: 6WPS, and the Regeneron mAbs with PDB: 6XDG. (C) The full RBD sequence of ancestral SARS-CoV-2 (Genbank: MN908947.3), with mAb binding footprints highlighted in light green. Mutations detected in spike haplotypes from patients treated with mAbs are marked in red for sotrovimab and in orange for the casirivimab and imdevimab combination. (D) Non-synonymous mutations identified in haplotypes from longitudinal samples collected from patient 8. Samples are displayed chronologically from the earliest to the latest, with each sample background uniquely shaded. Dates of first treatment with mAbs are indicated on the right as days post-onset of symptoms. Haplotype notation included the patient identifier (P), day of sampling post-onset of infection (D), and the haplotype rank in the sample (H). Haplotypes are ordered from highest to lowest proportion. For positions with non-synonymous mutations, the amino acid residue from the Wuhan reference sequence (Genbank: MN908947.3) is listed first, followed by the corresponding residue(s) in the infecting variant. Non-synonymous mutations on mAb footprints are highlighted in red for sotrovimab and in orange for the casirivimab and imdevimab combination. Non-synonymous mutations present in the infecting variant that remain unchanged during chronic infection are highlighted in gray. Positions not called are represented by an “X”.

Based on this observation, we analyzed spike haplotypes from patients in our cohort who received monoclonal antibody treatment during their chronic infection to examine how mutations evolved in targeted regions (Figures 4A–4C). Among the ten patients treated with sotrovimab, seven acquired mutations associated with evasion at one or more spike positions—P337, E340, R346, and/or K356 (Figure 4A). P17 exhibited three further mutations on the defined sotrovimab footprint that were not previously associated with resistance to this monoclonal antibody—N334K, A344S, and R357K (Figures 4A–4C). Additionally, two of three patients treated with casirivimab and imdevimab developed known resistance mutations within the corresponding binding regions (Figures 4A–4C). P8 exemplified how resistance mutations can evolve following mAb treatment during chronic infections. This patient received casirivimab and imdevimab on day 98 post-onset of symptoms, and by day 113, several resistance mutations (E406A, N440K, V445A, G446S/V, and Y453F) had already emerged. However, as this is the earliest available sample for this patient, it cannot be excluded that at least some of these mutations were already present. Additional resistance mutations, such as K417T, E484Q, and Q493E, appeared later in infection (Figure 4D). Furthermore, on day 128, the same patient was treated with sotrovimab, and known resistance mutations P337L and E340A were detected soon after on days 142 and 151, respectively. The emergence of multiple resistance mutations, none of which were detected in untreated patients, strongly suggests that monoclonal antibody therapy can quickly drive the selection of escape mutations in persistent infections.

Collectively, these findings provide compelling evidence that elevated evolutionary rates occur during chronic infections, in part driven by events of positive selection, while supporting the hypothesis that long-term persistent infections play a key role in the evolution of variants of concern.

### Immune pressure leads to an evolving universal evasion from wave 1 neutralizing responses

To directly assess intra-host immune pressures that may contribute to the evolution of spike, we performed detailed studies on longitudinal samples obtained from one individual with an exceptionally long disease course (P2 in Figure 1A; Table S1). This individual became symptomatic and tested positive for SARS-CoV-2 in April 2020 and was admitted to hospital in June 2020 (day 77) with a persistent infection with lineage B.1. They were treated with remdesivir but declined compassionate treatment with antibody-based therapies. The patient passed away in September 2021, on day 506 of the infection, with multiple co-morbidities and overlapping acute illnesses (Figure 1A).

**Figure 5:**
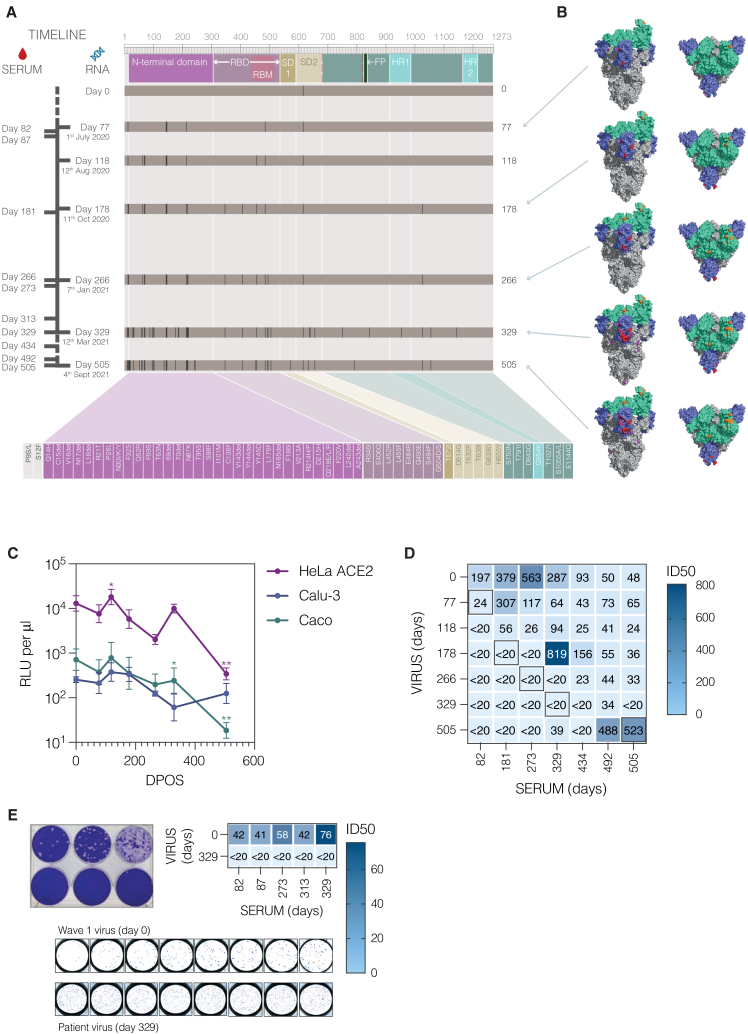
Longitudinal characterization of intra-host SARS-CoV-2 spike evolution and humoral immune evasion during a long-term chronic infection in one individual (A) Schematic representation of SARS-CoV-2 spike sequence evolution in one individual over the course of a 506-day infection. A timeline of serum samples and nasal swabs from this individual is shown on the left as days post-infection, with a schematic of the spike protein, including all major domains, at the top. The accumulation of individual amino acid mutations is shown over time and mapped to spike protein domains. The identity of each of these mutations is listed at the bottom, colored according to the spike domain in which they occur. (B) Trimeric spike structures showing the location of accumulating amino acid mutations. RBD is shown in green, and NTD in blue. RBD mutations are highlighted in orange, NTD in red, and visible mutations in other parts of spike are shown in purple. (C) Assessment of the infectivity of longitudinal spike proteins over the 506-day infection. Lentiviral vectors were pseudotyped with sequential SARS-CoV-2 spike proteins from days 0, 77, 118, 178, 266, 329 (P2 D329 H2 in Figure 2A), and 505 (P2 D505 H1 in Figure 2A) post infection. Pseudoviruses were titrated on HeLa ACE2, lung-derived (Calu-3), and colon-derived (Caco) cell lines, and titers were calculated as RLU per μL of pseudovirus. Means and standard deviations are derived from 3 independent experiments. Titers for each time point were compared with day 0 (D614G) spike on each cell line by two-way ANOVA with Dunnett’s multiple comparisons (HeLa ACE2 day 118 ∗*p* = 0.023 and day 505 ∗∗*p* = 0.0018; Caco day 329 ∗*p* = 0.029 and day 505 ∗∗*p* = 0.0064; all other comparisons were non-significant). (D) Heatmap of autologous longitudinal humoral immune response over the course of a chronic SARS-CoV-2 infection in one individual. Neutralizing responses were assessed using lentiviral vectors pseudotyped with sequential SARS-CoV-2 spike proteins from days 0, 77, 118, 178, 266, 329 (P2 D329 H2 in Figures 2A and S6A), and 505 (P2 D505 H1 in Figures 2A and S6A) post infection (shown in (A)) and longitudinal autologous serum samples from days 82, 181, 273, 329, 434, 492, and 505. Each square shows reciprocal mean neutralizing titers (ID50) for a given pseudovirus-serum pair, derived from two independent experiments, with color intensity proportional to neutralization potency. Boxed numbers represent mean ID50 values for contemporaneous virus-serum pairs (or where not available, the closest possible match). (E) Infectious virus was isolated from a day 329 nasal swab, with plaque morphology shown in the left panel. Neutralization of the day 0 ancestral virus was compared with the day 329 virus using sequential autologous serum samples from days 82, 87, 273, 313, and 329 post-infection. Each square shows reciprocal mean neutralizing titers (ID50) for a given infectious virus-serum pair, derived from two independent experiments, with color intensity proportional to neutralization potency. An example of results seen in the mini plaque reduction assay is shown in the bottom panel.

Spike genes were cloned from nasal swabs obtained at regular time points throughout infection (77, 118, 178, 266, 329, and 505 days post-infection; Figure 5A). Samples were not available from this individual prior to day 77; therefore, for the purposes of comparison, the day 0 (infecting) virus was assumed to be an ancestral D614G sequence, which represented the majority known spike sequence circulating at the time in the UK. Haplotype analysis from this individual demonstrated increasing diversification of viral sequences over time, resulting in 5 and 8 spike haplotypes (amounting to 42% and 89% of the total quasispecies) identified at days 329 and 505, respectively (Figures 2A and 2B). For these time points the 5 most abundant haplotypes were cloned. In parallel, full-length infectious virus was successfully cultured from a day 329 nasal swab.

A total of 55 amino acid mutations were detected in the spike protein throughout the course of infection in this one individual (Figures 5A and S6A; Table S6). The majority of these mutations occurred in antigenic regions of spike (Figure 5B), with 32 in the NTD and 8 in the RBD. Several of the mutations overlapped with known VOC lineage-defining mutations or VOC-associated mutations (Table 1), with the clustering reminiscent of that seen for Omicron BA.1 (Figure S4B). The impact of accumulating mutations on infectivity and ACE2 usage was assessed by comparing titers of lentiviral vectors pseudotyped with spikes from sequential time points on three ACE2-expressing cell lines: HeLa ACE2, lung-derived Calu-3, and colon-derived Caco (Figures 5C and S7A; Table S6). With the exception of the very last available sample (day 505), all spikes derived from patient 2 showed robust ability to infect the target cells, on a par with that seen for the D614G spike (Figures 5C and S7A).

To investigate the impact of accumulating mutations on escape from humoral immunity, neutralization assays were performed with lentiviral vectors pseudotyped with spikes from sequential time points and longitudinal autologous serum samples, as close to contemporaneous as available (Figures 5A, 5D, S7B, and S8A; Table S6). Overall, titers were low compared with typical titers seen in immunocompetent cohorts (e.g., Dupont et al.^41^), yet all serum samples tested were able to neutralize the D614G ancestral variant (Figure 5D; day 0 virus). A shifting window of immune escape was seen, with serum samples that preceded the spike isolation time point having no neutralizing effect, contemporaneous serum-spike pairs showing weak or no neutralization, and serum samples later than the spike time point increasing in neutralizing potency, peaking at approximately 100–150 days after the time of spike isolation (Figure 5D). The day 505 spike was the exception to this rule, with contemporaneous sera neutralizing the most common spike haplotype from this time point. Results with autologous sera were confirmed using full-length virus isolated from a day 329 nasal swab (Figures 5E and S8B; Table S6). While all serum samples tested (days 82, 87, 273, 313, and 329) were able to neutralize the day 0 virus (ancestral B.1), none of them neutralised the day 329 virus. Additionally, we tested serum samples from all other available eligible serum samples from the cohort (i.e., from individuals that did not receive anti-SARS-CoV-2 monoclonal antibody therapy or antiretroviral therapy for HIV infection). Neutralizing activity against D614G was detected in an additional two individuals, patients 1 and 4 (Figure S9; Table S6), with ID50 values of a similar magnitude to those seen for patient 2.

Together, these results clearly illustrate a stepwise evolution of spike driven by the humoral immune response, resulting in spikes that are completely refractory to neutralization by earlier autologous antibodies while maintaining the ability to efficiently infect ACE2-expressing lung- and intestinal-derived target cells.

**Figure 6:**
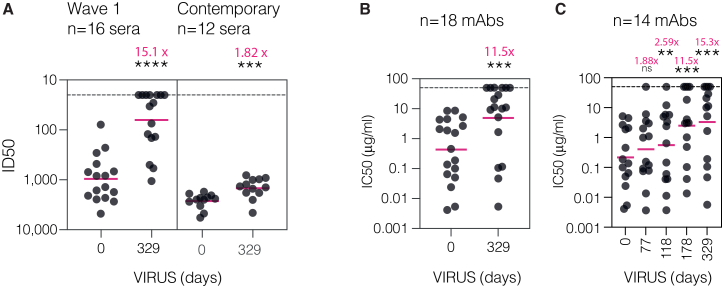
Further characterization of humoral immune evasion during long-term chronic infections (A) Neutralization of spike from assumed infecting virus (day 0; ancestral D614G) and day 329 spike-pseudotyped virus by early acute sera (10–31 DPOS) from individuals infected in wave 1 of the pandemic (left panel) and by recently collected contemporary sera (right panel). Each point represents the mean ID50 value for a given serum sample, derived from two independent experiments, with the pink line indicating the geometric mean titer (GMT) for *n* = 16 wave 1 sera and *n* = 12 contemporary sera. Neutralizing titers of the two pseudoviruses were compared using a two-tailed paired Wilcoxon signed rank test (∗∗∗∗*p* < 0.0001 and ∗∗∗*p* = 0.0005). Numbers in pink indicate the fold change in GMT between the groups. (B) Neutralization of day 0 and day 329 spikes was assessed using *n* = 18 monoclonal antibodies. Each point is the mean IC50 value for a given mAb, derived from two independent experiments. The pink line shows the overall GMT for all mAbs against each spike, with numbers in pink indicating the fold change in GMT. Significance was determined by two-tailed Wilcoxon signed rank test (∗∗∗*p* = 0.0001). (C) 14 monoclonal antibodies were used for more detailed stepwise escape from wave 1 monoclonal antibodies using spikes from days 0, 77, 118, 178, and 329. Pink lines indicate GMT for *n* = 14 mAbs against each spike, with numbers in pink showing the fold change in GMT between each spike and the ancestral (day 0) spike. Significance was determined by two-tailed Wilcoxon signed rank test (day 77 *p* = 0.54; day 118 ∗∗*p* = 0.0085; day 178 ∗∗∗*p* = 0.0004; day 329 ∗∗∗*p* = 0.0006).

To assess whether the neutralization escape by later spikes was specific only to the autologous neutralizing response or represented a generalized escape from typical wave 1 responses, 16 heterologous serum samples from acute wave 1 infection were compared for neutralization potency against the ancestral D614G (day 0) and day 329 spikes (Figures 6A and S10). Neutralization titers against the day 329 spike were significantly lower than the D614G ancestral (day 0) spike, with an overall 15.1-fold difference between the geometric mean titers (GMTs). However, this was not representative of being generally refractory to neutralization, as recent serum samples obtained in the post-omicron era^42^ showed robust neutralizing activity against both the D614G (day 0) and day 329 spikes (Figures 6A and S10).

Thus, a spike protein isolated late in a long-term infection demonstrates significant immune escape from both autologous and heterologous humoral immune responses, analogous to that seen for later SARS-CoV-2 saltatory variants such as BA.1.^43^^,^^44^ Yet, still similar to BA.1, it remained susceptible to neutralization from serum samples obtained later in the pandemic from individuals exposed to omicron variants.^42^

To probe the escape from wave 1 neutralization in more detail, a panel of monoclonal antibodies isolated from individuals infected during wave 1,^45^ or naive individuals vaccinated with ancestral spike-based vaccine,^46^ were tested against lentiviral vectors bearing spikes from P2. 18 mAbs were tested in total, comprising a range of potencies and binding specificities. Similar to results seen with wave 1 serum samples, neutralization of the day 329 spike was significantly weaker than the D614G ancestral (day 0) spike, with an 11.5-fold difference in GMT (Figure 6B). This was further dissected to assess stepwise escape using spikes from days 77, 118, 178, and 329 (Figure 5C). The accumulation of individual RBD mutations over time (Figures 5A and S6A) led to an incremental neutralization escape, with each time point accounting for a shift in GMT (Figure 6C), with a bigger change occurring between day 118 and 178 due to two RBD mutations (R346I and E406Q). Separating the results into individual mAb binding groups allowed a more precise mapping of the effects of these mutations on neutralization (Figure S11A; Table S6). In particular, we observed consistent evasion of group 4 mAbs over time, which were the most common binding group isolated from vaccinated individuals.^46^ Commercial mAbs, used therapeutically prior to the post-Omicron era, provided additional opportunities to map details of immune escape, given that structures are available for these mAbs in complex with the SARS-CoV-2 spike. While the effects of imdevimab and sotrovimab fluctuated slightly over time, the overall trend was for the P2 spikes to remain susceptible to neutralization by these mAbs (Figures S11B and 6A), in contrast to the effects seen with the group 4 mAbs in Figure S11A. Interestingly, casivirimab was rendered completely ineffective by day 178. The escape can be attributed to the acquisition of an E484R mutation from day 77 onwards, an L455F mutation from day 118 onwards, and then an E406Q mutation at day 178, all of which are located in the casivirimab footprint.^47^ The commercial mAbs were also tested with multiple haplotypes from two time points (days 329 and 505), confirming that haplotypes from the same time points gave broadly similar results for the different mAbs.

In summary, we have dissected the evolution of spike in a single, exceptionally long-term SARS-CoV-2 infection and provided clear evidence that intra-host humoral immune pressure drives the characteristic accumulation of spike mutations seen in novel VOCs. We have mapped out a pathway whereby highly mutated viruses can arise in one individual, beginning with infection by an ancestral virus in the first wave of the pandemic and culminating in a variant that is broadly similar in its mutation profile and immune evasion properties to Omicron 3 months prior to the latter’s emergence in southern Africa.

## Discussion

This work describes the development of a long-read sequencing workflow integrated with the bioinformatic tool, HaploVar 1.0, designed here for detecting and characterizing diverse spike haplotypes that emerge during long-term persistent infections. Haplotypes exhibited significant divergence and signs of positive selection, with many sharing key spike mutations found in VOCs. We then used these data to conduct phenotypic characterization of these spike haplotypes, in one of the longest persistent SARS-CoV-2 infections on record. Through these studies, we mapped out a pathway whereby a highly mutated variant can arise in a single individual, driven by escape from the autologous antibody response but resulting in a generalized resistance to first wave neutralizing responses. Compared to previous studies, our approach provides a more comprehensive view of intra-host viral diversity and population dynamics over time, while also allowing for the assessment of positive selection and uniquely enabling the linkage between genotype and phenotype.

As shown in this study, persistent SARS-CoV-2 infections can lead to viral sequences that exhibit an unusually high number of mutations compared to the original infecting strain, indicating accelerated evolution relative to acute infections. These sequences show an overrepresentation of non-synonymous substitutions, sometimes with several amino acid changes occurring at the same site, as well as small deletions and insertions, suggesting rapid adaptation within chronically infected individuals rather than neutral diversification.^22^^,^^25^^,^^30^^,^^48^ During acute infection transmission chains, where each infection is brief, viral populations undergo severe transmission bottlenecks.^28^^,^^49^ However, intra-host transmission between cells typically lacks such constraints, allowing continuous viral replication with reduced selection pressures. Over time, multiple mutations can accumulate within individual viruses and a greater diversity of variants persists. This increases the chances of creating favourable constellations of changes, which may then give rise to a new VOC. The rate of SARS-CoV-2 evolution in chronically infected individuals varies (reviewed in^21^), but, here, we demonstrate that cases can display mutation rates comparable to or exceeding those leading to saltatory emergence of VOCs, while other cases of persistent infection exhibit a slower evolutionary pace similar to contemporaneous circulating lineages. These differing findings may reflect the inherent challenges in interpreting consensus sequences from persistent infections, as such sequences might not capture the full complexity of the viral population. Depending on the prevalence of co-existing variants, consensus sequences may resemble the original strain more closely, potentially underestimating the full range of viral diversity. The sequencing workflow developed in this study addresses these limitations, offering a more precise depiction of the dynamic intra-host evolution of the spike protein during persistent infections. Importantly, unlike other methods such as high-throughput, single-copy sequencing (HT-SGS),^39^ this approach captures linked variants directly, without requiring statistical phasing or assembly, thus preserving variant phasing within single reads and enabling the resolution of complex mutations, including insertions, deletions, and structural changes.

Our study substantiates the theory that long-term persistent infections can drive the emergence of VOCs and underscores how studying these individuals may provide valuable insights to predict future developments in the SARS-CoV-2 pandemic that may also be of relevance to the selection of antigenic drift variants in other endemic respiratory viruses. We specifically note that, in certain cases of prolonged infection, the evolutionary trajectories of the identified spike haplotypes exhibit long branch lengths, akin to those seen in contemporaneously emerging VOCs such as Beta, Delta, and Omicron. For example, spike proteins from P2 contained a total of 55 possible mutations, including seven VOC-defining mutations and 11 VOC-like mutations. Phylogenetic analysis further indicates that many of these positions are under diversifying selection.

SARS-CoV-2 is well-documented to evolve known neutralizing antibody escape mutations during persistent infections,^35^^,^^40^^,^^48^^,^^50^ but, here, our unique access to longitudinal paired viral sequences and serum samples over the course of an exceptionally long-persistent infection has allowed us to document the stepwise creation of a highly evolved Omicron-like variant. We demonstrate a shifting window of evasion from weak neutralizing responses in an immunocompromised individual, paralleling classic studies of virus-host dynamics seen for other chronic infections like HIV.^51^^,^^52^ This resulted in a virus with universally reduced sensitivity to first wave antibody responses from immunocompetent individuals, in the form of heterologous sera, monoclonal antibodies isolated from vaccinees and infected individuals, and a commercial therapeutic monoclonal antibody. Interestingly, this is achieved with relatively few mutations in the RBD, including some that have been more recently associated with recent Omicron sub-variants. For example, L455F, known for forming the so-called FLiP mutations with F456L, which have recently appeared in the JN.1 lineage and are recognized for their role in immune evasion and antibody escape,^53^ as well as mutations at R346 and Q493E. However, in contrast to VOCs and particularly the Omicron sub-variants, in which the majority of mutations arise in the RBD, we observed a larger number of deletions and substitutions in the NTD of the P2 spikes. The potential effects of these on the conformation of the spike and exposure of key neutralizing epitopes remain to be characterized. Additionally, we observed spike haplotypes in P2 carrying the H655Y mutation, a hallmark of the Gamma and Omicron VOCs, which has been linked to enhanced viral replication, changes in spike protein cleavage and altered cell entry pathways,^54^ facilitating transmission.^55^ Remarkably, spike haplotypes with mutations now recognized as critical in the pandemic had already evolved by the time the final sample from P2’s long-term persistent infection was collected in September 2021, 3 months before the emergence of the Omicron BA.1 variant. This highlights the potential of studies like this to identify and characterize key mutations that might evolve in future VOCs.

Selective pressure in long-term persistent infection can also be externally driven, for example, through the administration of convalescent plasma^50^ or monoclonal antibodies,^35^^,^^56^ as demonstrated in this study for the latter. In line with earlier findings,^57^ we observed swift viral adaptations following treatments with monoclonal antibodies. This was marked by the frequent emergence of well-documented escape-related mutations, including P337L and E340—linked to resistance against sotrovimab—as well as E484Q and Q493E, associated with escape from imdevimab and casirivimab. Together, these observations underscore how quickly such adaptations can arise, raising concerns about the long-term utility of monoclonal antibody therapy in chronic infections.

To definitively demonstrate the role of persistent infections in the emergence of VOCs, it is crucial to show that VOC-like variants arising in such contexts are capable of onward transmission. While some SARS-CoV-2 variants (e.g., Alpha) achieved global spread primarily through enhanced transmissibility rather than immune evasion, others (e.g., Omicron) combined immune escape with increased fitness. Thus, the transmission advantage of SARS-CoV-2 VOCs has been linked not only to adaptive immune escape but also to other immune evasion mechanisms, increased infectivity, altered cell entry processes, and changes in spike protein cleavage and structure. Although our findings show that mutations do occur on P2 spike haplotypes at sites among those inferred to have positive effects on SARS-CoV-2 VOC transmission^58^—such as L455F, H655Y, and modifications in R346 and Q954—our selection analyses have also identified previously unreported loci, offering potential insights into adaptation during host-pathogen interactions. However, several of the most prominent mutations did not evolve. These include N460K and Q498R, associated with enhanced fusogenicity and spike processing, increased ACE2 binding affinity, and transmissibility,^59^^,^^60^^,^^61^ and P681H/R, suggested to enhance spike cleavage and shown to confer a level of resistance to IFN-beta.^10^^,^^62^ Together, these might reflect that while highly divergent lineages may be transmitted,^63^^,^^64^ they often fail to sustain transmission at the population level.^65^ The relative rarity of these population saltation events may be linked to distinct selective pressures driving evolution, as mutations selected during persistent infections have been proposed to favor intra-host rather than inter-host transmission.^66^ This trade-off occurring in within-host persistent lineages most often results in evolutionary dead ends, as mutations that confer strong immune evasion may compromise viral fitness or reduce transmissibility.^6^ In contrast, variants that persist globally must maintain high replication efficiency and transmission potential.

During the last stages of revision of this manuscript, two other studies performing genomic analysis of long-term persistent infections were published.^67^^,^^68^ While these studies did not assess haplotype diversity or phenotypic spike changes as done here, they similarly support the notion that viral adaptation to the immune system is the main evolutionary driver. However, although different types or degrees of immunocompromise can possibly lead to divergent evolutionary patterns, there is no clear evidence that host factors such as source of altered immune status, age, sex, vaccination status, or virus lineage influence evolutionary rates.^67^

Future studies should explore the role of specific mutation constellations in shaping viral phenotypes, including immune escape and fitness. To date, limited research has examined these dynamics, but the long-read sequencing workflow presented here offers a powerful tool for studying the phenotypic effects of linked mutations. These insights could enhance our understanding of how mutations interact to influence viral evolution and transmission.

### Limitations of the study

This study has several limitations. First, although individual cases of prolonged SARS-CoV-2 infection allowed detailed characterization of intrahost viral evolution, we did not demonstrate increased transmissibility of emergent variants, which is necessary to support the hypothesis that intrahost variants evolved during persistent infection are the source of VOCs. A shared limitation of our study and others, together with their retrospective nature, is that only nasopharyngeal swabs were analyzed, which may have led to an underestimation of viral population diversity by overlooking subpopulations present in the deeper airways or the gut. In addition, our sequencing approach cannot reliably detect minority variants below a few percent frequency, meaning that low-abundance subpopulations could have been missed.

Second, the number of individuals with chronic SARS-CoV-2 infection from whom sequential serum samples and nasopharyngeal swabs were available was low, meaning autologous neutralization responses for most cases could not be evaluated. This reflects both logistical constraints on blood collection and the exclusion of patients receiving anti-SARS-CoV-2 monoclonal antibody therapy from studies assessing neutralization. Moreover, because our neutralization assays rely on SARS-CoV-2-pseudotyped lentiviral particles, they cannot be performed using serum from individuals receiving antiretroviral therapy for HIV, restricting analysis for an additional subset of patients with available samples.

Finally, future work investigating factors such as HLA typing and T cell responses could provide deeper insights into the mechanisms driving viral evolution, while characterizing non-spike mutations arising during chronic infections may shed light on innate immune adaptations that support viral persistence.

## Resource availability

### Lead contact

Further information and requests for resources and reagents should be directed to and will be fulfilled by the lead contact, Rui P. Galao (rui_pedro.galao@kcl.ac.uk).

### Materials availability

There are restrictions to the availability of some of the clinical samples used in this study due to their scarcity or the lack of remaining material. This includes nasopharyngeal swabs and sera. All further unique/stable reagents generated in this study will be made available on request after completion of a materials transfer agreement (MTA). All requests for resources and reagents should be directed to the lead contact.

### Data and code availability

• Sequencing data have been deposited at the NCBI Sequencing Read Archive as (BioProject: PRJNA1247580) and are publicly available at the date of publication. Source data related to neutralization assays are available in this paper’s supplemental information. Sequences used to synthesize patient spikes have been deposited at Genbank: PV551127–PV551139.
• All original code for HaploVar v1.0 is publicly available at Zenodo: https://doi.org/10.5281/zenodo.17692842 as of the date of publication.
• Any additional information required to reanalyze the data reported in this work paper is available from the lead contact upon request.

## Acknowledgments

We are extremely grateful to all patients and staff at St Thomas’ Hospital who participated in this study. We thank all colleagues in the G2P consortia for the insightful discussions, as well as Prof. Emma Thompson, Dr. Ana Da Silva Filipe, and teams for review of initial genomic findings. We would like to thank Prof. Sergei Pond for his advice on performing phylogenetic selection analysis and Prof. Florence Débarre for her valuable feedback on the manuscript. This research was funded by the UK Medical Research Council Discovery Awards to the G2P/G2P2 consortia (MC/PC/15068 and MR/Y004205 to M.H.M., K.J.D., S.J.D.N., and R.P.G.), the Wellcome Trust to the G2P-global consortium (226141/Z/22/Z to M.H.M., K.J.D., and S.J.D.N.), and the Huo Family Foundation. This study was further supported by the UK Department of Health via an NIHR Comprehensive Biomedical Research Centre award to 10.13039/501100004941Guy's and St Thomas' NHS Foundation Trust in partnership with King’s College London and 10.13039/100010872King's College Hospital NHS Foundation Trust. S.J.D.N. was supported by a Wellcome Trust Senior Fellowship (WT098049AIA). M.H.M. was supported by 10.13039/100010269Wellcome Trust awards (106223/Z/14/Z and 222433/Z/21/Z). L.B.S. was supported by UK Medical Research Council Fellowship (MR/W025140/1). The funding sources of this study had no influence in study design, data collection and analysis, data interpretation, or the preparation of the report or in the decision to submit this manuscript for publication.

## Author contributions

Planning and conceptualization, L.B.S., S.P., K.J.D., G.N., J.E., S.J.D.N., and R.P.G.; investigation, methodology, and data analysis, L.B.S., S.P., A.A.-M., H.W., J.S., C.G., L.O.’C., and R.P.G.; funding acquisition, L.B.S., R.B., M.H.M., K.J.D., G.N., J.E., S.J.D.N., and R.P.G.; software, L.B.S.; resources, K.J.D. and J.E.; writing the manuscript, L.B.S., S.P., S.J.D.N., and R.P.G.; review and editing the manuscript, L.B.S., S.P., A.A.-M., H.W., J.S., C.G., L.O.’C., R.B., M.H.M., K.J.D., G.N., J.D.E., S.J.D.N., and R.P.G.

## Declaration of interests

J.D.E. is employed part-time as the VP of Medical Affairs by Oxford Nanopore Technologies.

## STAR★Methods

### Key resources table

**Table:** 

REAGENT or RESOURCE	SOURCE	IDENTIFIER
**Antibodies**		

SARS-CoV-2 spike-specific mAbs	Graham et al.^69^; Seow et al.^46^	N/A
Sotrovimab	Guy’s & St. Thomas’ NHS Foundation Trust	N/A
Imdevimab	Guy’s & St. Thomas’ NHS Foundation Trust	N/A
Casirivimab	Guy’s & St. Thomas’ NHS Foundation Trust	N/A
Wave 1 representative sera	Dupont et al.^41^	N/A
CR3009 (murinised N specific mAb)	Expressed in-house	N/A
Goat anti-mouse IgG (Fc-specific)-peroxidase antibody	Sigma	Cat#A2554; RRID:AB_258008

**Bacterial and virus strains**		

SARS-CoV-2 B.1 reference strain	Public Health England	England 02/2020/407073
Patient 2, day 329 isolate	This paper	N/A
SARS-CoV-2 Alpha (B.1.1.7)	NIBSC	Cat#101019
SARS-CoV-2 Beta (B.1.351)	NIBSC	Cat#101022

**Biological samples**		

Nucleic Acid Extracts from nasal and throat swabs	Guy’s & St. Thomas’ NHS Foundation Trust	REC Approval: 20/SC/0310
Autologous sera from chronical infected individuals	Guy’s & St. Thomas’ NHS Foundation Trust	REC Approval: 20/SC/0310

**Critical commercial assays**		

QIAsymphony DSP Virus/Pathogen Mini Kit	QIAGEN	Cat#937036
Qubit™ dsDNA Quantification, High Sensitivity	Thermo Fischer Scientific	Cat#Q32851
SuperScript™ IV One-Step RT-PCR System	Invitrogen	Cat#12594025
Qubit™ 3.0 Fluorometer	Thermo Fischer Scientific	Cat#Q33216
Qubit™ Assay Tubes	Thermo Fischer Scientific	Cat #Q32856
Native Barcoding Kit 23 V14	Oxford Nanopore Technologies	Cat#SQK-NBD114.24
R10.4.1 flow cells	Oxford Nanopore Technologies	Cat #FLO-MIN114
Gridion Mk1	Oxford Nanopore Technologies	Cat #GRD-MK1
NEBNext® Ultra™ II End Repair/dA-Tailing Module	New England Biolabs	Cat #E7546S
NEBNext® Quick Ligation Module	New England Biolabs	Cat#E6056S
SARS-CoV-2 N gene standard	TIB MOLBIOL	Cat#30-7454-71
Luna® SARS-CoV-2 RT-qPCR Multiplex Assay Kit	New England Biolabs	Cat# E3019S
Steady-Glo® Luciferase Assay System	Promega	Cat#E2520
TrueBlue peroxidase substrate	SeraCare	Cat#50-78-02

**Deposited data**		

Code for HaploVar v1.0	This paper	Zenodo: https://doi.org/10.5281/zenodo.17692842
Sequencing data	This paper	BioProject: PRJNA1247580
Sequences of synthesized spikes	This paper	GenBank: PV551127-PV551139

**Experimental models: Cell lines**		

HEK 293T/T17	ATCC	ATCC CRL 11268TM
Vero-E6	ATCC	ATCC CRL 1586TM
Vero-E6-TMPRSS2	Winstone et al.^10^	N/A
HeLa-ACE2	James Voss (Scripps Research, San Diego)	N/A

**Oligonucleotides**		

SARS-CoV-2 spike forward primer: 5′aggggtactgctgttatgtcttt3′	Matsubara et al.^70^	N/A
SARS-CoV-2 spike reverse primer 5′aggcttgtatcggtatcgttgc3′	Matsubara et al.^70^	N/A

**Recombinant DNA**		

p8.91 – (HIV-1 Gag-Pol)	Zufferey et al.^71^	N/A
pCSXW (HIV-firefly luciferase). Constructed by replacing GFP in pHR’SIN-SEW (PMID: 11975847) with firefly luciferase.	Luis Apolonia, (King’s College London)	N/A
Plasmid expressing SARS-Cov-2 B.1 spike	Nigel Temperton, (Medway School of Pharmacy, Anson)	N/A
Plasmids expressing SARS-CoV-2 P2 spikes	GenScript. This paper	GenBank: PV551127-PV551139

**Software and algorithms**		

MinKnow v23.04.5	Oxford Nanopore Technologies	https://nanoporetech.com/software/devices/gridion
Dorado v0.3.1	Oxford Nanopore Technologies	https://github.com/nanoporetech/dorado
Samtools v1.10	Danecek et al.^72^	https://github.com/samtools/samtools/releases/
HaploVar v1.0	This paper	http://github.com/GSTT-CIDR
Nanofilt v2.8.0	De Coster et al.^73^	https://github.com/wdecoster/nanofilt
bedtools v.2.30.0	Quinlan et al.^74^	https://github.com/arq5x/bedtools2
seqtk v1.3	NA	https://github.com/lh3/seqtk
freebayes v1.3.7	NA	https://github.com/freebayes/freebayes
awk	Aho et al.^75^	https://github.com/onetrueawk/awk
gofasta v1.2.1	Jackson^76^	https://github.com/virus-evolution/gofasta
ARTIC bioinformatic pipeline	NA	https://github.com/artic-network/fieldbioinformatics
IQ-TREE v.2.3.0	Minh et al.^77^	https://www.iqtree.org
ETE 3 v3.1.3	Huerta-Cepas et al.^78^	https://etetoolkit.org/
TimeTree v0.11.3	Kumar et al.^79^	https://github.com/neherlab/treetime
Mega11	Tamura et al.^80^	https://www.megasoftware.net
HYPHY v2.5.62	Kosakovsky Pond et al.^81^	https://hyphy.org
Mixed Effects Model of Evolution (MEME)	Murrell et al.^82^	https://stevenweaver.github.io/hyphy-site/methods/selection-methods/#meme
Contrast-FEL	Kosakovsky Pond et al.^83^	https://hyphy.org/methods/other/contrast-fel/
TempEST v1.5.3	Rambaut et al.^84^	https://tree.bio.ed.ac.uk/software/tempest/
SciPy v1.9.3	Virtanen et al.^85^	https://pypi.org/project/scipy/
GraphPad Prism v10	Dotmatics	https://www.graphpad.com

### Experimental model and study participant details

Patients were identified at a referral center for COVID-19 cases.^27^ Cases of acute (69), and persistent infection (23 patients, 15 male and 8 female) (Figure 1; Table S2) were identified in line with previous definitions^22^ - acute cases involved individuals without immunocompromise who were asymptomatic at the time of their initial positive test, while chronic infections were defined by PCR positivity lasting at least 30 days. Residual longitudinal nasal and throat swab samples and autologous serums from SARS-CoV-2-infected individuals at St. Thomas’ Hospital were retrieved at the point of being discarded from April 2020 to January 2024, and processed under existing ethics, which did not require patient consent and allowed linked data to be retrieved from routinely collected notes (20/SC/0310, South Central - Hampshire B Research Ethics Committee). Severity of illness was categorised as per World Health Organisation definitions.^86^ Given the relatively restricted number of identified patients with persistent infections, as well as their diverse immunosuppressive background, we are not able to report on the association of sex, gender or both on the results of the study.

HEK293T/17 (ATCC CRL 11268), Vero-E6 (ATCC CRL 1586), Calu-3 (ATCC HTB-55) and Caco (ATCC HTB-37) cells were obtained from the American Type Culture Collection. Vero-E6 cells were modified to stably express TMPRSS2 via lentiviral vector transduction.^10^ HeLa-ACE2 cells were generously provided by James E. Voss. All cell lines were cultured in DMEM supplemented with GlutaMAX (Gibco, UK) and 10% FCS, maintained at 37°C with 5% CO_2_. All cell lines were regularly tested for mycoplasma contamination.

### Method details

#### Amplification of the spike gene sequence and high quality long-read sequencing

Nucleic acid extraction utilised the QIAGEN QIAsymphony SP system in combination with the QIAsymphony DSP Virus/Pathogen Mini Kit (Qiagen), following the off-board lysis protocol. To create amplicons spanning the full length of the spike gene sequence, nucleic acid extracts were subjected to reverse transcription polymerase chain reaction (RT-PCR) using the SuperScript IV One-Step RT-PCR System (Invitrogen) with primers flanking the spike region (Forward: AGGGGTACTGCTGTTATGTCTTT; Reverse: AGGCTTGTATCGGTATCGTTGC)^70^ under the following incubation conditions: 10 min at 55.0°C; 2 min at 98.0°C; 40 cycles of 10s at 98.0°C, 10s at 64.6°C, 2 min 20 s at 72.0°C; followed by final extension of 5 min at 72.0°C. PCR products were quantified using the high sensitivity Qubit dsDNA Quantification kit (Thermo Fisher) on the Qubit 3.0 Fluorometer (Thermo Fisher). 400 fmol of PCR products were prepared for sequencing using the Native Barcoding Kit 24 V14 (SQK-NBD114.24, Oxford Nanopore Technologies, UK) according to manufacturer’s conditions with the following modifications. For the end preparation reaction 7μL of Ultra II End Repair/dA-Tailing Module Reaction Buffer and 3μL of Ultra II I End-prep Enzyme Mix (NEB) were used and incubated for 15 min at 20°C and 15 min at 65°C. The final library was eluted in a 30 μL volume. 20 fmol of the final library was sequenced using R10.4.1 flow cells on the GridION Mk1 (Oxford Nanopore Technologies) for 24 h.

#### Bioinformatic analysis for the identification of spike haplotypes

Demultiplexing of sequencing data was performed using onboard MinKnow v23.04.5 (Oxford Nanopore Technologies). Duplex basecalling was performed using dorado v0.3.1 (https://github.com/nanoporetech/dorado) with further processing into fastq using samtools v1.10 (https://github.com/samtools/).

To identify unique spike haplotypes, fastq files were then processed using HaploVar v1.0 (https://doi.org/10.5281/zenodo.17692842, Figure S1). Briefly, reads with a minimum quality of Q30 (99.9% basecalling accuracy) were first filtered with Nanofilt v2.8.0 (https://github.com/wdecoster/nanofilt) and reads are identified that fully span the spike region using samtools and bedtools v.2.30.0 (https://github.com/arq5x/bedtools2), taking reads crossing both the start (21,563) and end position (25,384) of spike against Wuhan reference genome (Genbank: MN908947.3). The identified Q30 full spike spanning reads are then subsampled from the total duplex read pool using seqtk v1.3 (https://github.com/lh3/seqtk). Bam files were created from this high quality subsampled full-length spike reads using samtools.

Variants (SNPs, deletions and insertions) were identified in these bam files using freebayes v1.3.7 (https://github.com/freebayes/freebayes), identifying variants at a minimum of 1% frequency in the read pool and with a minimum depth of 200 based on previous reports for identifying intra-host variants.^35^ The output from freebayes was queried using awk (https://github.com/onetrueawk/awk) to identify positions of variants not fixed in the read pool to identify intra-host variants. Next, the reads were further processed to identify haplotypes, specifically how the identified intra-host variants exist in linkage with each other in each read. To achieve this, the Q30 full-length spike reads were converted to fasta format using seqtk (https://github.com/lh3/seqtk) before being mapped against the Wuhan reference genome using gofasta v1.2.1 (https://github.com/virus-evolution/gofasta). Reads with ambiguous mapping were discarded. For each full-length spike read, the nucleotide at each position of intra-host variation was extracted, discarding the rest of the read where the positions were invariant, giving a haplotype for each read. Unique haplotypes within the read pool were then identified, the frequency of the occurrence of each haplotype in the read pool was counted, and reads were separated into discrete read pools according to haplotype using seqtk. Consensus sequences for each haplotype were then created using a modified version of the ARTIC bioinformatic pipeline (https://github.com/artic-network/fieldbioinformatics) reflecting the custom primer scheme and a minimum coverage depth of 10x. Haplotypes with frameshifts were discarded.

#### Testing ability of the spike haplotyping workflow to determine minority variants

To allow determination of viral copy number a standard curve was established using a quantified N-gene from an ancestral SARS-CoV-2 genome (TIB Molbiol 30-7454-71) and applying an RT-qPCR method (NEB Luna SARS-CoV-2 RT-qPCR Multiplex Assay Kit). Absolute viral copy number was determined for B.1.1.7 (National Institute for Biological Standards and Control, #101019) and B.1.351 lineage (#101022) before artificial mixing 10^4^ genome copies with minority variant at 0%, 0.5%, 1%, 2%, 5%, 10%, 20%, before testing with the spike haplotyping workflow.

#### Phylogenetic and selection analysis

Maximum likelihood phylogenies were derived using IQ-TREE v2.3.0^77^ fitted using a codon-aware model with codon-aware ancestral state reconstruction and branch testing utilising Shimodaira–Hasegawa-like approximate likelihood-ratio test with 1000 replicates. Trees were further processed in ete3 v3.1.3 (https://etetoolkit.org/) for display. Molecular-clock phylogenies were constructed in TimeTree v0.11.3 (https://github.com/neherlab/treetime).

Rates of synonymous and non-synonymous mutation were calculated using the Nei-Gojobori method (Jukes Cantor model) in Mega11^80^ assuming the standard genetic code. As most spike haplotypes lacked synonymous mutations, the synonymous substitution rate for these haplotypes was calculated as 0 substitutions/site/year, rendering dN/dS undefined; thus, dN-dS is reported instead. Variation in rates were assumed to be gamma distributed (shape parameter = 1.0) across the spike gene region and gaps were treated as pairwise deletions. Ambiguous positions were not included in comparisons. For each patient, the reference haplotype used for comparison in these calculations was the most abundant haplotype in the first successfully sequenced sample. Note these data represent mutations detected at each sampling point, not necessarily the time of their emergence.

Tests were performed to identify which sites were under selective pressure using HYPHY v2.5.62.^81^ Overall dN/dS rates for each persistent infection were computed using the Muse-Gaut 1994 codon model with universal genetic code, imposing a global branch model in which a single nonsynonymous-to-synonymous rate ratio (ω) is shared across all branches. Rate heterogeneity was fitted with a gamma distribution utilising 4 distinct rate classes. Branch lengths were re-estimated simultaneously with model parameters by maximum likelihood. Mixed Effects Model of Evolution (MEME)^82^ was used to test sites under episodic selection pressure both overall and at specific timepoints. Fixed effects likelihood (Contrast-FEL)^83^ was used to determine which sites have significantly different rates of change between timepoints. Phylogenic resampling was set at 1000 replicates. Molecular clocks utilized TempEST v1.5.3 (https://tree.bio.ed.ac.uk/software/tempest/).

When evaluating mutations in haplotypes, the lineages considered for comparison were any assigned a Greek letter by the World Health Organisation (i.e., Alpha, Beta, Gamma, Delta, Episilon, Kappa, Eta, Iota, Lambda, Epsilon, Theta, Zeta, Mu, Omicron) and predominant lineages that came after Greek letters were assigned (BA.1-5, JN.1 and KP.3).

#### Pseudovirus production

Sub-confluent HEK293T/17 cells were transfected in 10 cm dishes with 2 μg of the HIV-1 8.91 Gag-Pol plasmid, 3 μg of the CSXW (HIV-firefly luciferase) plasmid, and 2 μg of the SARS-CoV-2 spike plasmid using 35 μg of PEI-Max (1 mg/mL, Polysciences). Cells were incubated at 37°C, and the medium was replaced 6–12 h post-transfection. Pseudoviruses were harvested 72 h after transfection and filtered through 0.45 μm filters. Low-titre preparations were concentrated via ultracentrifugation through a sucrose cushion. To achieve this, pseudoviruses were initially treated with 10 U/mL of recombinant DNase I (Merck) in the presence of 10 μM MgCl_2_ for 2 h at 37°C. The DNase-treated preparations were then layered over a 20% sucrose cushion in PBS and ultracentrifuged at 28,000 rpm for 1 h and 30 min. After centrifugation, the supernatant was removed, and the pellets were resuspended in serum-free DMEM GlutaMAX.

All spike plasmids used in this study were codon-optimized and included full-length cytoplasmic tails. The SARS-CoV-2 B.1 spike plasmid was generously provided by Prof. Nigel Temperton. Longitudinal spikes representing the most common haplotypes from patient 2 were synthesized by GenScript (GenBank PV551127-PV551139).

To determine pseudovirus titers, supernatant was 5- or 10-fold serially diluted in DMEM GlutaMAX (Gibco, UK) then 50 μL was added per well of a 96-well plate. HeLa-ACE2 cells were prepared at a concentration of 5 × 10^5^ cells/mL, and 50 μL (2.5 × 10^4^ cells/well) was added to each well. Plates were incubated for 72 h at 37°C before measuring luciferase activity as relative light units (RLU) using the Steady-Glo Luciferase Assay System (Promega, UK) with a VICTOR X Multilabel Reader (PerkinElmer). To compare infectivity of pseudoviruses in HeLa-ACE2, Calu-3 and Caco cell lines, the above procedure was carried out with the exception that 4 × 10^4^ Calu-3 cells or 4 × 10^4^ Caco cells were added per well. RLU was plotted against μl of pseudovirus and titers (RLU/μL)determined by regression analysis.

#### Viruses

The UK SARS-CoV-2 Wave 1 (lineage B.1) reference strain, England 02 (England 02/2020/407073), was obtained from Public Health England. Full-length viruses were isolated from patient nasal and throat swab samples by diluting 200 μL of the sample in 1.5 mL of DMEM GlutaMAX containing 2% FCS, filtering through 0.45 μm filters, and infecting Vero-E6-TMPRSS2 cells. Supernatants were collected upon the appearance of visible cytopathic effects (CPE). For virus propagation, 100 μL of the isolated virus was added to confluent Vero-E6-TMPRSS2 cells cultured in 75 cm^2^ flasks with DMEM GlutaMAX supplemented with 2% FCS. Cells were monitored daily, and cultures were harvested upon visible CPE. The harvested cultures were filtered through 0.45 μm filters, aliquoted, and stored at −80°C until use.

#### Plaque assays

To determine viral titers, plaque assays were conducted in 6-well plates. Virus samples were serially diluted 10-fold, and 500 μL of each dilution was added per well to confluent Vero-E6-TMPRSS2 cells, followed by incubation at 37°C for 1 h. After incubation, 500 μL of a pre-warmed overlay (0.1% agarose in DMEM GlutaMAX supplemented with 2% FCS) was added to each well, and the plates were incubated at 37°C for 72 h. Cells were fixed with 4% formaldehyde at room temperature for 30 min, then stained with 0.05% crystal violet in ethanol for 5 min. Wells were washed with PBS, air-dried, and plaques were counted. Viral titers were calculated by averaging the results from three independent assays.

#### Neutralisation assays

Neutralisation assays were performed using longitudinal serum samples from patient 2, as well as wave 1 representative sera,^41^ sera collected from healthy donors in November/December 2024,^42^ and monoclonal antibodies (mAbs) representative of different competing binding groups,^46^^,^^69^ and the commercial mAbs sotrovimab, imdevimab and casirivimab.

Neutralization assays with pseudovirus were conducted using HeLa-ACE2 cells.^46^ mAbs or serum (heat-inactivated at 56°C for 30 min before initial use) were serially diluted in DMEM GlutaMAX (Gibco, UK) and incubated with pseudovirus at 37°C for 1 h. HeLa-ACE2 cells were prepared at a concentration of 5 × 10^5^ cells/mL, and 50 μL (2.5 × 10^4^ cells/well) was added to each well. Plates were incubated for 72 h at 37°C before measuring luciferase activity using the Steady-Glo Luciferase Assay System (Promega, UK) with a VICTOR X Multilabel Reader (PerkinElmer). For neutralisation assays with full-length SARS-CoV-2 infectious virus, mini plaque reduction neutralisation tests (PRNT) were performed.^46^ Vero-E6-TMPRSS2 cells were seeded the day before infection at a density of 30,000 cells per well of a 96-well plate in DMEM supplemented with 2% FCS. Sera were serially diluted in DMEM GlutaMAX (Gibco, UK) and incubated with virus (optimised to achieve 80–200 plaques per well) at 37°C for 1 h (50μl of virus was added to 50ul of diluted sera) before adding to cells. This was incubated for a further 1 h at 37°C before the addition of pre-warmed carboxymethylcellulose overlay (Sigma-Aldrich, C4888) to a final concentration of 0.5%. Plates were incubated 37°C for 16–20 h before removing supernatant and fixing with 4% formaldehyde in PBS (30 min at room temperature). Fixed cells were then washed in PBS, permeabilised in 0.2% Triton X-100 for 15 min at room temperature, then blocked with 3% milk in PBS for 15 min at room temperature before addition of murinised anti-nucleocapsid antibody CR3009 (2ug/ml) for 45 min at room temperature. Plates were washed twice with PBS and incubated for a further 30 min at room temperature with secondary antibody goat anti-mouse IgG (Fc-specific)-peroxidase (Sigma A2554; 2 μg/ml). Plates were washed twice with PBS before addition of TrueBlue peroxidase substrate (50μl per well; SeraCare 50-78-02). Plates were incubated for 20–60 min until clear, dark blue plaques were visible, before removal of substrate and air drying. Plaques were identified and counted using an AID EliSpot Reader with EliSpot 8.0 software.

### Quantification and statistical analysis

Statistical analyses were performed using both GraphPad Prism v10 (GraphPad Software Inc.), and SciPy v1.9.3 in python v3.1.1 (https://pypi.org/project/scipy/). Statistical power of interquartile ranges comparisons was determined by Mann-Whitney test. Strength and direction of the relationship between two variables were determined by Spearman’s rank correlation. Titers of different pseudotypes were compared for each time point and with control group on distinct cell lines by two-way ANOVA with Dunnett’s multiple comparisons. Neutralising titers were compared using two-tailed paired Wilcoxon signed rank tests. Measures are expressed as mean ± SEM and significance levels were set at *p* < 0.05 compared with control conditions. Finally, normality of passed and failed samples was tested using Shapiro-Wilk, in which *p* > 0.05 confirms the hypothesis of normality.

## Supplemental information

Document S1. Figures S1–S11 and Tables S1 and S3–S5

Table S2. Supplemental Database (spreadsheet), related to Table 1, Figure 1, and Figure 3 (1) Line list of all samples processed. (2) List of VOC mutations. (3) List of mutations found in spike haplotypes for each patient. (4) Synonymous and non-synonymous rates in each haplotype.

Table S6. Source data related to neutralization assays in Figures 5–6 and Figures S7 and S9 (spreadsheet)

Document S2. Article plus supplemental information
