# Supplementary material for: Antibody escape drives emergence of diverse spike haplotypes resembling variants of concern in persistent SARS-CoV-2 infections
Source: Cell Rep Med. 2026 Feb 2;7(2):102587. doi: 10.1016/j.xcrm.2026.102587 (PMC12923952; doi:10.1016/j.xcrm.2026.102587)
Supplement: Document S1. Figures S1–S11 and Tables S1 and S3–S5 [file mmc1.pdf]

Cell Reports Medicine, Volume *7*

Supplemental information

Antibody escape drives emergence of diverse spike haplotypes resembling variants of concern in persistent SARS-CoV-2 infections

Luke B. Snell, Suzanne Pickering, Adela Alcolea-Medina, Helena Winstone, Jeffrey Seow, Carl Graham, Lorcan O'Connell, Rahul Batra, Michael H. Malim, Katie J. Doores, Gaia Nebbia, Jonathan D. Edgeworth, Stuart J.D. Neil, and Rui P. Galão

Figure S1

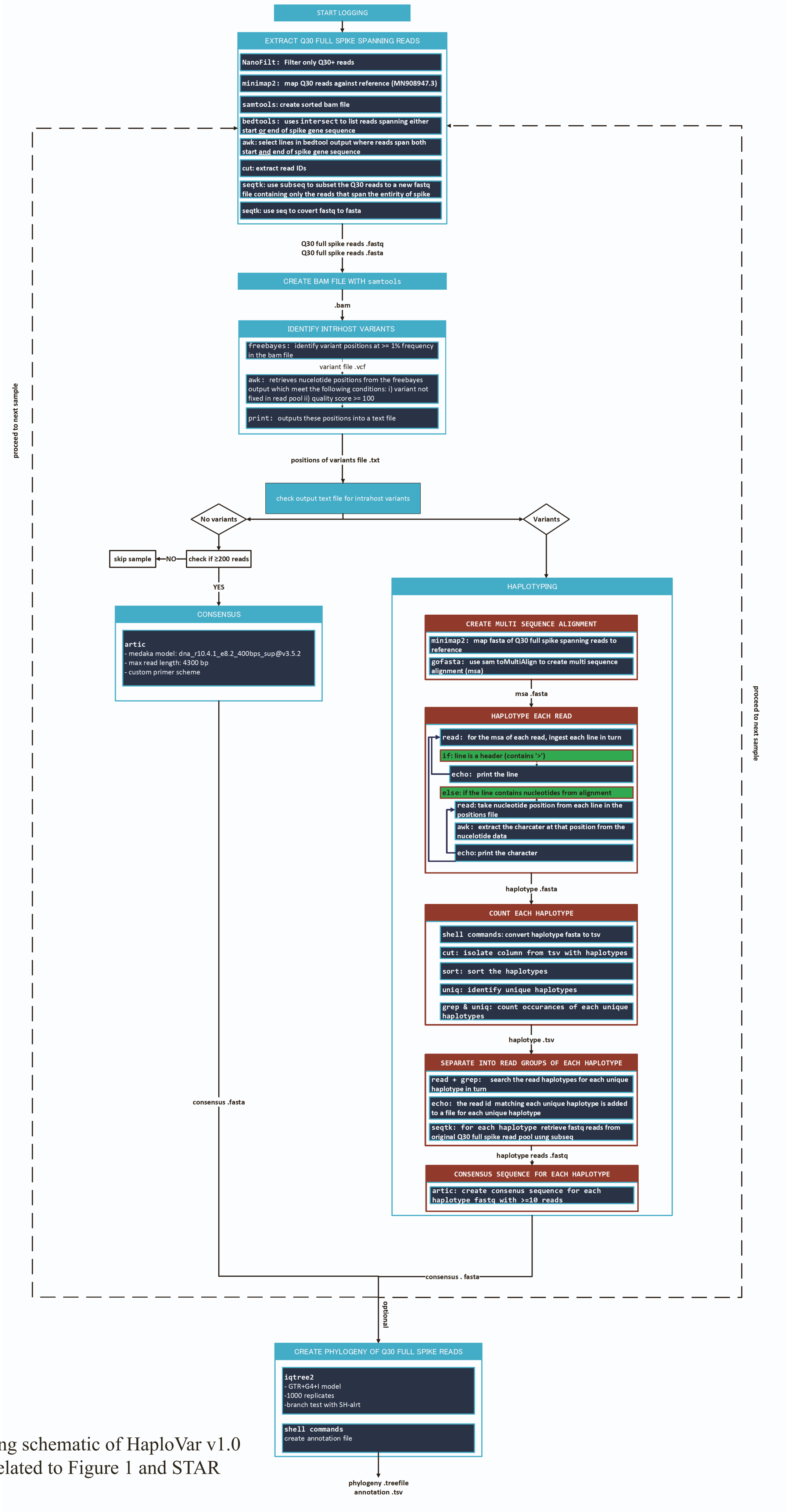

Figure S1. Diagram showing schematic of HaploVar v1.0 bioinformatic workflow. Related to Figure 1 and STAR Methods.

Figure S2

A

Empirical mix; 100,000 viral RNA copies		Mix identified by sequencing					
		Replicate 1		Replicate 2		Replicate 3	
Alpha	Beta	Alpha	Beta	Alpha	Beta	Alpha	Beta
**80%**	**20%**	80.3%	19.7%	75.3%	24.6%	76.9%	23.1%
**90%**	**10%**	88.6%	11.4%	89.7%	10.3%	87.3%	12.7%
**95%**	**5%**	95.0%	5.0%	91.8%	8.2%	95.1%	4.9%
**98%**	**2%**	97.4%	2.6%	97.5%	2.5%	97.4%	2.6%
**99%**	**1%**	99.0%	1.0%	99.2%	0.8%	99.1%	0.9%
**99.5%**	**0.5%**	99.3%	0.7%	99.3%	0.7%	99.2%	0.8%
**100%**	**0%**	not done	not done	100%	0%	99.5%	0.5%

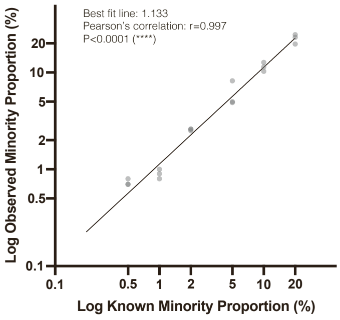

B

Viral Genome RNA copies	Q30 positive full-length spike reads			
	Replicate 1	Replicate 2	Replicate 3	Replicate 4
**1000**	404	4339	4574	293
**500**	3333	1573	4997	1362
**250**	1934	2462	6514	714
**100**	825	3635	0	0

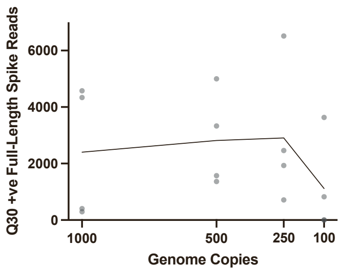

C

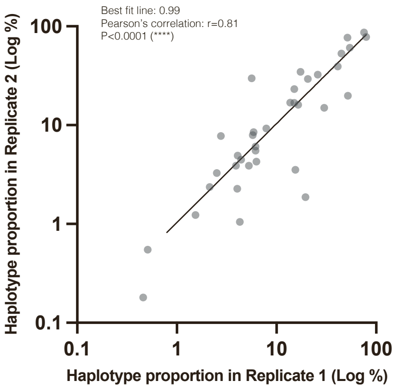

Figure S2. (**A**) Table and logarithmic scatter plot displaying the detection, in triplicate, of pre-defined minority populations of spike haplotypes using the HaploVar 1.0 workflow on empirical mixes containing 10^4 copies of viral RNA from Alpha and Beta lineages. (**B**) Determination of the minimum total number of viral genome RNA copies required to obtain at least 200 Q30-positive full-length reads for the application of HaploVar 1.0. A table and scatter plot illustrate the reproducibility of detecting Q30-positive full-length reads (n=4) in samples with total viral genome RNA copies ranging from 100 to 1,000 (**C**) Logarithmic scatter plot displaying proportion of identified full-spike haplotypes on clinical samples (n=9) in two independent replicates (see also Table S3). Correlations in A and C determined by Pearson's; ****p<0.0001. Related to Figure 1 and STAR Methods.

Figure S3

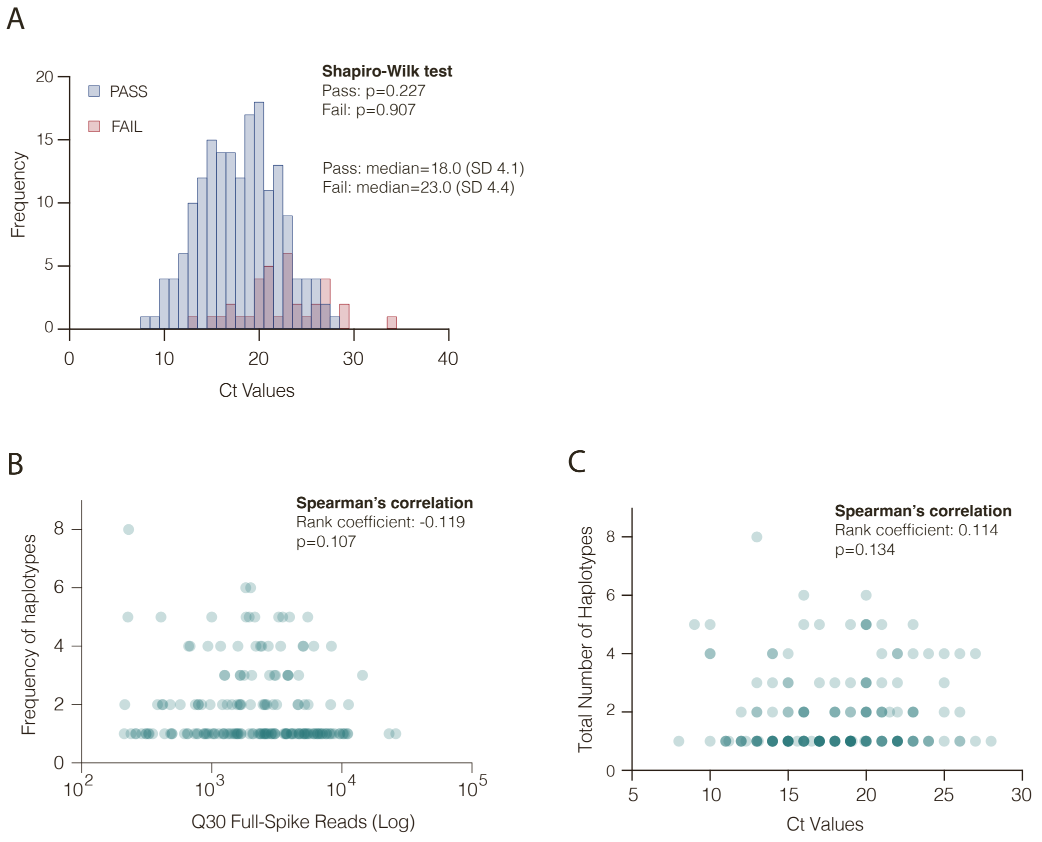

Figure S3. (**A**) Histogram showing Ct value of samples (n=219) successfully haplotyped (blue) compared to those that failed haplotyping (red). Normality of passed and failed samples was tested using Shapiro-Wilk; p>0.05 confirms the hypothesis of normality. (**B, C**) Scatter plots showing number of spike haplotypes with a frequency greater than 5% in each sample used in this study against logarithmic transformed number of Q30 positive full-length spike reads (**B**), or Ct values (**C**). Correlations determined by Spearman's; ns>0.05. Relate with Figure 1.

Figure S4

PATIENTS 1-23

Amino-Acid Matrices representing non-synonymous mutations

Phylogeny Trees fitted with codon-aware model and annotated with amino acid changes on branches

Molecular Clock Trees

Figure S4. (A-W) Amino-acid alignment matrices representing non-synonymous mutations identified in haplotypes from longitudinal samples collected from Patients 1-23 (top panels). Note this reflects the mutations detected at each sampling point, not necessarily the time of their emergence. Maximum likelihood codon divergence phylogeny trees and molecular clock trees of haplotypes collected longitudinally from the same single persistent infections (bottom panels). Related to Figure 2 and Figure S5.

Figure S4A

Patient 1

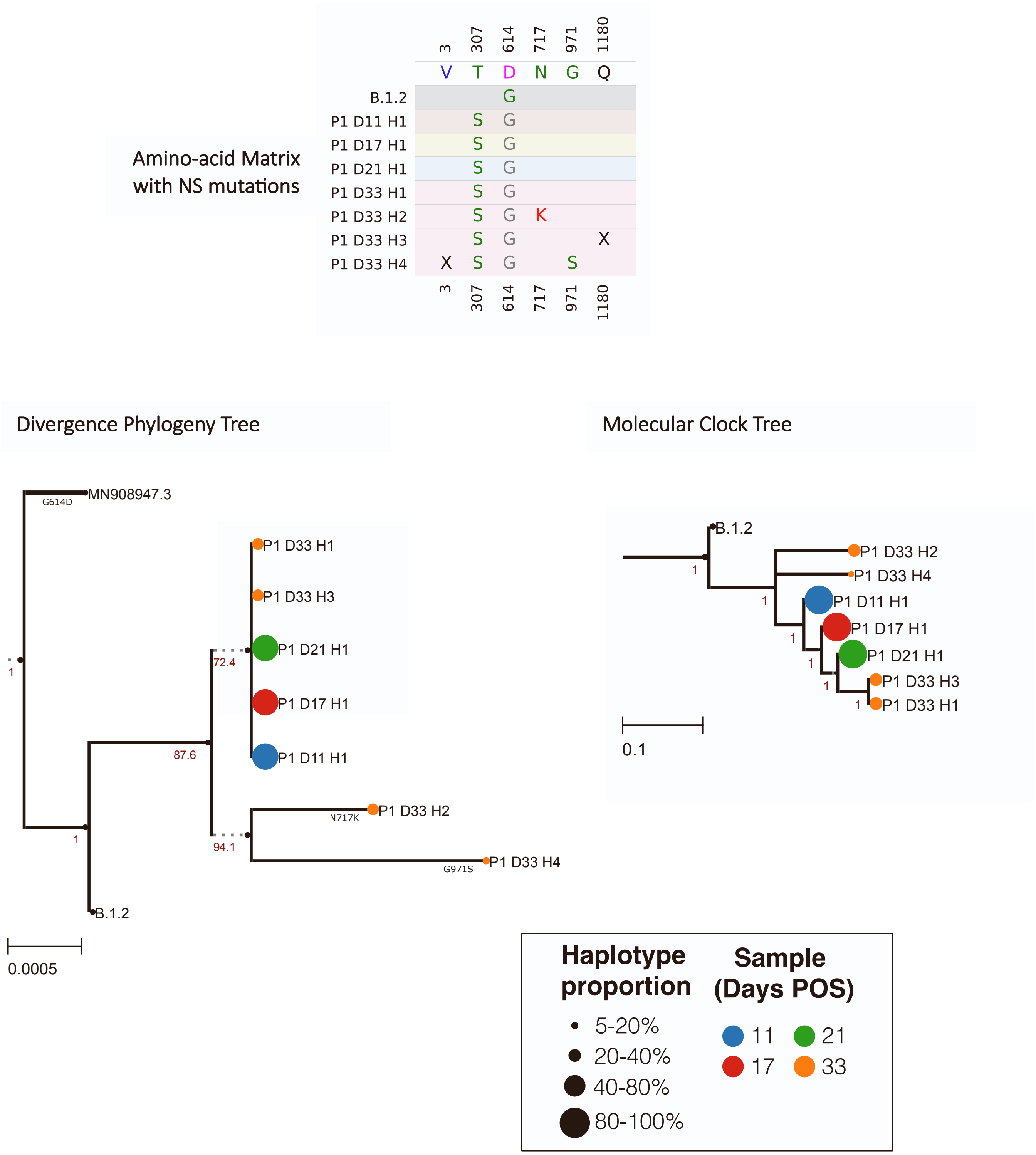

Figure S4B

Patient 2

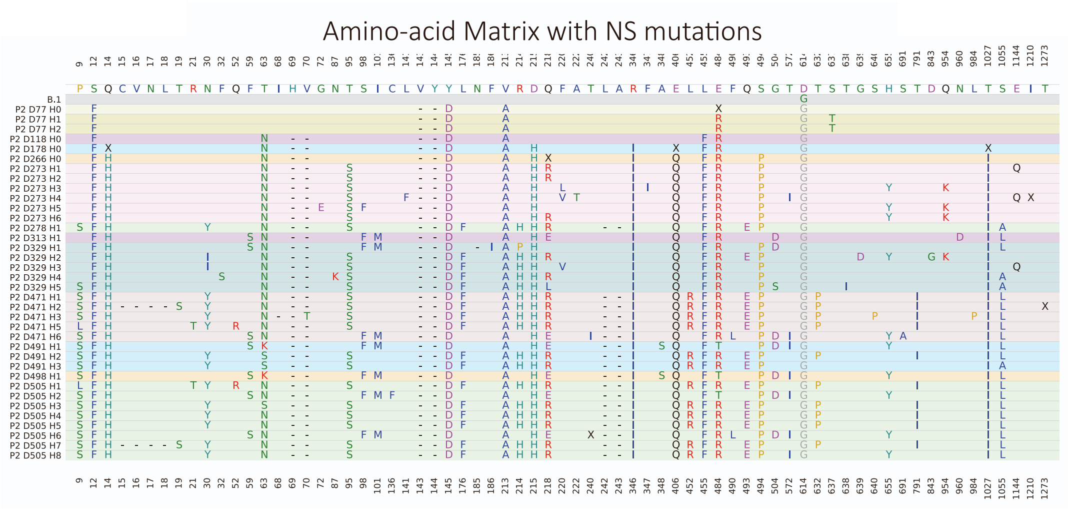

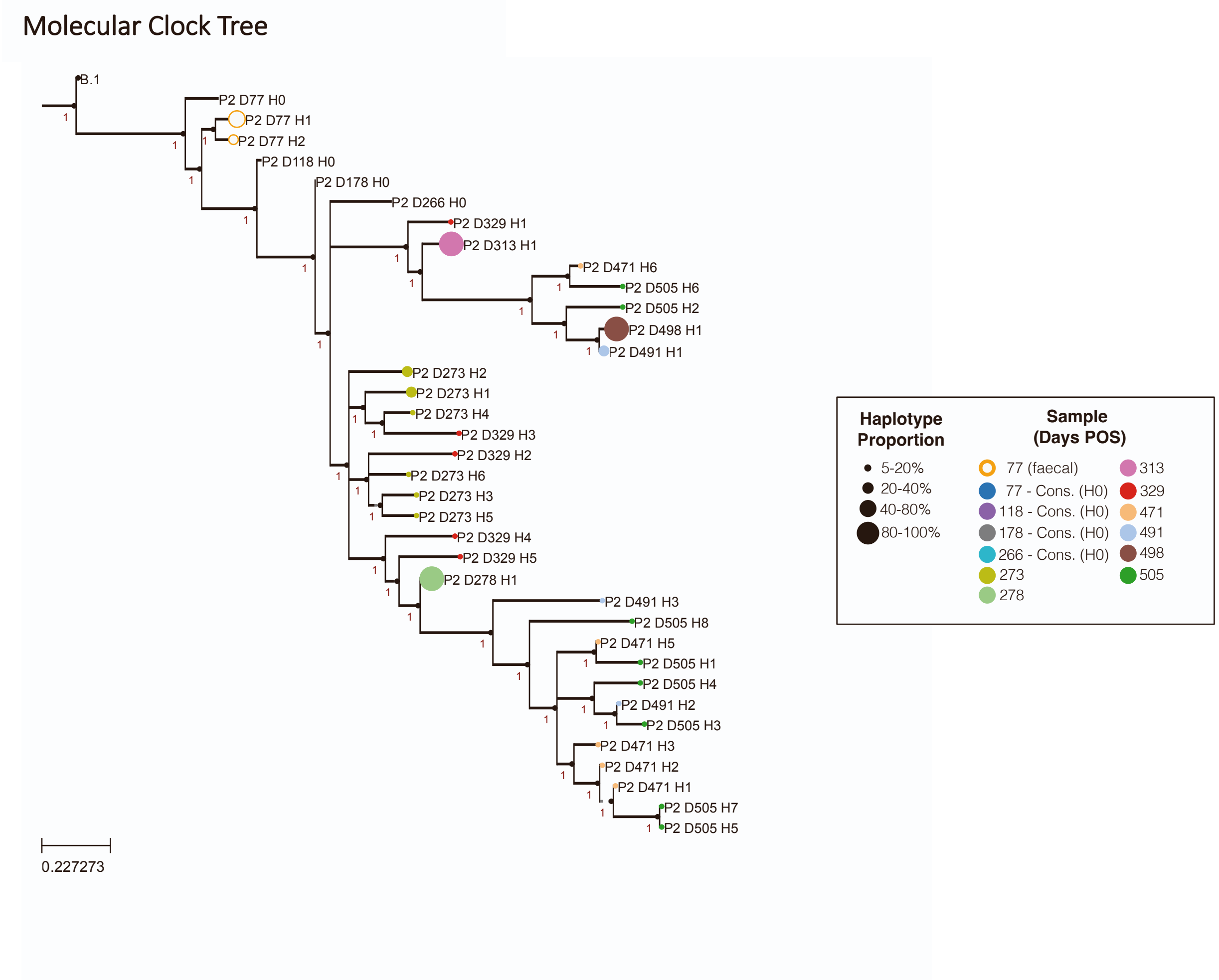

Figure S4B (cont.) – Patient 2

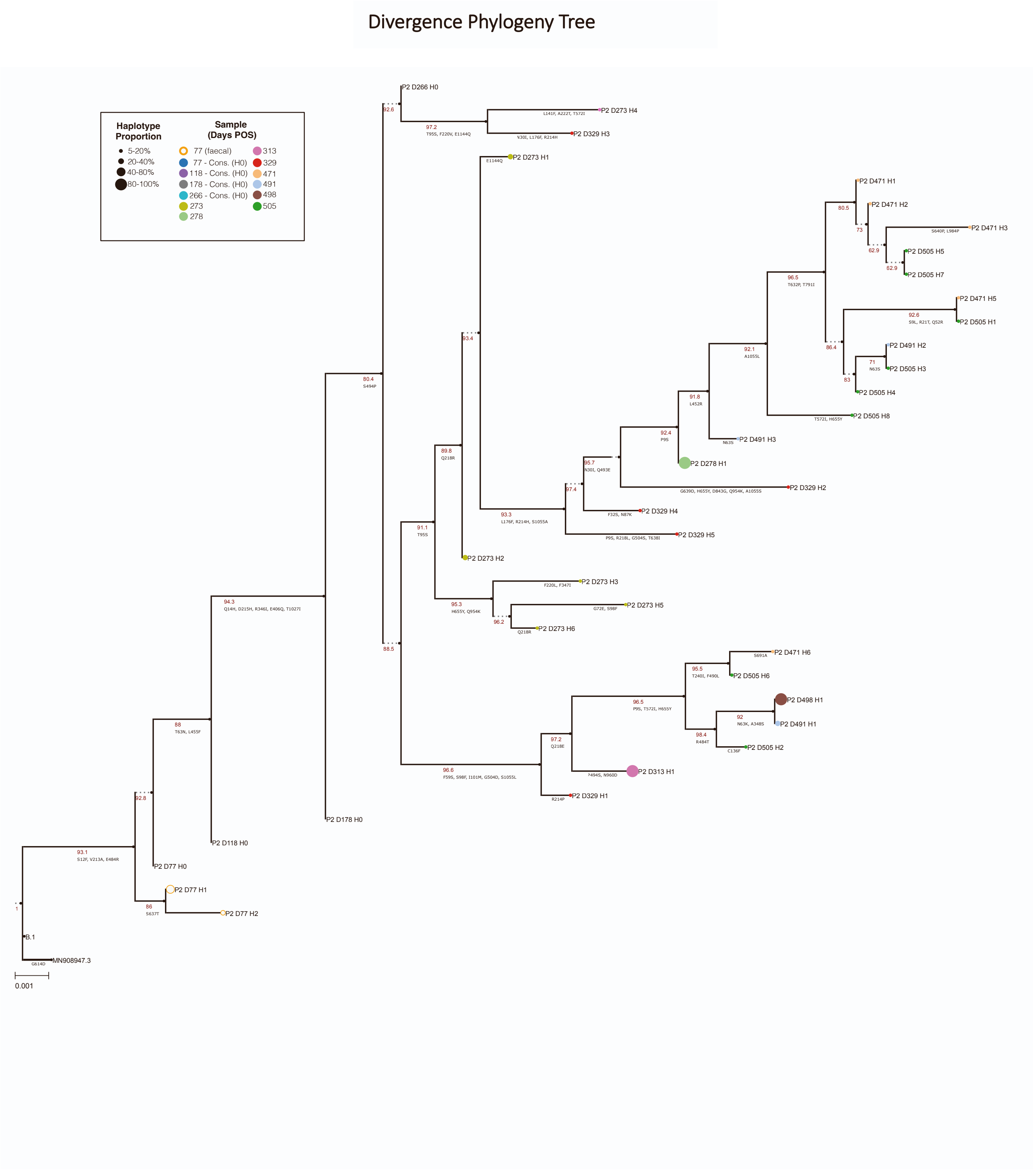

Figure S4C

Patient 3

Amino-acid Matrix with NS mutations

	69	70	243	439	614	932	1027	1253
	H	V	A	N	D	G	T	C
B.1.258					G			
P3 D35 H1	-	-		K	G	D		G
P3 D35 H2	-	-		K	G			G
P3 D35 H3	-	-	V	K	G		I	
P3 D39 H1	-	-		K	G			G
P3 D39 H2	-	-	V	K	G		I	
P3 D39 H3	-	-		K	G			
P3 D39 H4	-	-	V	K	G			G
P3 D39 H5	-	-	V	K	G		I	G
	69	70	243	439	614	932	1027	1253

Divergence Phylogeny Tree

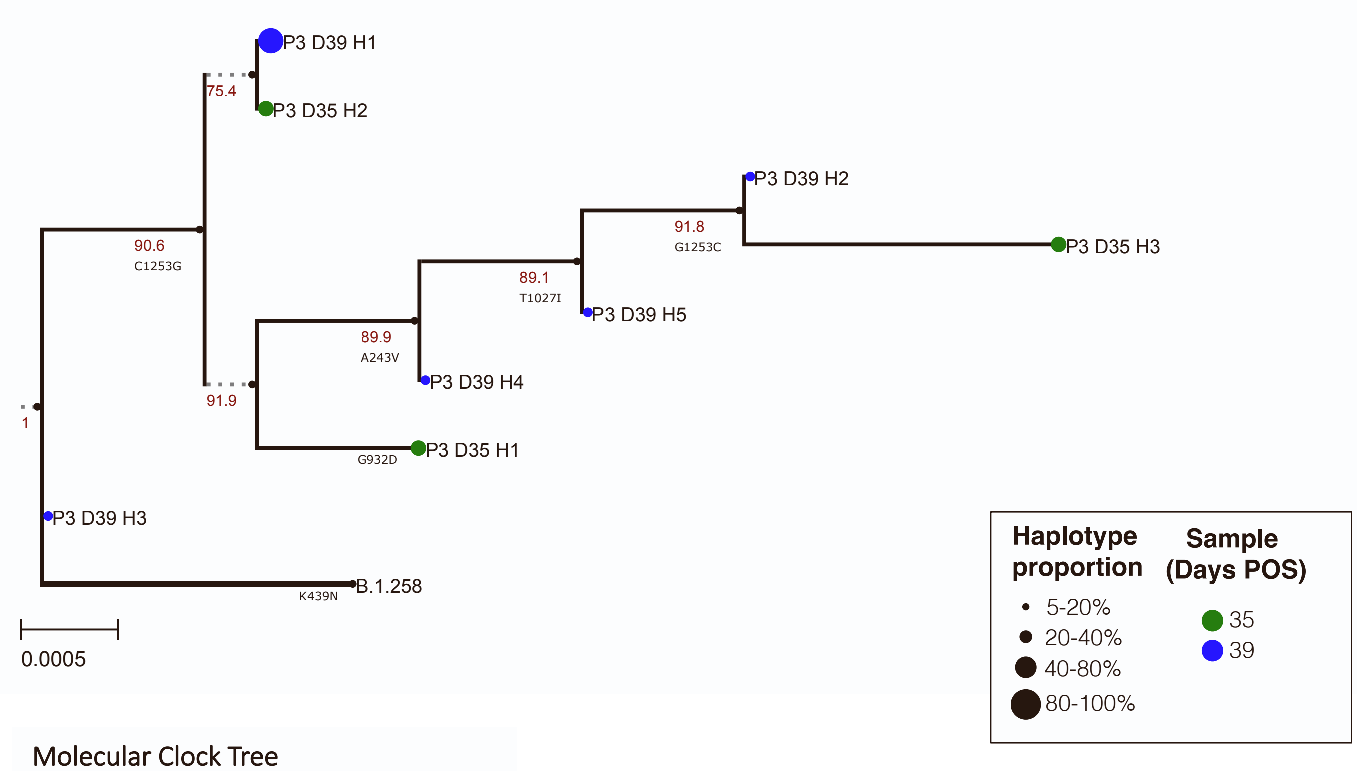

Molecular Clock Tree

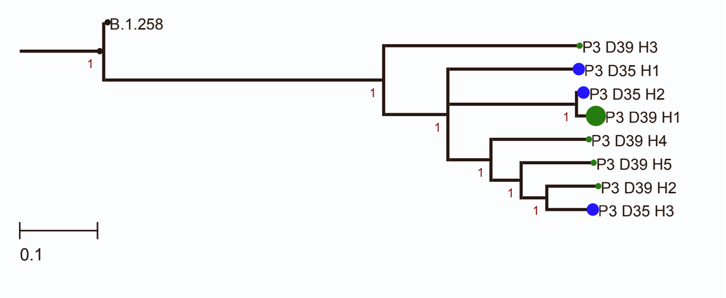

Figure S4D

Patient 4 - 1st infection

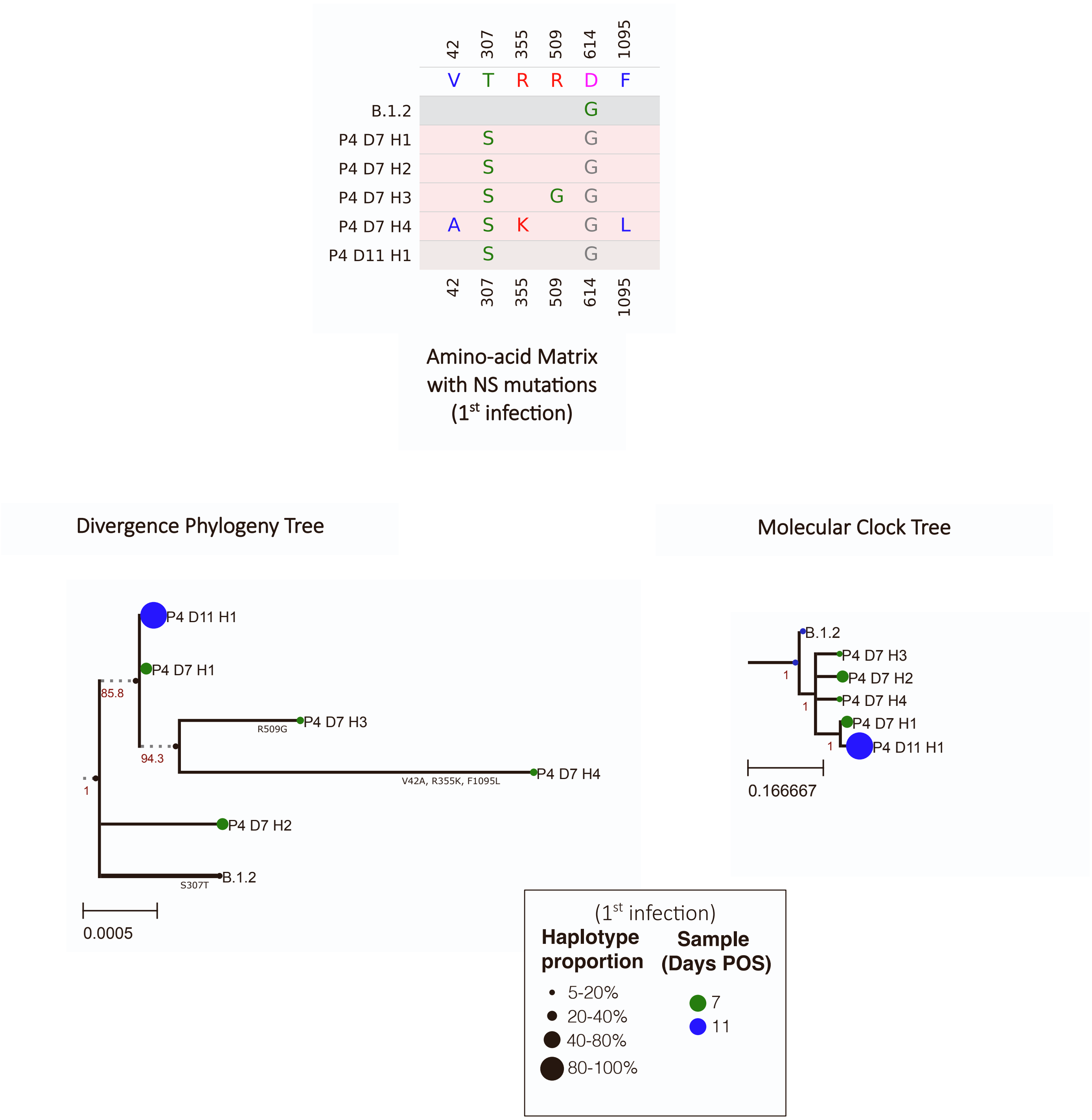

Figure S4D (cont.)

Patient 4 – 2nd infection

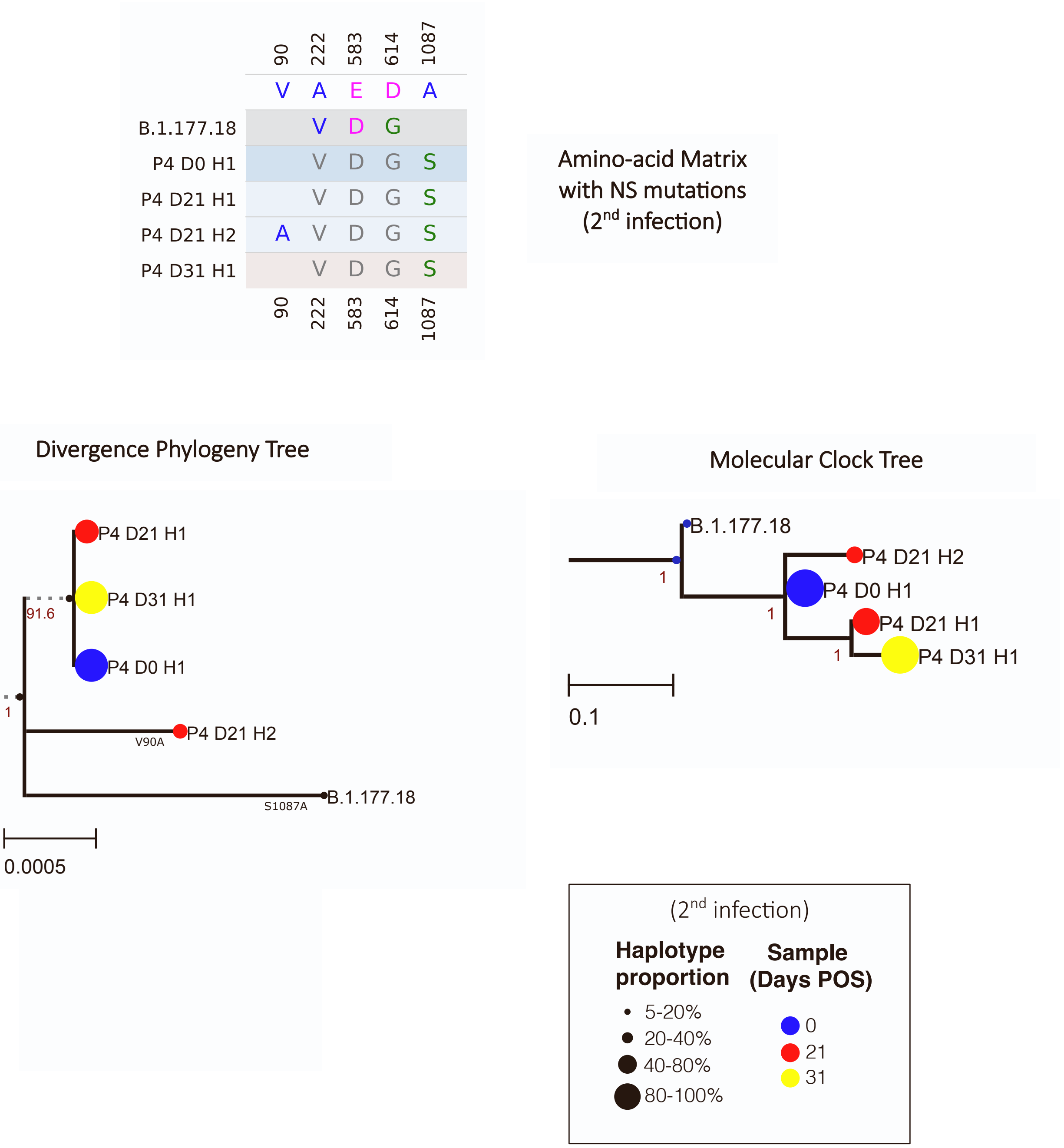

Figure S4E

Patient 5

Amino-acid Matrix with NS mutations

	9	69	70	142	144	152	257	484	501	570	614	681	716	982	1118
	P	H	V	G	Y	W	G	E	N	A	D	P	T	S	D
B.1.1.7		-	-		-				Y	D	G	H	I	A	H
P5 D12 H1		-	-		-				Y	D	G	H	I	A	H
P5 D23 H1		-	-		-				Y	D	G	H	I	A	H
P5 D39 H1		-	-		-				Y	D	G	H	I	A	H
P5 D39 H2	L	-	-		-				Y	D	G	H	I	A	H
P5 D39 H3		-	-		-		S		Y	D	G	H	I	A	H
P5 D48 H1		-	-		-				Y	D	G	H	I	A	H
P5 D58 H1		-	-		-	L		K	Y	D	G	H	I	A	H
P5 D58 H2		-	-		-			K	Y	D	G	H	I	A	H
P5 D64 H1		-	-	-	V			K	Y	D	G	H	I	A	H
P5 D64 H2		-	-	-	V			K	Y	D	G	H	I	A	H
P5 D64 H3		-	-		-				Y	D	G	H	I	A	H
P5 D64 H4		-	-		-			K	Y	D	G	H	I	A	H
P5 D64 H5		-	-	-	V				Y	D	G	H	I	A	H
P5 D69 H1		-	-	-	V			K	Y	D	G	H	I	A	H
P5 D69 H2		-	-	-	V			K	Y	D	G	H	I	A	H
P5 D69 H3		-	-		-				Y	D	G	H	I	A	H
P5 D69 H4		-	-		-			K	Y	D	G	H	I	A	H
P5 D69 H5		-	-	-	V				Y	D	G	H	I	A	H
P5 D71 H1		-	-		-	L		K	Y	D	G	H	I	A	H
	9	69	70	142	144	152	257	484	501	570	614	681	716	982	1118

Divergence Phylogeny Tree

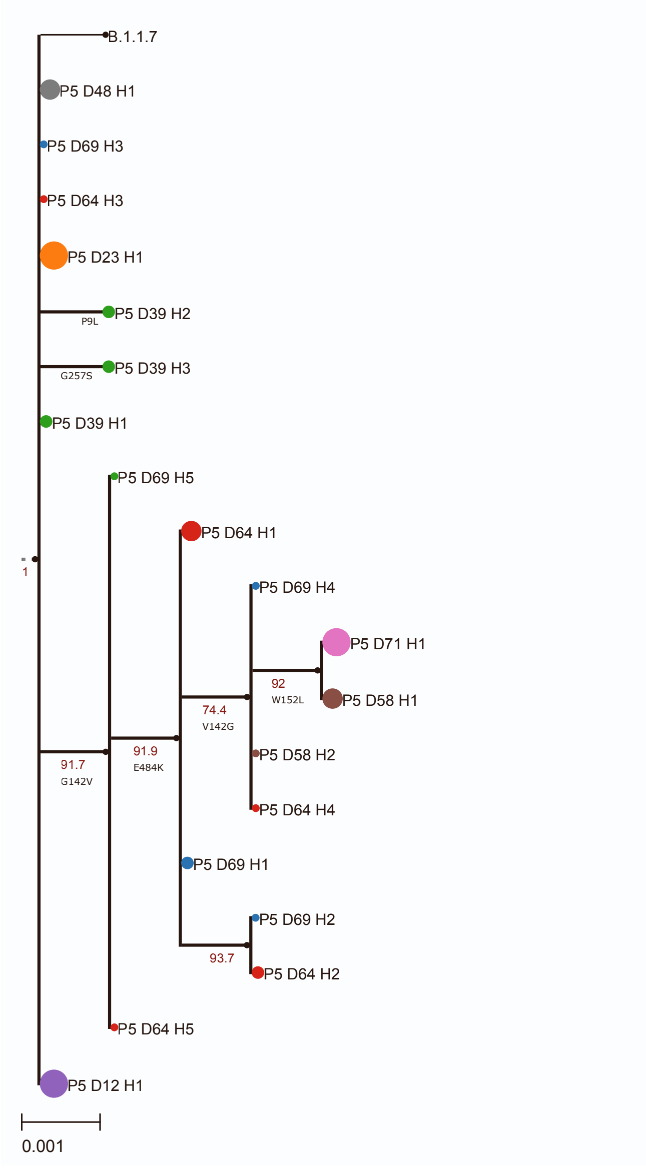

Molecular Clock Tree

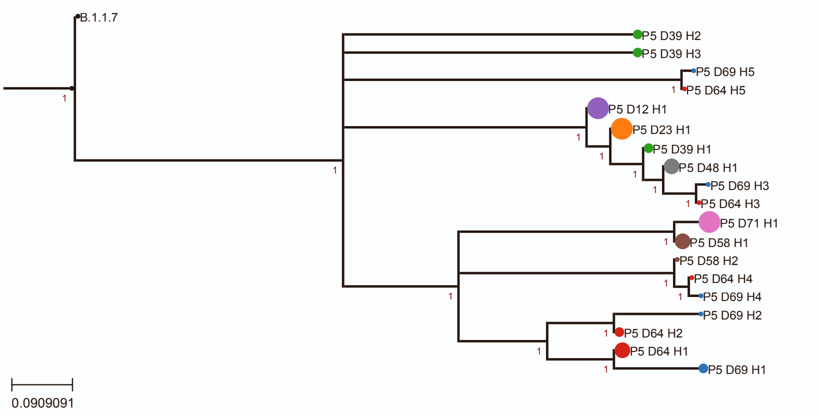

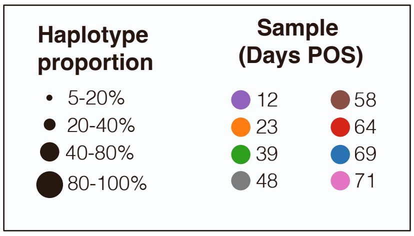

Figure S4F

Patient 6

Amino-acid Matrix with NS mutations

	64	67	69	70	95	144	215	501	570	595	614	681	684	716	982	1118
	W	A	H	V	T	Y	D	N	A	V	D	P	A	T	S	D
B.1.1.7			-	-		-		Y	D		G	H		I	A	H
P6 D16 H1			-	-		-		Y	D		G	H		I	A	H
P6 D38 H1	R		-	-		-		Y	D		G	H	V	I	A	H
P6 D38 H2	R		-	-		-		Y	D		G	H		I	A	H
P6 D38 H3			-	-	I	-		Y	D		G	H		I	A	H
P6 D38 H4			-	-		-		Y	D		G	H		I	A	H
P6 D46 H1		V	-	-		-	G	Y	D	A	G	H		I	A	H
P6 D48 H1			-	-	I	-		Y	D		G	H		I	A	H
P6 D48 H2			-	-		-		Y	D		G	H		I	A	H
	64	67	69	70	95	144	215	501	570	595	614	681	684	716	982	1118

Divergence Phylogeny Tree

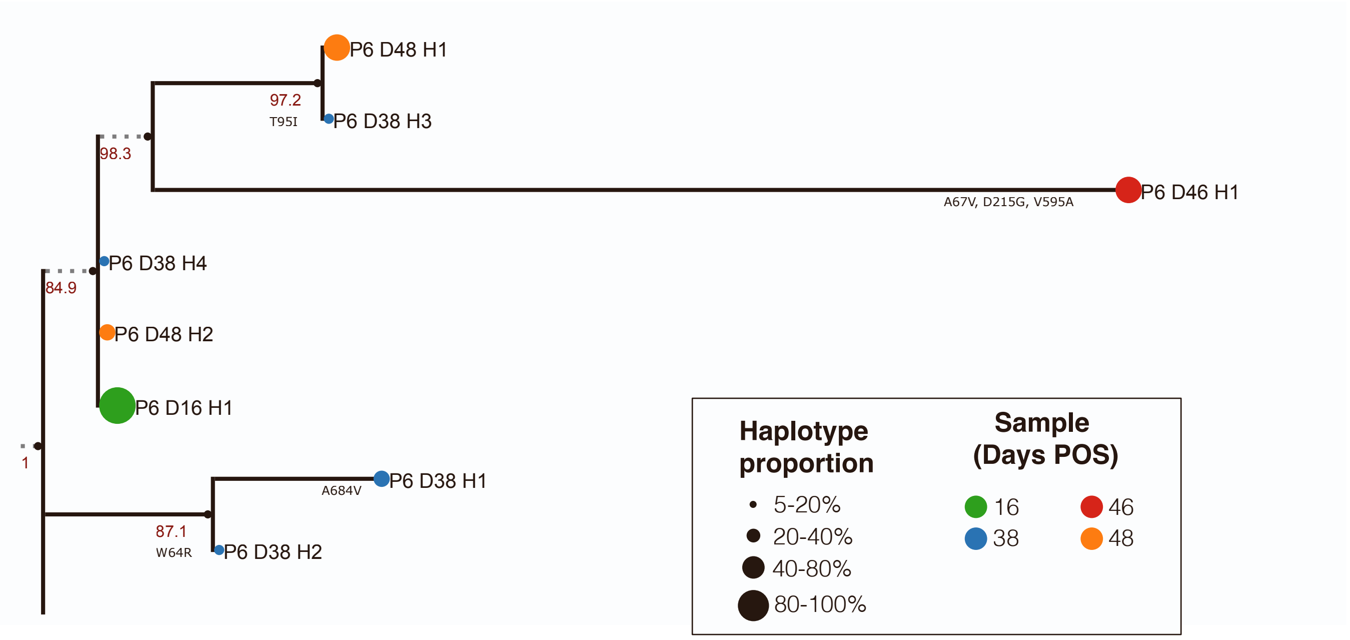

Molecular Clock Tree

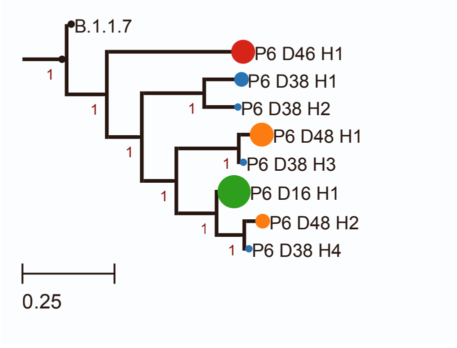

Figure S4G

Patient 7

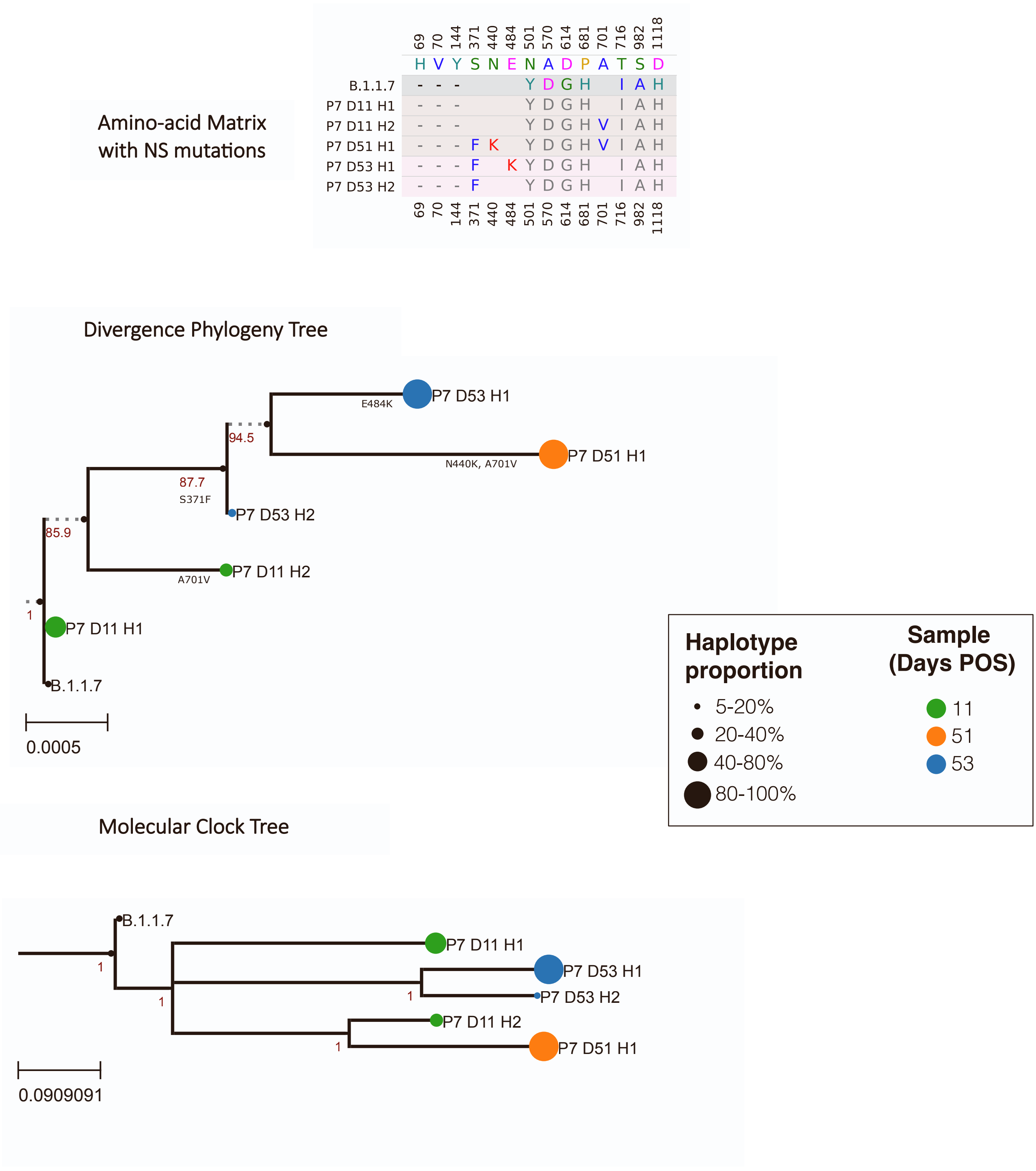

Figure S4H

Patient 8

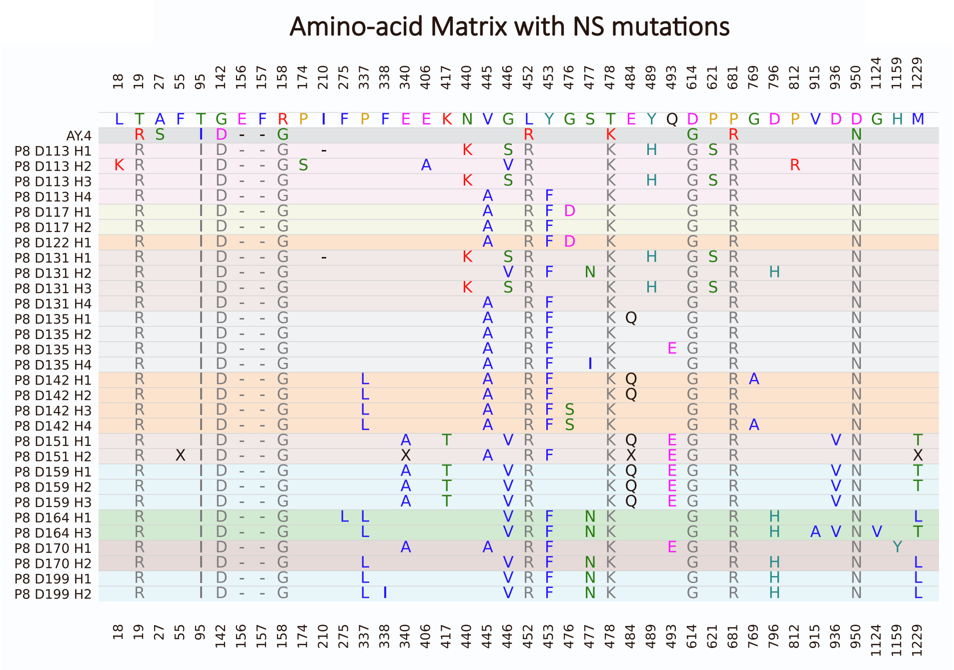

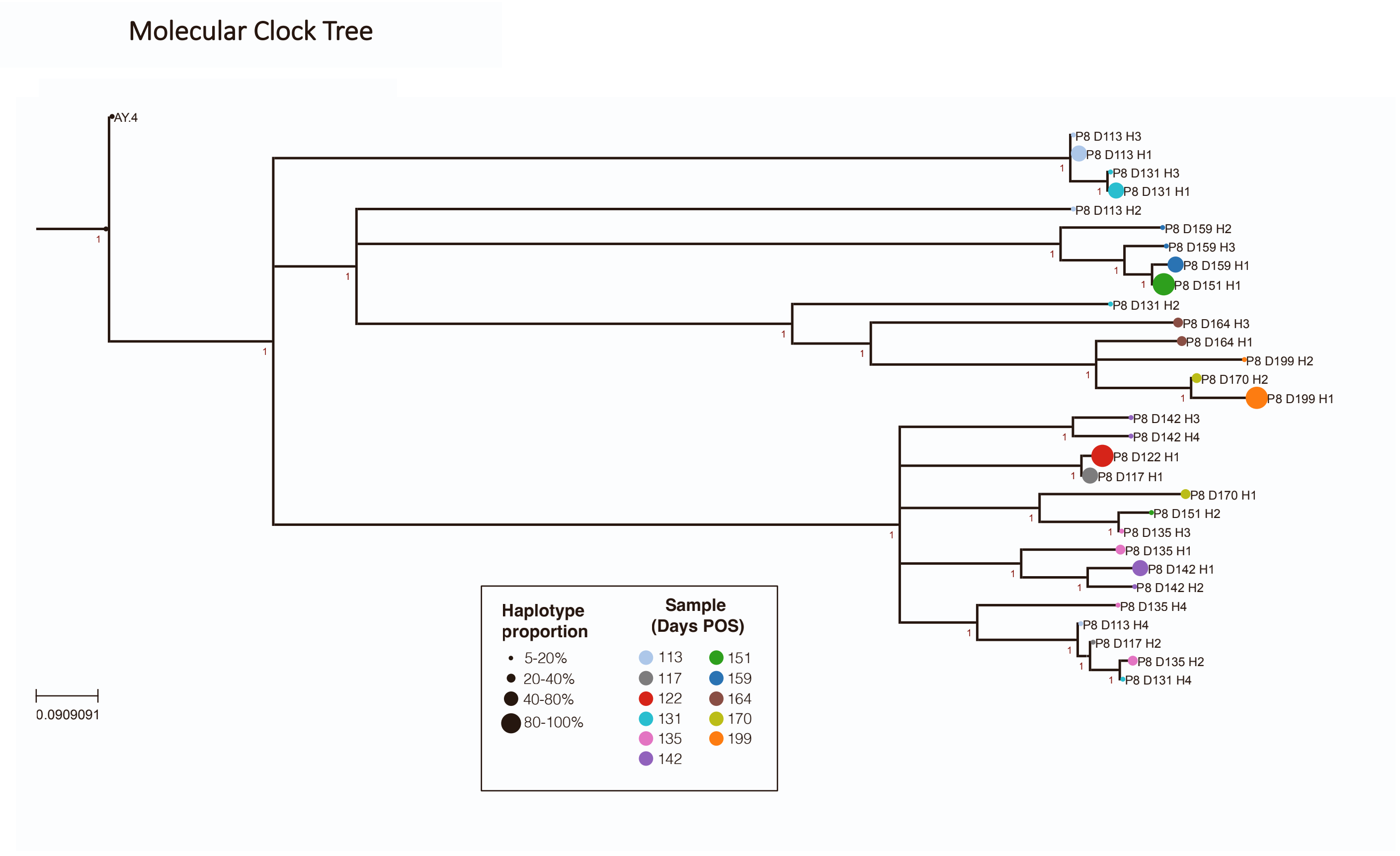

Figure S4H (cont.)

Patient 8

Divergence Phylogeny Tree

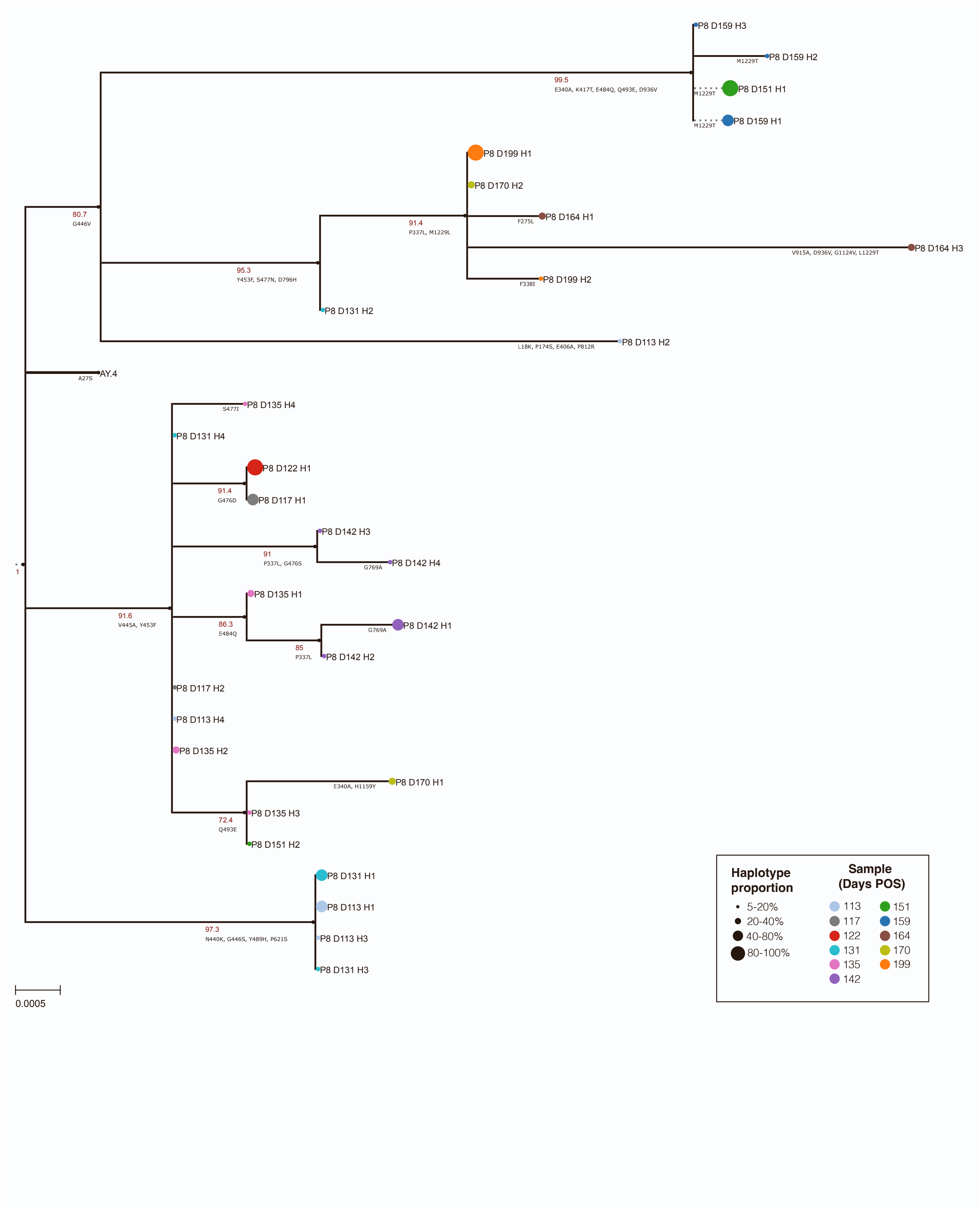

Figure S4I

Patient 9

Amino-acid Matrix with NS mutations

	67	69	70	95	142	143	144	145	211	212	337	339	340	346	367	371	373	375	417	440	446	460	477	478	484	493	496	498	501	505	547	614	655	679	681	764	796	856	954	969	981
	A	H	V	T	G	V	Y	Y	N	L	P	G	E	R	V	S	S	S	K	N	G	N	S	T	E	Q	G	Q	N	Y	T	D	H	N	P	N	D	N	Q	N	L
BA.1.1	V	-	-	I	-	-	-	D	-	I		D		K						K	S		N	K	A	R	S	R	Y	H	K	G	Y	K	H	K	Y	K	H	K	F
P9 D16 H1	V	-	-	I	-	-	-	D	-	I		D	D	K		L	P	F	N	K	S		N	K	A	R	S	R	Y	H	K	G	Y	K	H	K	Y	K	H	K	F
P9 D16 H2	V	-	-	I	-	-	-	D	-	I	S	D		K		L	P	F	N	K	S		N	K	A	R	S	R	Y	H	K	G	Y	K	H	K	Y	K	H	K	F
P9 D16 H3	V	-	-	I	-	-	-	D	-	I		D		K		L	P	F	N	K	S		N	K	A	R	S	R	Y	H	K	G	Y	K	H	K	Y	K	H	K	F
P9 D24 H1	V	-	-	I	-	-	-	D	-	I	S	D		K		L	P	F	N	K	S		N	K	A	R	S	R	Y	H	K	G	Y	K	H	K	Y	K	H	K	F
P9 D24 H2	V	-	-	I	-	-	-	D	-	I		D	D	K		L	P	F	N	K	S		N	K	A	R	S	R	Y	H	K	G	Y	K	H	K	Y	K	H	K	F
P9 D24 H3	V	-	-	I	-	-	-	D	-	I	S	D	D	K		L	P	F	N	K	S		N	K	A	R	S	R	Y	H	K	G	Y	K	H	K	Y	K	H	K	F
P9 D30 H1	V	-	-	I	-	-	-	D	-	I		D	D	K		L	P	F	N	K	S		N	K	A	R	S	R	Y	H	K	G	Y	K	H	K	Y	K	H	K	F
P9 D30 H2	V	-	-	I	-	-	-	D	-	I	S	D		K		L	P	F	N	K	S		N	K	A	R	S	R	Y	H	K	G	Y	K	H	K	Y	K	H	K	F
P9 D30 H3	V	-	-	I	-	-	-	D	-	I	S	D	D	K		L	P	F	N	K	S		N	K	A	R	S	R	Y	H	K	G	Y	K	H	K	Y	K	H	K	F
P9 D40 H1	V	-	-	I	-	-	-	D	-	I	S	D		K		L	P	F	N	K	S	K	N	K	A	R	S	R	Y	H	K	G	Y	K	H	K	Y	K	H	K	F
P9 D40 H2	V	-	-	I	-	-	-	D	-	I	S	D		K		L	P	F	N	K	S		N	K	A	R	S	R	Y	H	K	G	Y	K	H	K	Y	K	H	K	F
P9 D40 H3	V	-	-	I	-	-	-	D	-	I	S	D		K	F	L	P	F	N	K	S		N	K	A	R	S	R	Y	H	K	G	Y	K	H	K	Y	K	H	K	F
P9 D40 H4	V	-	-	I	-	-	-	D	-	I		D	D	K		L	P	F	N	K	S		N	K	A	R	S	R	Y	H	K	G	Y	K	H	K	Y	K	H	K	F
P9 D43 H1	V	-	-	I	-	-	-	D	-	I		D	D	K		L	P	F	N	K	S		N	K	A	R	S	R	Y	H	K	G	Y	K	H	K	Y	K	H	K	F
P9 D43 H2	V	-	-	I	-	-	-	D	-	I	S	D		K		L	P	F	N	K	S	K	N	K	A	R	S	R	Y	H	K	G	Y	K	H	K	Y	K	H	K	F
P9 D45 H1	V	-	-	I	-	-	-	D	-	I		D	D	K		L	P	F	N	K	S		N	K	A	R	S	R	Y	H	K	G	Y	K	H	K	Y	K	H	K	F
P9 D45 H2	V	-	-	I	-	-	-	D	-	I		D	D	K		L	P	F	N	K	S		N	K	A	R	S	R	Y	H	K	G	Y	K	H	E	Y	K	H	K	F
P9 D74 H1	V	-	-	I	-	-	-	D	-	I		D	D	K		L	P	F	T	K	S		N	K	A	R	S	R	Y	H	K	G	Y	K	H	K	Y	K	H	K	F
	67	69	70	95	142	143	144	145	211	212	337	339	340	346	367	371	373	375	417	440	446	460	477	478	484	493	496	498	501	505	547	614	655	679	681	764	796	856	954	969	981

Molecular Clock Tree

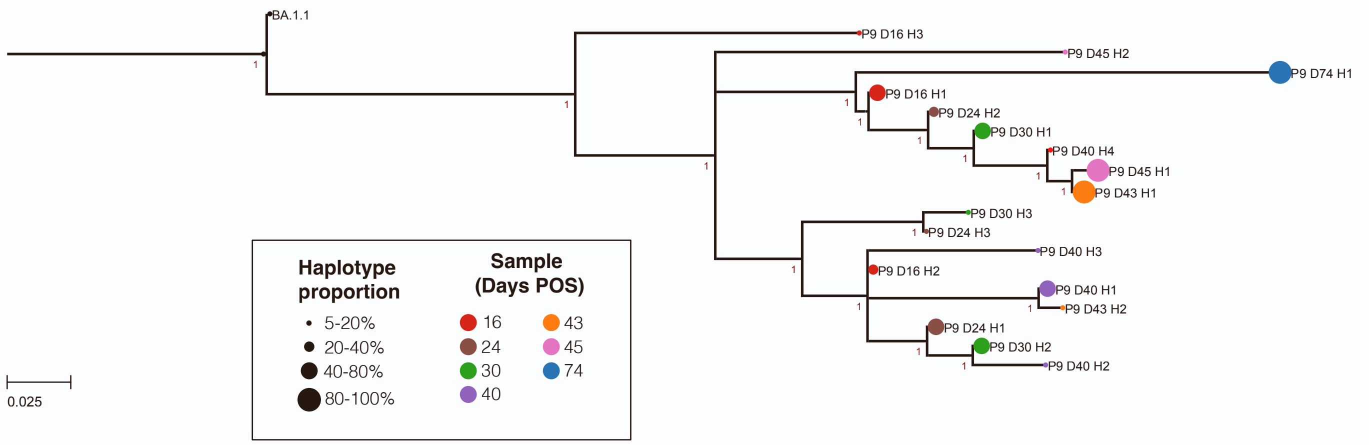

Figure S4I (cont.)

Patient 9

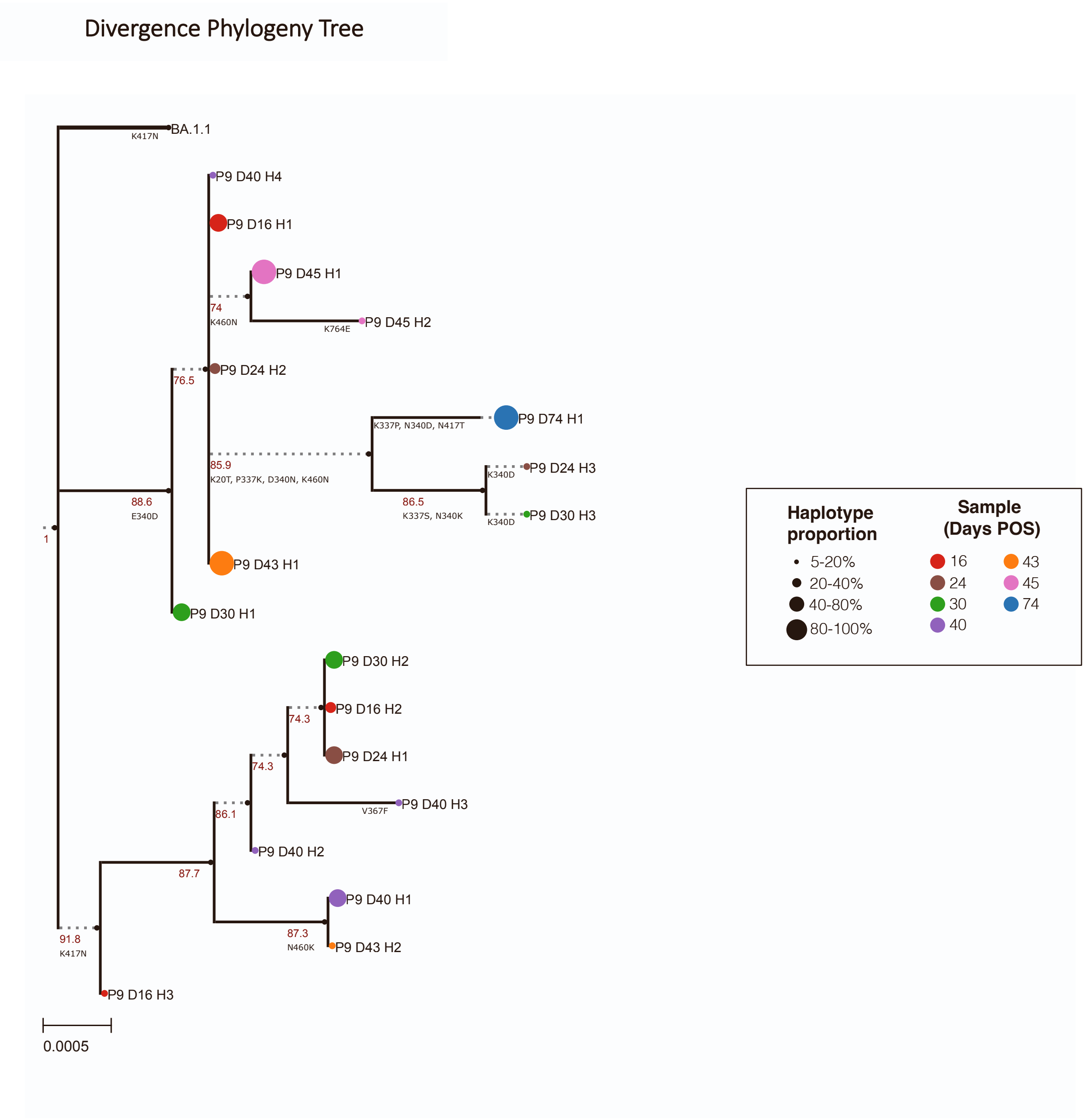

Figure S4J

Patient 10

Amino-acid Matrix with NS mutations

	19	24	25	26	27	142	213	339	340	356	371	373	375	376	385	405	408	417	440	477	478	484	493	498	501	505	614	655	679	681	764	796	954	969
	T	L	P	P	A	G	V	G	E	K	S	S	S	T	T	D	R	K	N	S	T	E	Q	Q	N	Y	D	H	N	P	N	D	Q	N
BA.2	I	-	-	-	S	D	G	D			F	P	F	A		N	S	N	K	N	K	A	R	R	Y	H	G	Y	K	H	K	Y	H	K
P10 D1 H1	I	-	-	-	S	D	G	D			F	P	F	A		N	S	N	K	N	K	A	R	R	Y	H	G	Y	K	H	K	Y	H	K
P10 D10 H1	I	-	-	-	S	D	G	D			F	P	F	A		N	S	N	K	N	K	A	R	R	Y	H	G	Y	K	H	K	Y	H	K
P10 D15 H1	I	-	-	-	S	D	G	D			F	P	F	A		N	S	N	K	N	K	A	R	R	Y	H	G	Y	K	H	K	Y	H	K
P10 D37 H1	I	-	-	-	S	D	G	D			F	P	F	A		N	S	N	K	N	K	A	R	R	Y	H	G	Y	K	H	K	Y	H	K
P10 D43 H1	I	-	-	-	S	D	G	D			F	P	F	A		N	S	N	K	N	K	A	R	R	Y	H	G	Y	K	H	K	Y	H	K
P10 D43 H2	I	-	-	-	S	D	G	D			F	P	F	A	I	N	S	N	K	N	K	A	R	R	Y	H	G	Y	K	H	K	Y	H	K
P10 D43 H3	I	-	-	-	S	D	G	D			F	P	F	A	I	N	S	N	K	N	K	A	R	R	Y	H	G	Y	K	H	K	Y	H	K
P10 D56 H1	I	-	-	-	S	D	G	D			F	P	F	A		N	S	N	K	N	K	A	R	R	Y	H	G	Y	K	H	K	Y	H	K
P10 D56 H2	I	-	-	-	S	D	G	D		T	F	P	F			N	S	N	K	N	K	A	R	R	Y	H	G	Y	K	H	K	Y	H	K
P10 D72 H1	I	-	-	-	S	D	G	D	A		F	P	F	A		N	S	I	K	N	K	A	R	R	Y	H	G	Y	K	H	K	Y	H	K
	19	24	25	26	27	142	213	339	340	356	371	373	375	376	385	405	408	417	440	477	478	484	493	498	501	505	614	655	679	681	764	796	954	969

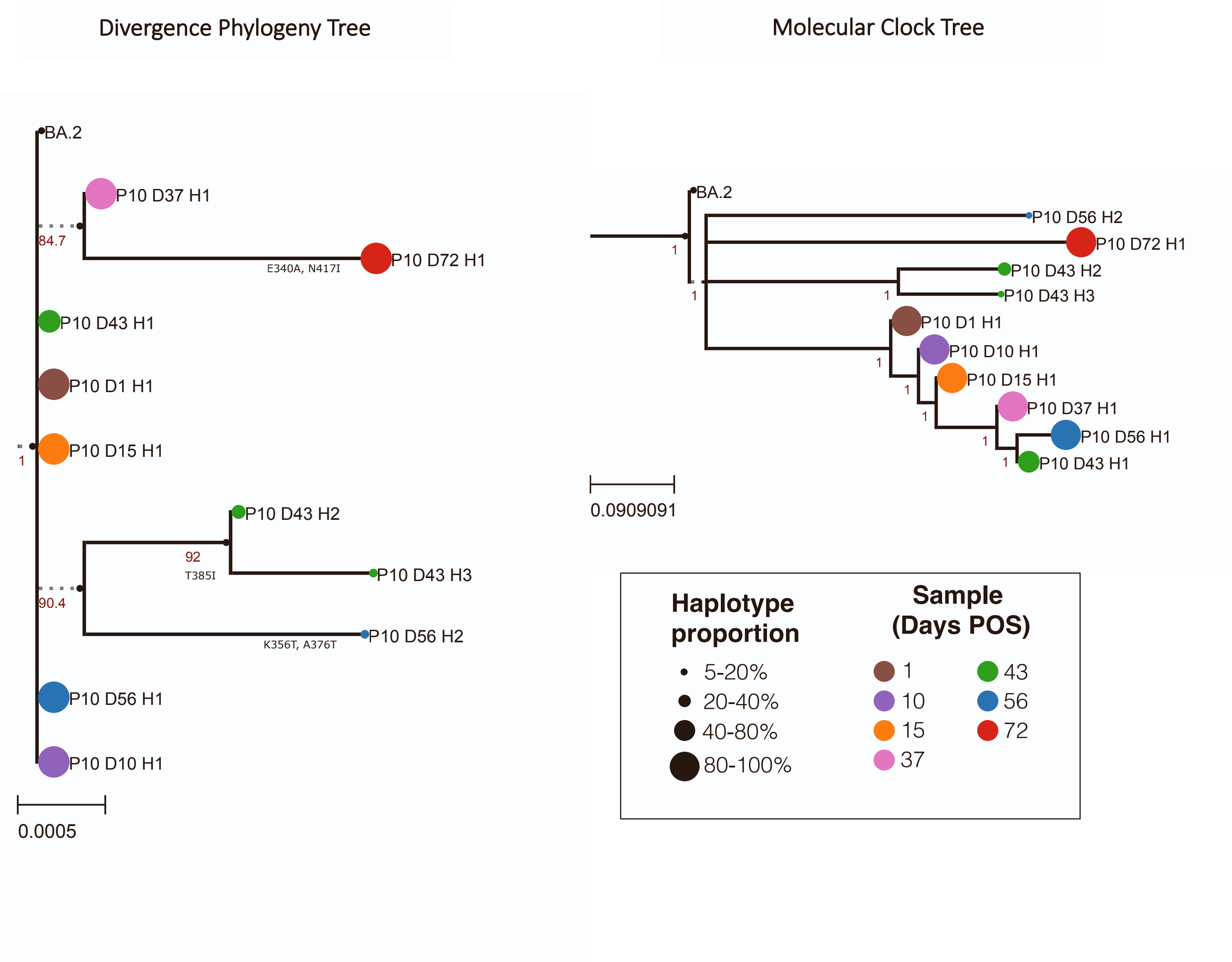

Figure S4K

Patient 11

Amino-acid Matrix with NS mutations

	67	69	70	95	142	143	144	145	211	212	339	346	371	373	375	417	440	446	477	478	484	493	496	498	501	505	547	614	655	679	681	764	796	856	936	954	961	969	981	987
	A	H	V	T	G	V	Y	Y	N	L	G	R	S	S	S	K	N	G	S	T	E	Q	G	Q	N	Y	T	D	H	N	P	N	D	N	D	Q	T	N	L	V
BA.1.1	V	-	-	I	-	-	-	D	-	I	D	K					K	S	N	K	A	R	S	R	Y	H	K	G	Y	K	H	K	Y	K		H		K	F	
P11 D129 H1	V	-	-	I	-	-	-	D	-	I	D	K	L	P	F	N	K	S	N	K	A	R	S	R	Y	H	K	G	Y	K	H	K	Y	K	Y	H		K	F	F
P11 D129 H2	V	-	-	I	-	-	-	D	-	I	D	K	L	P	F	N	K	S	N	K	A	R	S	R	Y	H	K	G	Y	K	H	K	Y	K		H		K	F	F
P11 D129 H3	V	-	-	I	-	-	-	D	-	I	D	K	L	P	F	N	K	S	N	K	A	R	S	R	Y	H	K	G	Y	K	H	K	Y	K		H		K	F	
P11 D129 H4	V	-	-	I	-	-	-	D	-	I	D	K	I	P	F	N	K	S	N	K	A	R	S	R	Y	H	K	G	Y	K	H	K	Y	K		H	M	K	F	F
P11 D129 H5	V	-	-	I	-	-	-	D	-	I	D	K	L	P	F	N	K	S	N	K	A	R	S	R	Y	H	K	G	Y	K	H	K	Y	K	Y	H		K	F	
	67	69	70	95	142	143	144	145	211	212	339	346	371	373	375	417	440	446	477	478	484	493	496	498	501	505	547	614	655	679	681	764	796	856	936	954	961	969	981	987

Divergence Phylogeny Tree

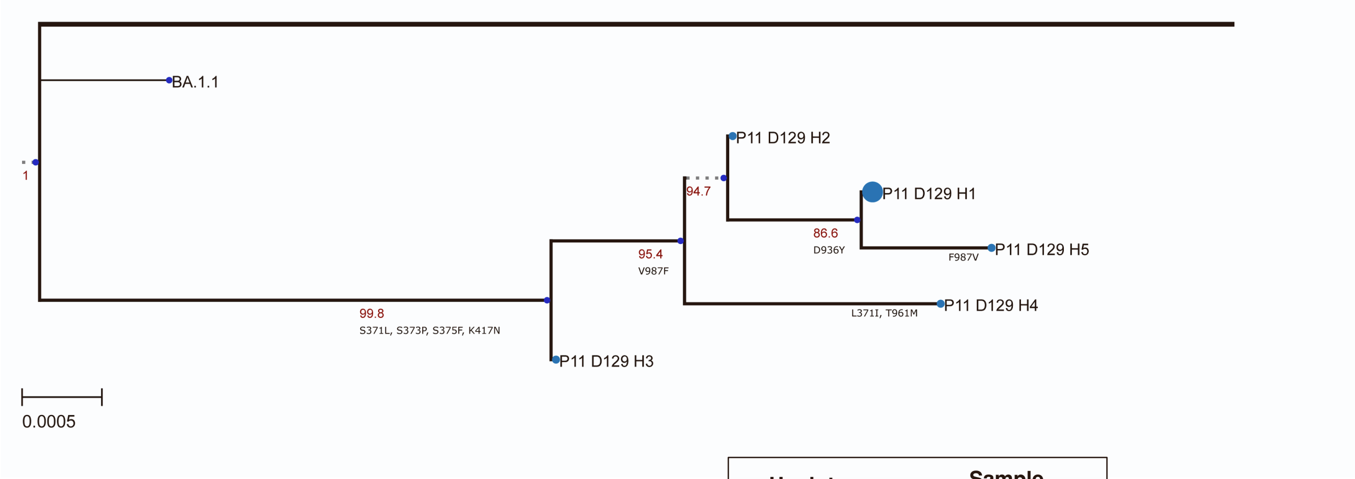

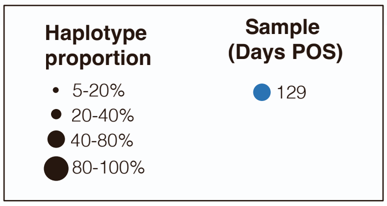

Molecular Clock Tree

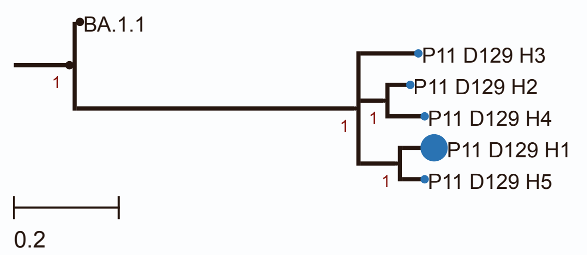

Figure S4L

Patient 12

Amino-acid Matrix with NS mutations

	19	24	25	26	27	142	213	339	340	356	371	373	375	376	385	405	408	417	440	477	478	484	493	498	501	505	614	655	679	681	764	796	954	969
	T	L	P	P	A	G	V	G	E	K	S	S	S	T	T	D	R	K	N	S	T	E	Q	Q	N	Y	D	H	N	P	N	D	Q	N
BA.2	I	-	-	-	S	D	G	D			F	P	F	A		N	S	N	K	N	K	A	R	R	Y	H	G	Y	K	H	K	Y	H	K
P12 D0 H1	I	-	-	-	S	D	G	D			F	P	F	A		N	S	N	K	N	K	A	R	R	Y	H	G	Y	K	H	K	Y	H	K
P12 D1 H1	I	-	-	-	S	D	G	D			F	P	F	A		N	S	N	K	N	K	A	R	R	Y	H	G	Y	K	H	K	Y	H	K
P12 D2 H1	I	-	-	-	S	D	G	D	K		F	P	F	A		N	S	N	K	N	K	A	R	R	Y	H	G	Y	K	H	K	Y	H	K
P12 D24 H1	I	-	-	-	S	D	G	D	Q		F	P	F	A	I	N	S	N	K	N	K	A	R	R	Y	H	G	Y	K	H	K	Y	H	K
P12 D55 H1	I	-	-	-	S	D	G	D	K		F	P	F	A		N	S	N	K	N	K	A	R	R	Y	H	G	Y	K	H	K	Y	H	K
P12 D55 H2	I	-	-	-	S	D	G	D	Q		F	P	F	A		N	S	N	K	N	K	A	R	R	Y	H	G	Y	K	H	K	Y	H	K
P12 D94 H1	I	-	-	-	S	D	G	D	Q		F	P	F	A	I	N	S	N	K	N	K	A	R	R	Y	H	G	Y	K	H	K	Y	H	K
P12 D113 H1	I	-	-	-	S	D	G	D		R	F	P	F	A		N	S	N	K	N	K	A	R	R	Y	H	G	Y	K	H	K	Y	H	K
P12 D113 H2	I	-	-	-	S	D	G	D			F	P	F	A		N	S	N	K	N	K	A	R	R	Y	H	G	Y	K	H	K	Y	H	K
P12 D117 H1	I	-	-	-	S	D	G	D			F	P	F	A		N	S	N	K	N	K	A	R	R	Y	H	G	Y	K	H	K	Y	H	K
P12 D117 H2	I	-	-	-	S	D	G	D			F	P	F	A	I	N	S	N	K	N	K	A	R	R	Y	H	G	Y	K	H	K	Y	H	K
P12 D117 H3	I	-	-	-	S	D	G	D			F	P	F	A	I	N	S	N	K	N	K	A	R	R	Y	H	G	Y	K	H	K	Y	H	K

19 24 25 26 27 142 213 339 340 356 371 373 375 376 385 405 408 417 440 477 478 484 493 498 501 505 614 655 679 681 764 796 954 969

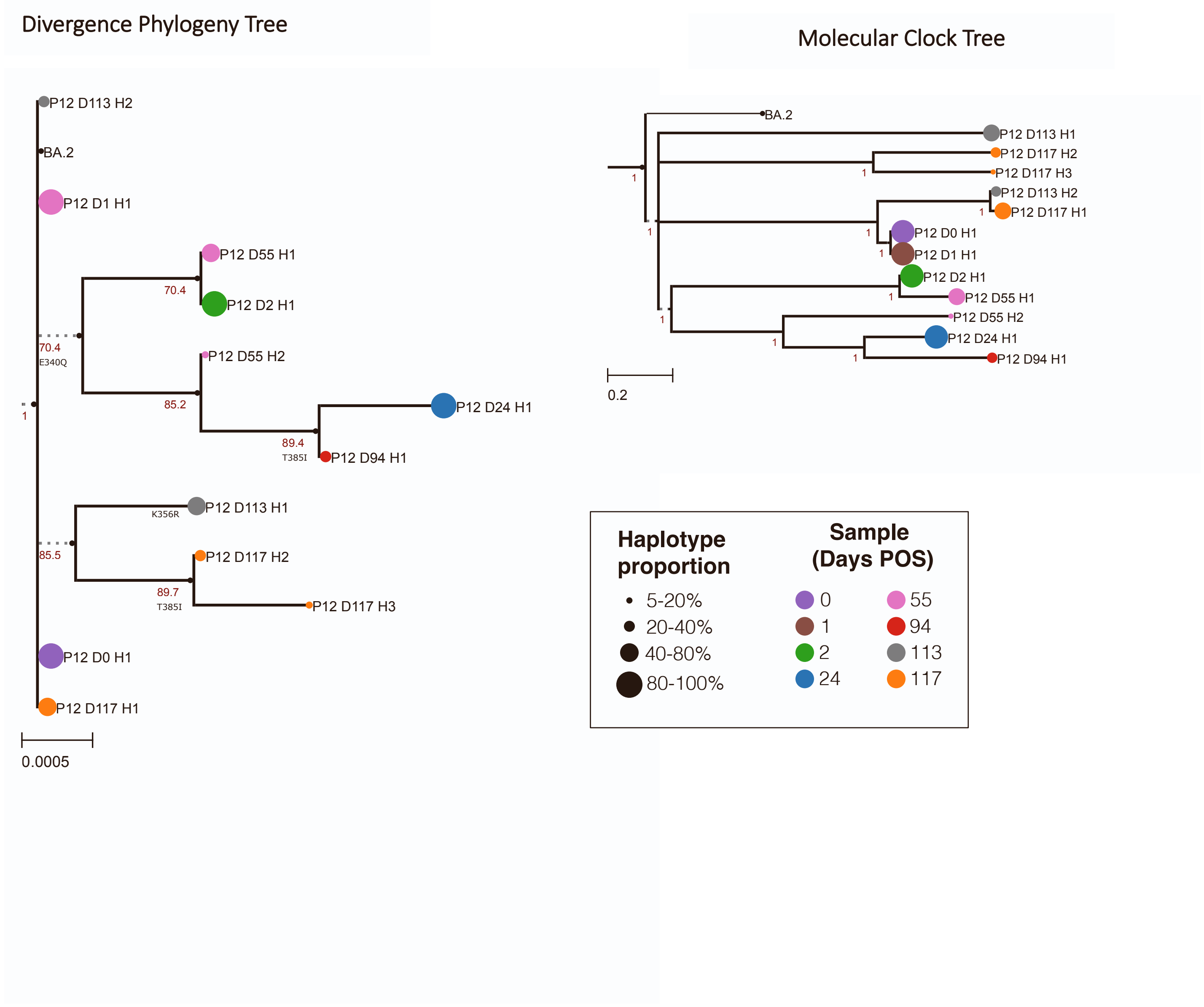

Figure S4M

Patient 13

	19	24	25	26	27	142	213	299	308	339	371	373	375	376	385	405	408	417	440	477	478	484	493	498	501	505	614	655	679	681	764	796	944	954	969	1262
	T	L	P	P	A	G	V	T	V	G	S	S	S	T	T	D	R	K	N	S	T	E	Q	Q	N	Y	D	H	N	P	N	D	A	Q	N	E
BA.2	I	-	-	-	S	D	G			D	F	P	F	A		N	S	N	K	N	K	A	R	R	Y	H	G	Y	K	H	K	Y		H	K	
P13 D237 H1	I	-	-	-	S	D	G	I	I	D	F	P	F	A	I		S	N	K	N	K	A	R	R	Y	H	G	Y	K	H	K	Y	V	H	K	G
	19	24	25	26	27	142	213	299	308	339	371	373	375	376	385	405	408	417	440	477	478	484	493	498	501	505	614	655	679	681	764	796	944	954	969	1262

TREES NOT AVAILABLE

Figure S4N

Patient 14

Amino-acid Matrix with NS mutations

	19	24	25	26	27	142	213	339	371	373	375	376	405	408	417	440	477	478	484	493	498	501	505	614	655	679	681	764	796	954	969
	T	L	P	P	A	G	V	G	S	S	S	T	D	R	K	N	S	T	E	Q	Q	N	Y	D	H	N	P	N	D	Q	N
BA.2	I	-	-	-	S	D	G	D	F	P	F	A	N	S	N	K	N	K	A	R	R	Y	H	G	Y	K	H	K	Y	H	K
P14 D34 H1	I	-	-	-	S	D	G	D	F	P	F	A	N	S	N	K	N	K	A	R	R	Y	H	G	Y	K	H	K	Y	H	K
P14 D34 H3	I	-	-	-	S	D	G	D	F	P	F	A	N	S	N	K	N	K	A	R	R	Y	H	G	Y	K	H	K	H	H	K
P14 D39 H1	I	-	-	-	S	D	G	D	F	P	F	A	N	S	N	K	N	K	A	R	R	Y	H	G	Y	K	H	K	Y	H	K

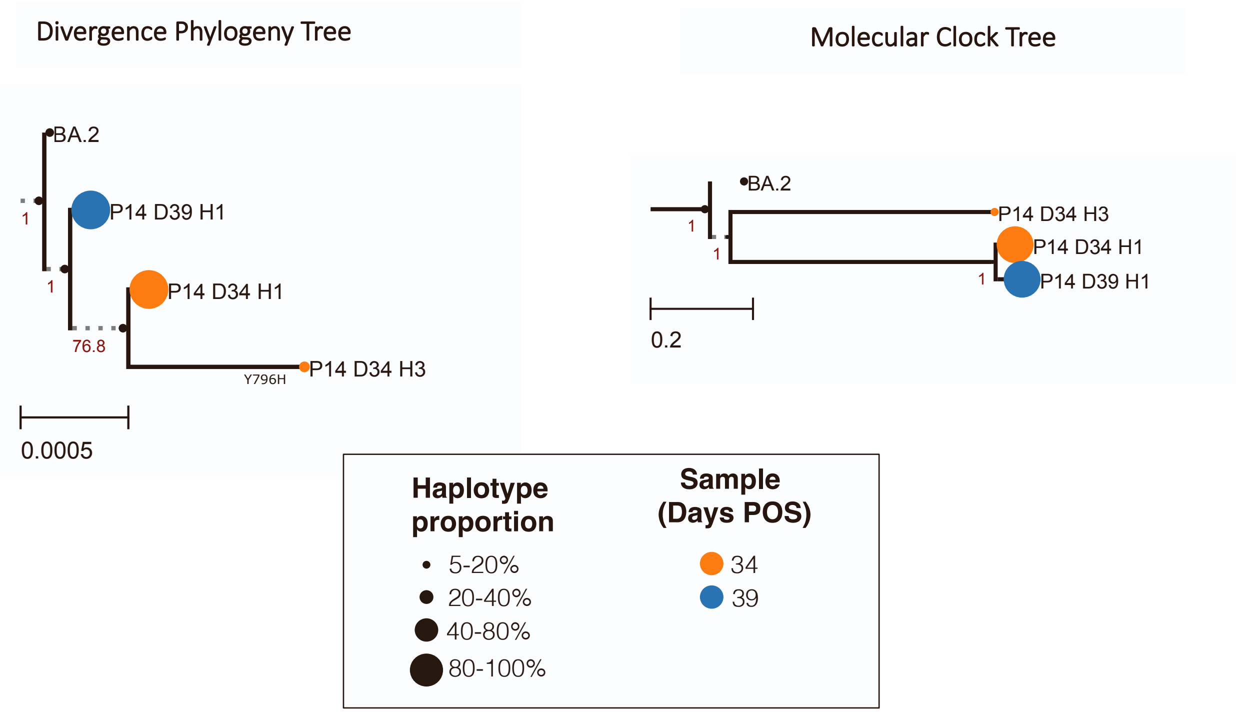

Figure S4O

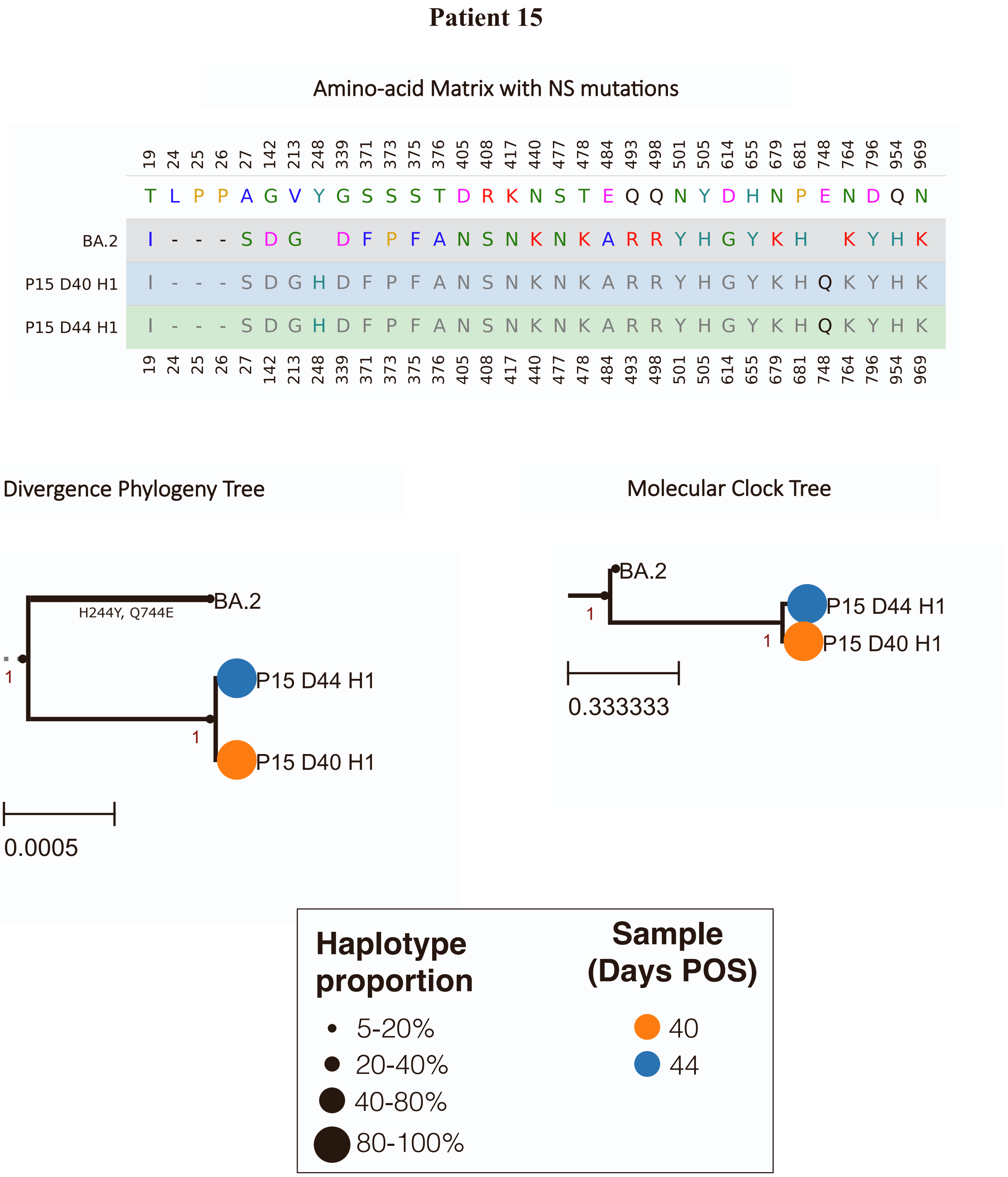
Patient 15
Amino-acid Matrix with NS mutations
19 24 25 26 27 142 213 248 339 371 373 375 376 405 408 417 440 477 478 484 493 498 501 505 614 655 679 681 748 764 796 954 969
T L P P A G V Y G S S S T D R K N S T E Q Q N Y D H N P E N D Q N
BA.2 I - - - S D G D F P F A N S N K N K A R R Y H G Y K H K Y H K
P15 D40 H1 I - - - S D G H D F P F A N S N K N K A R R Y H G Y K H Q K Y H K
P15 D44 H1 I - - - S D G H D F P F A N S N K N K A R R Y H G Y K H Q K Y H K
Divergence Phylogeny Tree
BA.2
H244Y, Q744E
1
P15 D44 H1
1
P15 D40 H1
0.0005
Molecular Clock Tree
BA.2
1
P15 D44 H1
1
P15 D40 H1
0.333333
Haplotype proportion
5-20%
20-40%
40-80%
80-100%
Sample (Days POS)
40
44

Figure S4P

Patient 16

Amino-acid Matrix with NS mutations

	19	24	25	26	27	109	142	213	339	371	373	375	376	405	408	417	440	477	478	484	493	498	501	505	515	614	655	679	681	764	796	954	969	1264
	T	L	P	P	A	T	G	V	G	S	S	S	T	D	R	K	N	S	T	E	Q	Q	N	Y	F	D	H	N	P	N	D	Q	N	V
BA.2	I	-	-	-	S		D	G	D	F	P	F	A	N	S	N	K	N	K	A	R	R	Y	H		G	Y	K	H	K	Y	H	K	
P16 D0 H1	I	-	-	-	S		D	G	D	F	P	F	A	N	S	N	K	N	K	A	R	R	Y	H		G	Y	K	H	K	Y	H	K	
P16 D0 H2	I	-	-	-	S		D	G	D	F	P	F	A	N	S	N	K	N	K	A	R	R	Y	H	S	G	Y	K	H	K	Y	H	K	
P16 D0 H3	I	-	-	-	S		D	G	D	F	P	F	A	N	S	N	K	N	K	A	R	R	Y	H		G	Y	K	H	K	Y	H	K	A
P16 D0 H4	I	-	-	-	S		D	G	D	F	P	F	A	N	S	N	K	N	K	A	R	R	Y	H	S	G	Y	K	H	K	Y	H	K	
P16 D5 H1	I	-	-	-	S		D	G	D	F	P	F	A	N	S	N	K	N	K	A	R	R	Y	H		G	Y	K	H	K	Y	H	K	
P16 D6 H1	I	-	-	-	S	A	D	G	D	F	P	F	A	N	S	N	K	N	K	A	R	R	Y	H		G	Y	K	H	K	Y	H	K	

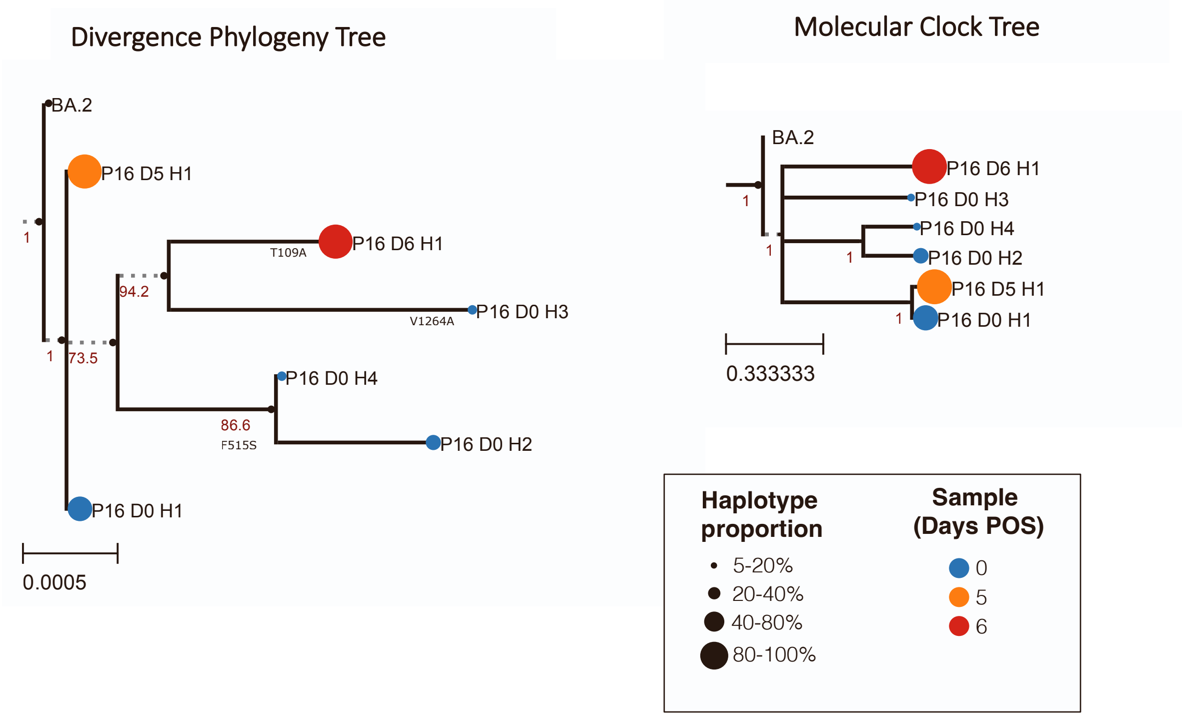

Figure S4Q

Patient 17

Amino-acid Matrix with NS mutations

	19	24	25	26	27	142	213	334	337	339	340	344	346	356	357	370	371	373	375	376	405	408	417	440	477	478	484	493	498	501	504	505	614	655	679	681	764	785	796	954	969	1003
	T	L	P	P	A	G	V	N	P	G	E	A	R	K	R	N	S	S	S	T	D	R	K	N	S	T	E	Q	Q	N	G	Y	D	H	N	P	N	V	D	Q	N	S
BA.2	I	-	-	-	S	D	G			D							F	P	F	A	N	S	N	K	N	K	A	R	R	Y		H	G	Y	K	H	K		Y	H	K	
P17 D80 H1	I	-	-	-	S	D	G			D				R			F	P	F	A	N	S	N	K	N	K	A	R	R	Y		H	G	Y	K	H	K		Y	H	K	
P17 D81 H1	I	-	-	-	S	D	G			D				R			F	P	F	A	N	S	N	K	N	K	A	R	R	Y		H	G	Y	K	H	K		Y	H	K	
P17 D95 H1	I	-	-	-	S	D	G			D			I	R			F	P	F	A	N	S	N	K	N	K	A	R	R	Y		H	G	Y	K	H	K		Y	H	K	
P17 D95 H2	I	-	-	-	S	D	G			D		S		R			F	P	F	A	N	S	N	K	N	K	A	R	R	Y		H	G	Y	K	H	K		Y	H	K	
P17 D95 H3	I	-	-	-	S	D	G			D				R			F	P	F	A	N	S	N	K	N	K	A	R	R	Y		H	G	Y	K	H	K		Y	H	K	
P17 D95 H4	I	-	-	-	S	D	G			D				R			F	P	F	A	N	S	N	K	N	K	A	R	R	Y		H	G	Y	K	H	K		Y	H	K	
P17 D95 H5	I	-	-	-	S	D	G			D		S		R			F	P	F	A	N	S	N	K	N	K	A	R	R	Y		H	G	Y	K	H	K		Y	H	K	
P17 D95 H6	I	-	-	-	S	D	G			D			I	R			F	P	F	A	N	S	N	K	N	K	A	R	R	Y		H	G	Y	K	H	K		Y	H	K	
P17 D104 H1	I	-	-	-	S	D	G		S	D						K	F	P	F	A	N	S	N	K	N	K	A	R	R	Y	D	H	G	Y	K	H	K		Y	H	K	
P17 D104 H2	I	-	-	-	S	D	G			D	D				K		F	P	F	A	N	S	N	K	N	K	A	R	R	Y	D	H	G	Y	K	H	K	G	Y	H	K	
P17 D104 H3	I	-	-	-	S	D	G		S	D						K	F	P	F	A	N	S	N	K	N	K	A	R	R	Y	D	H	G	Y	K	H	K	G	Y	H	K	
P17 D104 H4	I	-	-	-	S	D	G			D	D				K		F	P	F	A	N	S	N	K	N	K	A	R	R	Y	D	H	G	Y	K	H	K		Y	H	K	
P17 D105 H1	I	-	-	-	S	D	G			D			I	R			F	P	F	A	N	S	N	K	N	K	A	R	R	Y		H	G	Y	K	H	K		Y	H	K	
P17 D108 H1	I	-	-	-	S	D	G			D			I	R			F	P	F	A	N	S	N	K	N	K	A	R	R	Y		H	G	Y	K	H	K		Y	H	K	
P17 D108 H2	I	-	-	-	S	D	G	K		D			I	R			F	P	F	A	N	S	N	K	N	K	A	R	R	Y		H	G	Y	K	H	K		Y	H	K	
P17 D108 H3	I	-	-	-	S	D	G			D				R			F	P	F	A	N	S	N	K	N	K	A	R	R	Y		H	G	Y	K	H	K		Y	H	K	
P17 D114 H1	I	-	-	-	S	D	G	K		D			I	R			F	P	F	A	N	S	N	K	N	K	A	R	R	Y		H	G	Y	K	H	K		Y	H	K	
P17 D114 H2	I	-	-	-	S	D	G			D			I	R			F	P	F	A	N	S	N	K	N	K	A	R	R	Y		H	G	Y	K	H	K		Y	H	K	I
P17 D114 H3	I	-	-	-	S	D	G	K		D			I	R			F	P	F	A	N	S	N	K	N	K	A	R	R	Y		H	G	Y	K	H	K		Y	H	K	
P17 D114 H4	I	-	-	-	S	D	G			D	A			R			F	P	F	A	N	S	N	K	N	K	A	R	R	Y		H	G	Y	K	H	K		Y	H	K	

Molecular Clock Tree

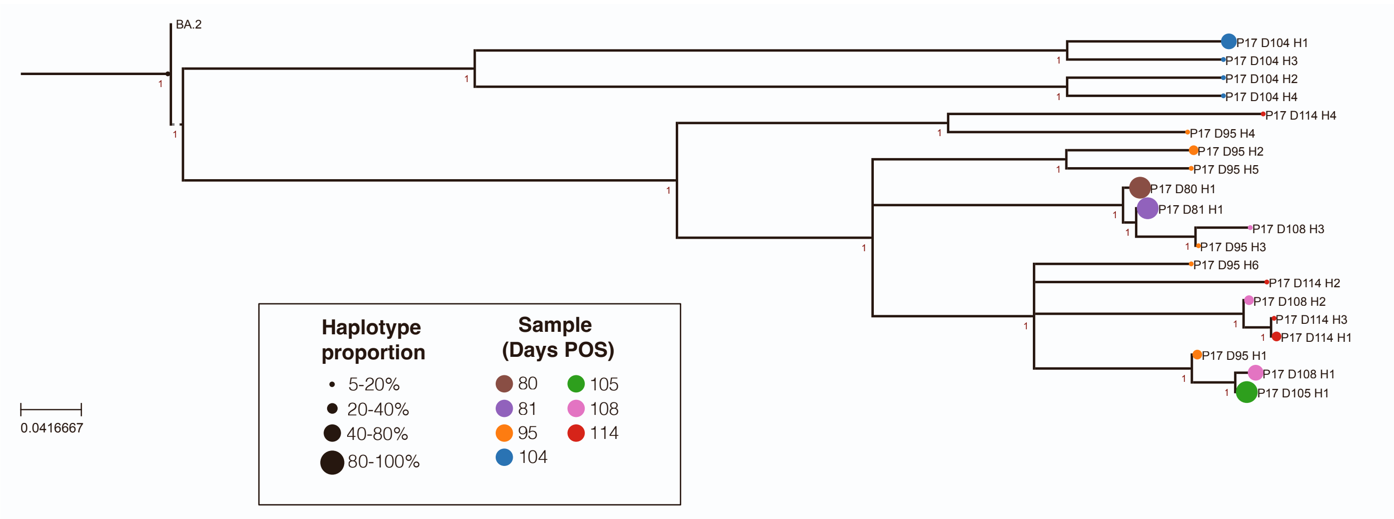

Figure S4Q (cont.)

Patient 17

Divergence Phylogeny Tree

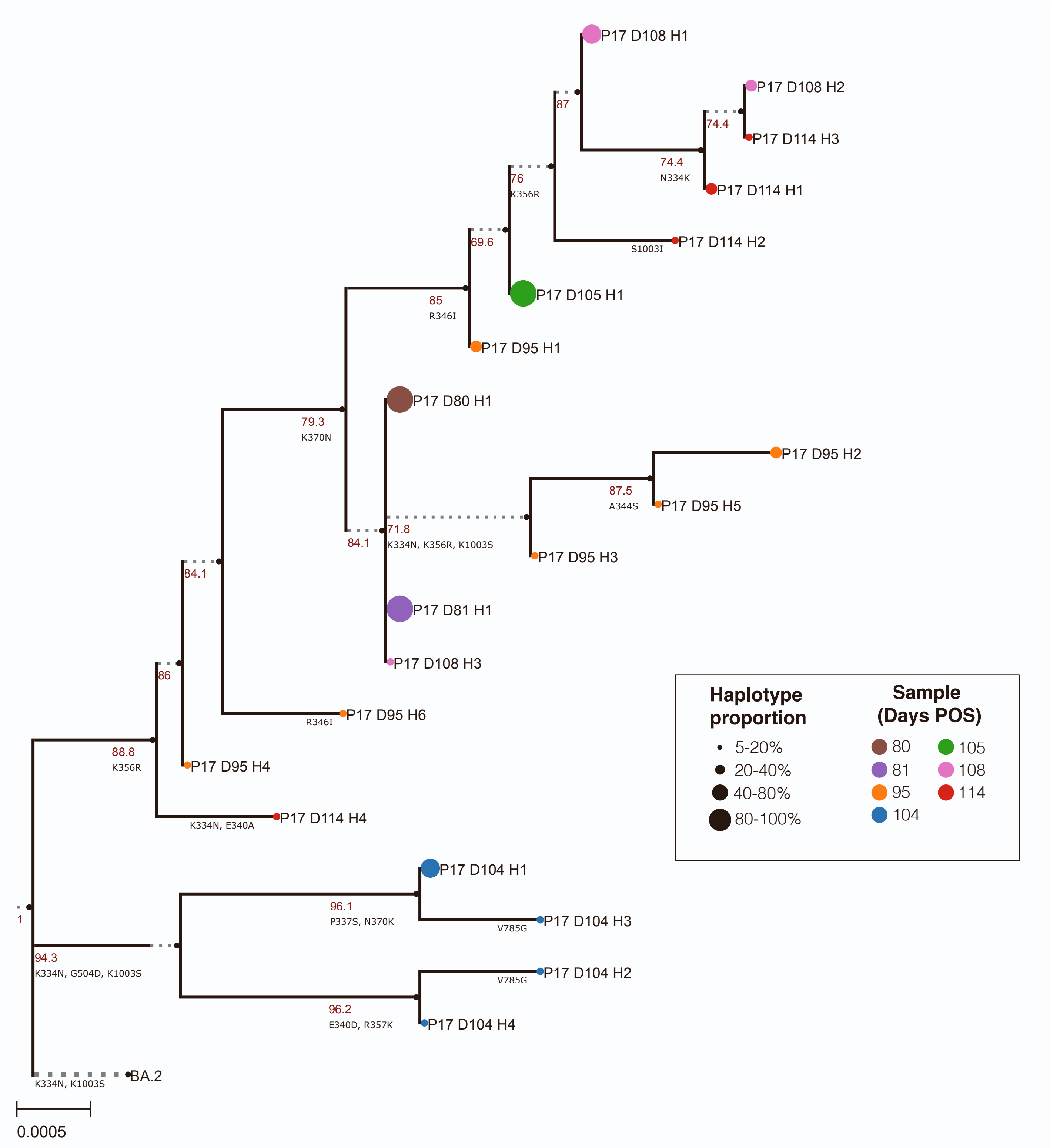

Figure S4R

Patient 18

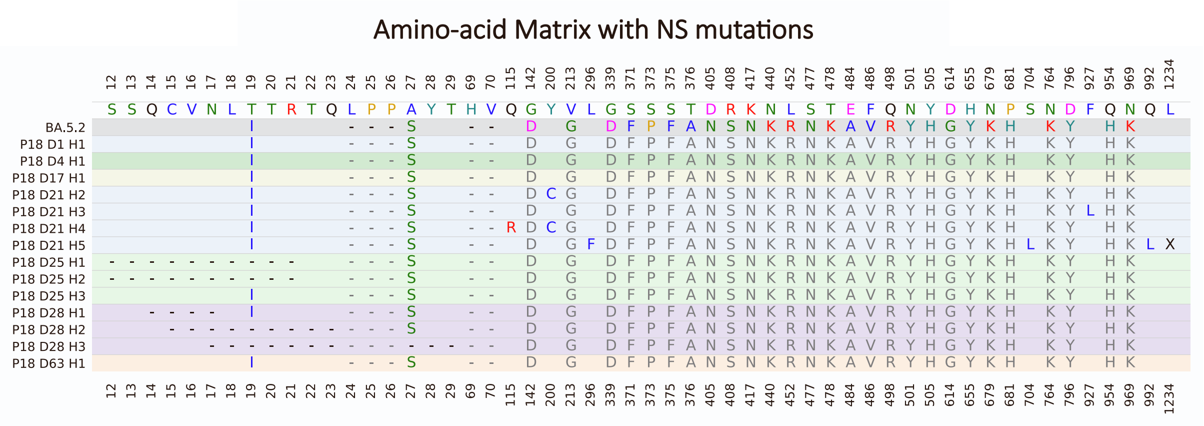

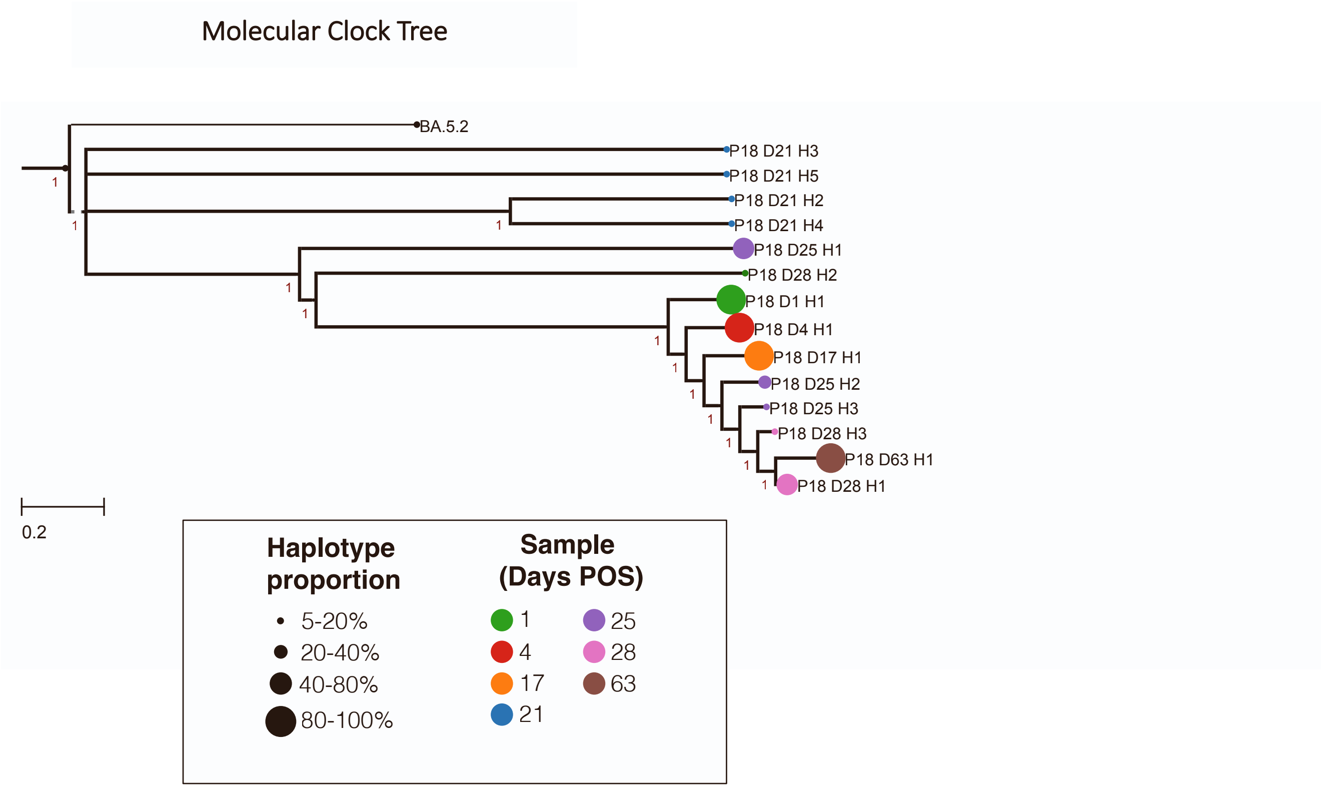

Figure S4R (cont.)

Patient 18

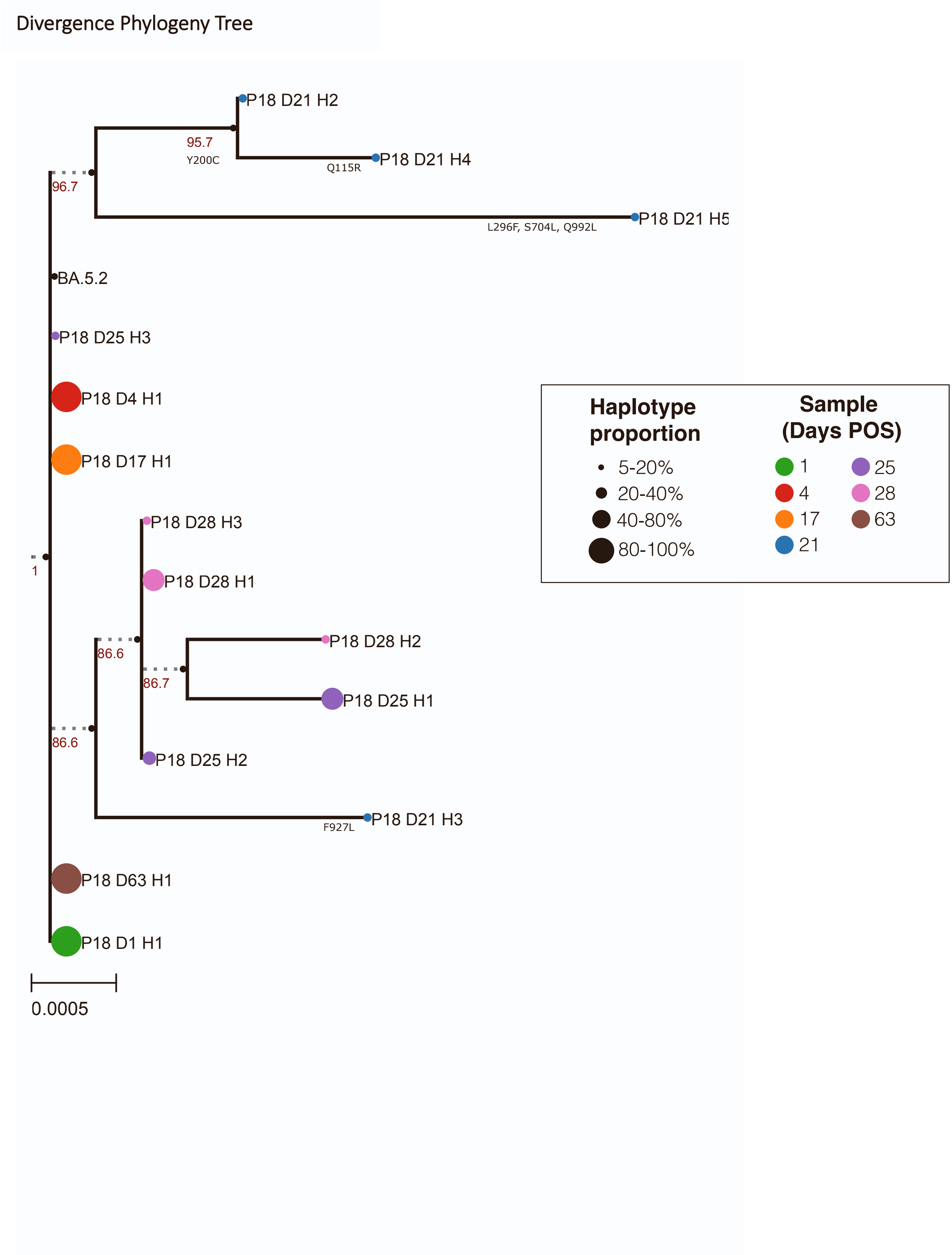

Figure S4S

Patient 19

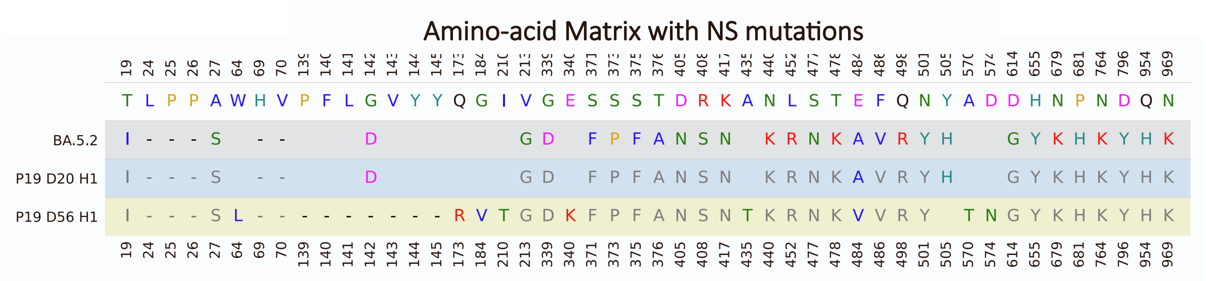

Divergence Phylogeny Tree

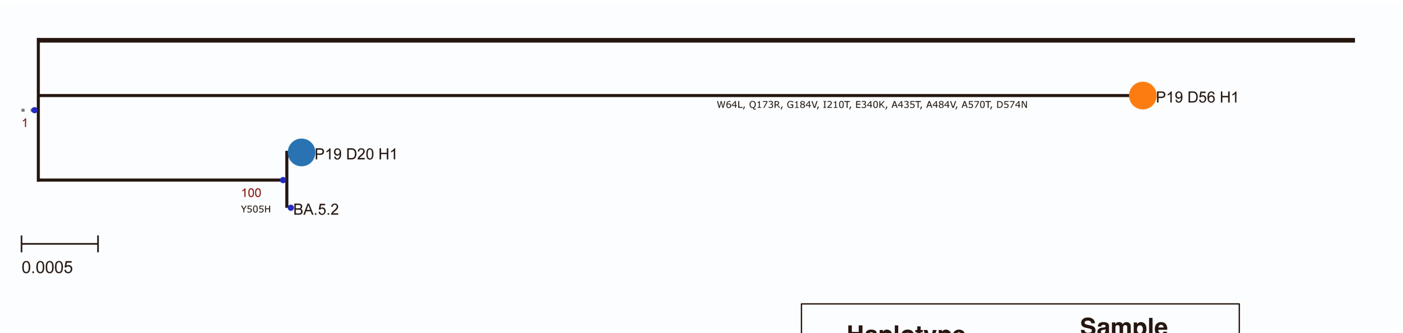

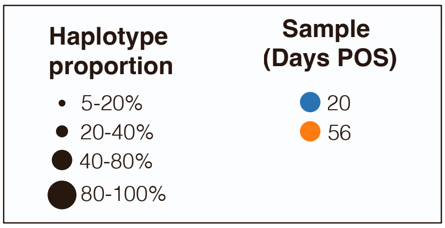

Molecular Clock Tree

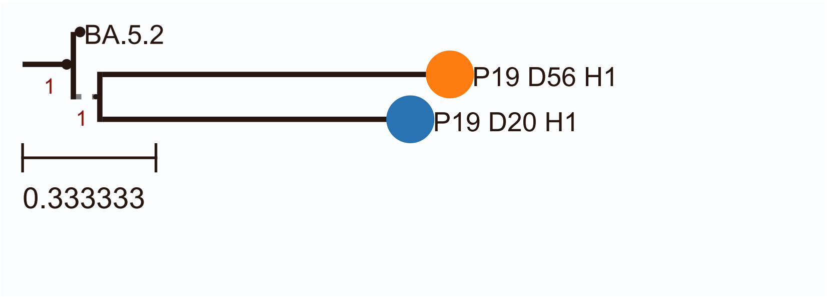

Figure S4T

Patient 20

Amino-acid Matrix with NS mutations

	19	24	25	26	27	69	70	142	144	156	213	323	337	339	340	371	372	373	375	376	405	408	417	440	452	477	478	484	486	498	501	505	614	655	679	681	764	796	824	954	969	1020
	T	L	P	P	A	H	V	G	Y	E	V	T	P	G	E	S	A	S	S	T	D	R	K	N	L	S	T	E	F	Q	N	Y	D	H	N	P	N	D	N	Q	N	A
BA.5.2	I	-	-	-	S	-	-	D			G			D		F		P	F	A	N	S	N	K	R	N	K	A	V	R	Y	H	G	Y	K	H	K	Y		H	K	
P20 D1 H1	I	-	-	-	S	-	-	D			G			D		F		P	F	A	N	S	N	K	R	N	K	A	V	R	Y	H	G	Y	K	H	K	Y		H	K	
P20 D38 H1	I	-	-	-	S	-	-	D			G			D		F		P	F	X	N	S	N	K	R	N	K	A	V	R	Y	H	G	Y	K	H	K	Y		H	K	
P20 D38 H2	I	-	-	-	S	-	-	D			G			D	K	F		P	F	A	N	S	N	K	R	N	K	A	V	R	Y	H	G	Y	K	H	K	Y		H	K	
P20 D38 H3	I	-	-	-	S	-	-	D			G			D	K	F		P	F	A	N	S	N	K	R	N	K	A	V	R	Y	H	G	Y	K	H	K	Y		H	K	
P20 D38 H4	I	-	-	-	S	-	-	D			G			D	K	F		P	F	A	N	S	N	K	R	N	K	A	V	R	Y		G	Y	K	H	K	Y		H	K	
P20 D38 H5	I	-	-	-	S	-	-	D			G			D	G	F		P	F	A	N	S	N	K	R	N	K	A	V	R	Y	H	G	Y	K	H	K	Y		H	K	
P20 D45 H1	I	-	-	-	S	-	-	D			G			D	K	F		P	F	A	N	S	N	K	R	N	K	A	V	R	Y	H	G	Y	K	H	K	Y		H	K	
P20 D45 H2	I	-	-	-	S	-	-	D			G		L	D		F		P			N	S	N	K	R	N	K	A	V	R	Y	H	G	Y	K	H	K	Y		H	K	
P20 D45 H3	I	-	-	-	S	-	-	D			G			D		F		P	F	A	N	S	N	K	R	N	K	A	V	R	Y	H	G	Y	K	H	K	Y		H	K	
P20 D45 H4	I	-	-	-	S	-	-	D		K	G		L	D		-	-	-	A	P	N	S	N	K	R	N	K	A	V	R	Y	H	G	Y	K	H	K	Y		H	K	
P20 D45 H5	I	-	-	-	S	-	-	D			G			D	G	F		P	F	A	N	S	N	K	R	N	K	A	V	R	Y	H	G	Y	K	H	K	Y		H	K	
P20 D51 H1	I	-	-	-	S	-	-	D			G			D	K	F		P	F	A	N	S	N	K	R	N	K	A	V	R	Y	H	G	Y	K	H	K	Y		H	K	
P20 D51 H2	I	-	-	-	S	-	-	D			G			D	K	F		P	F	A	N	S	N	K	R	N	K	A	V	R	Y	H	G	Y	K	H	K	Y		H	K	
P20 D51 H3	I	-	-	-	S	-	-	D	-		G			D	K	F		P	F	A	N	S	N	K	R	N	K	A	V	R	Y	H	G	Y	K	H	K	Y		H	K	
P20 D51 H4	I	-	-	-	S	-	-	D	-		G			D	K	F		P	F	A	N	S	N	K	R	N	K	A	V	R	Y	H	G	Y	K	H	K	Y		H	K	
P20 D59 H1	I	-	-	-	S	-	-	D	-		G			D	K	F		P	F	A	N	S	N	K	R	N	K	A	V	R	Y	H	G	Y	K	H	K	Y	X	H	K	
P20 D59 H2	I	-	-	-	S	-	-	D			G		L	D		F		P			N	S	N	K	R	N	K	A	V	R	Y	H	G	Y	K	H	K	Y		H	K	
P20 D59 H3	I	-	-	-	S	-	-	D	-		G	A		D	K	F		P	F	A	N	S	N	K	R	N	K	A	V	R	Y	H	G	Y	K	H	K	Y		H	K	V
P20 D59 H4	I	-	-	-	S	-	-	D	-		G	A		D	K	F		P	F	A	N	S	N	K	R	N	K	A	V	R	Y	H	G	Y	K	H	K	Y		H	K	

Molecular Clock Tree

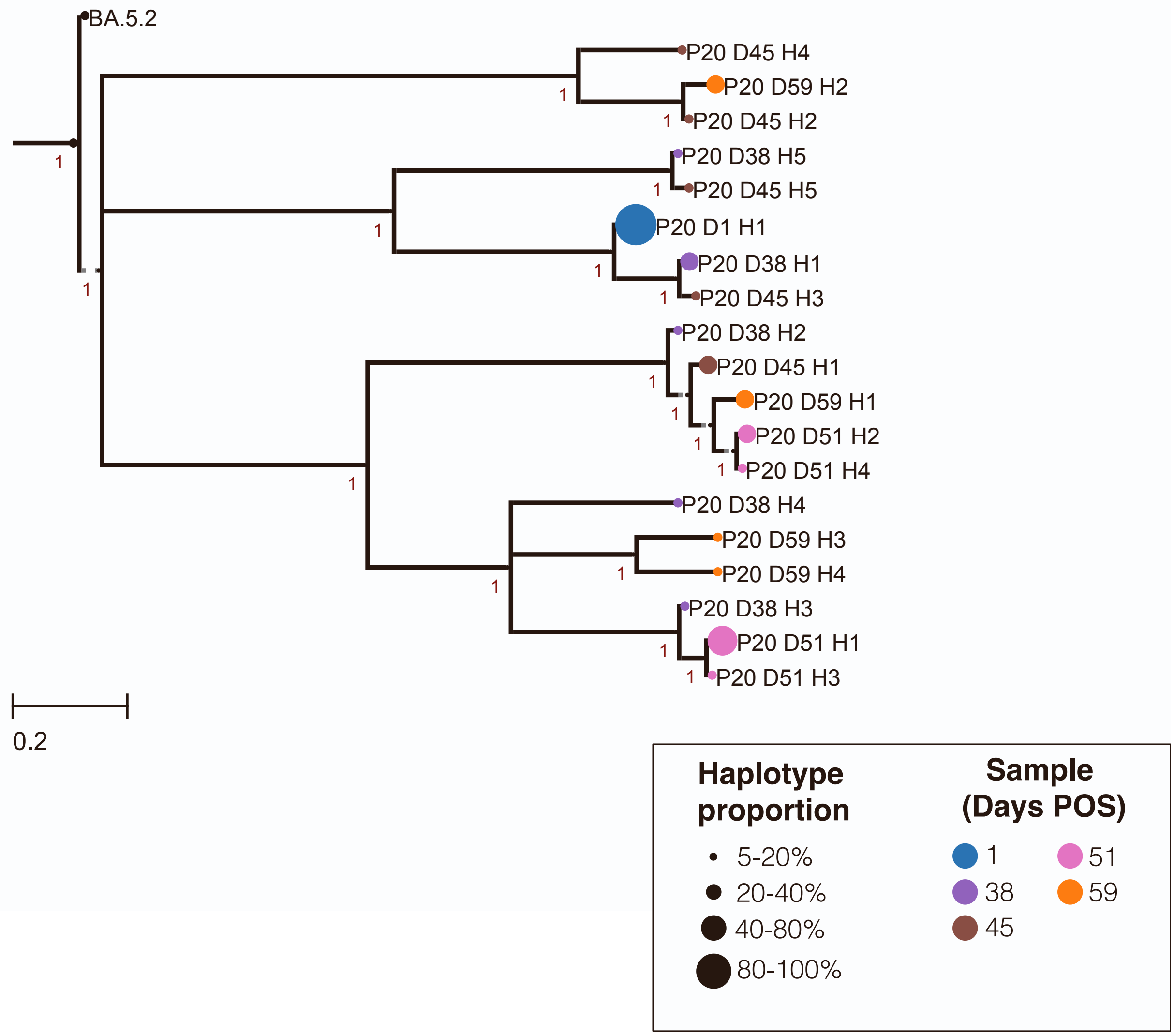

Fugure S4T (cont.)

Patient 20

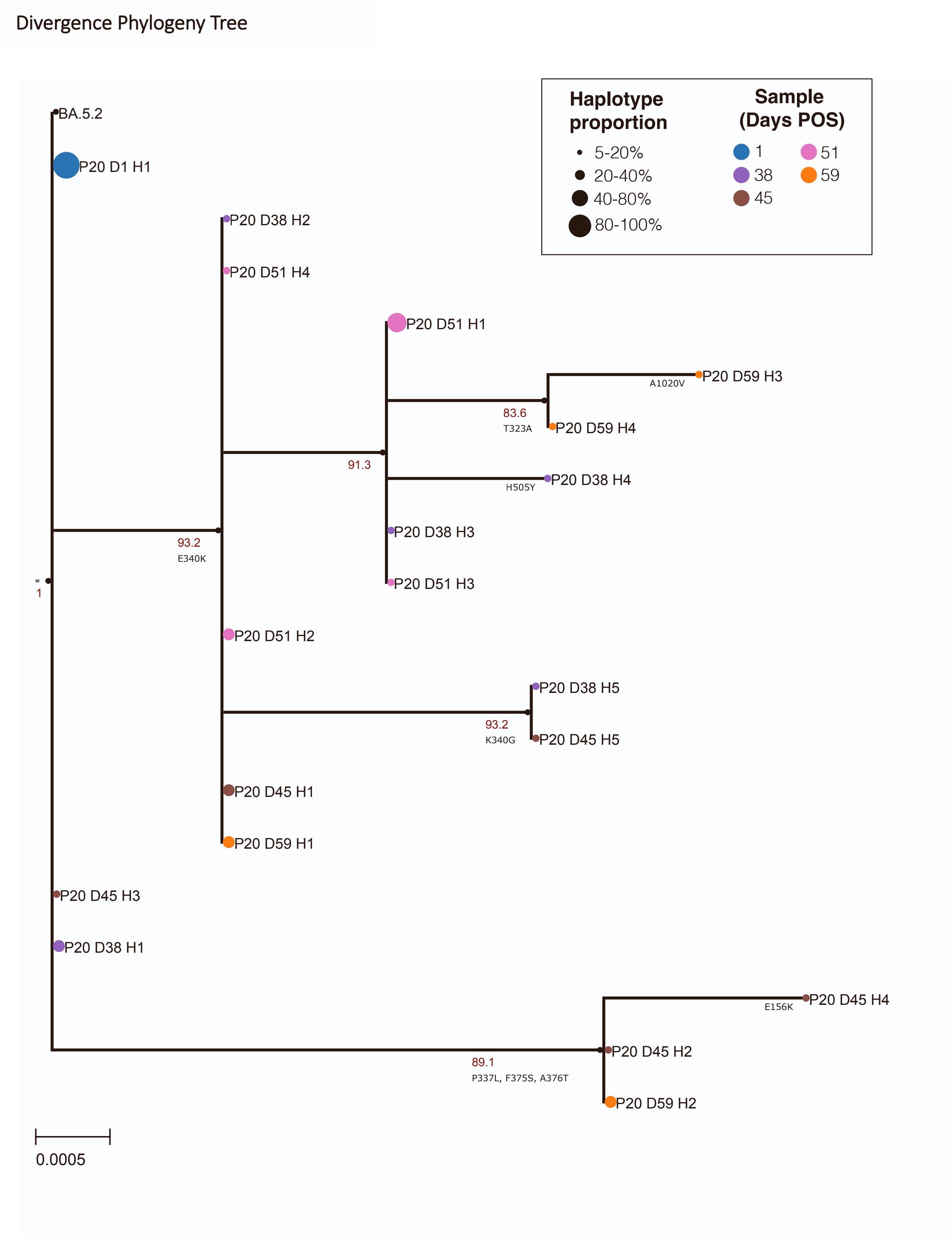

Figure S4U

Patient 21

Amino-acid Matrix with NS mutations

	19	24	25	26	27	68	69	70	105	142	213	330	339	346	371	373	375	376	405	408	417	440	444	452	460	477	478	484	486	498	501	505	614	655	679	681	752	764	796	954	969	1072	1083	1103
	T	L	P	P	A	I	H	V	I	G	V	P	G	R	S	S	S	T	D	R	K	N	K	L	N	S	T	E	F	Q	N	Y	D	H	N	P	L	N	D	Q	N	E	H	F
BQ.1.1	I	-	-	-	S		-	-		D	G		D	T	F	P	F	A	N	S	N	K	T	R	K	N	K	A	V	R	Y	H	G	Y	K	H		K	Y	H	K			
P21 D17 H2	I	-	-	-	S		-	-		D	G	S	D	T	F	P	F	A	N	S	N	K	T	R	K	N	K	A	V	R	Y	H	G	Y	K	H		K	Y	H	K			
P21 D17 H3	I	-	-	-	S		-	-	X	D	G		D	T	F	P	F	A	N	S	N	K	T	R	K	N	K	A	V	R	Y	H	G	Y	K	H		K	Y	H	K	X		
P21 D23 H1	I	-	-	-	S		-	-		D	G	S	D	T	F	P	F	A	N	S	N	K	T	R	K	N	K	A	V	R	Y	H	G	Y	K	H		K	Y	H	K			C
P21 D26 H1	I	-	-	-	S		-	-		D	G	S	D	T	F	P	F	A	N	S	N	K	T	R	K	N	K	A	V	R	Y	H	G	Y	K	H		K	Y	H	K			
P21 D29 H1	I	-	-	-	S		-	-		D	G	S	D	T	F	P	F	A	N	S	N	K	T	R	K	N	K	A	V	R	Y	H	G	Y	K	H	P	K	Y	H	K			
P21 D29 H2	I	-	-	-	S		-	-		D	G	S	D	T	F	P	F	A	N	S	N	K	T	R	K	N	K	A	V	R	Y	H	G	Y	K	H		K	Y	H	K			
P21 D29 H4	I	-	-	-	S	-	-	T		D	G	S	D	T	F	P	F	A	N	S	N	K	T	R	K	N	K	A	V	R	Y	H	G	Y	K	H		K	Y	H	K		L	
P21 D33 H1	I	-	-	-	S		-	-		D	G	S	D	T	F	P	F	A	N	S	N	K	T	R	K	N	K	A	V	R	Y	H	G	Y	K	H		K	Y	H	K			
P21 D33 H2	I	-	-	-	S		-	-		D	G		D	T	F	P	F	A	N	S	N	K	T	R	K	N	K	A	V	R	Y	H	G	Y	K	H		K	Y	H	K			
P21 D42 H1	I	-	-	-	S		-	-		D	G	S	D	T	F	P	F	A	N	S	N	K	T	R	K	N	K	A	V	R	Y	H	G	Y	K	H		K	Y	H	K			
P21 D42 H2	I	-	-	-	S		-	-		D	G		D	T	F	P	F	A	N	S	N	K	T	R	K	N	K	A	V	R	Y	H	G	Y	K	H		K	Y	H	K			

19 24 25 26 27 68 69 70 105 142 213 330 339 346 371 373 375 376 405 408 417 440 444 452 460 477 478 484 486 498 501 505 614 655 679 681 752 764 796 954 969 1072 1083 1103

Molecular Clock Tree

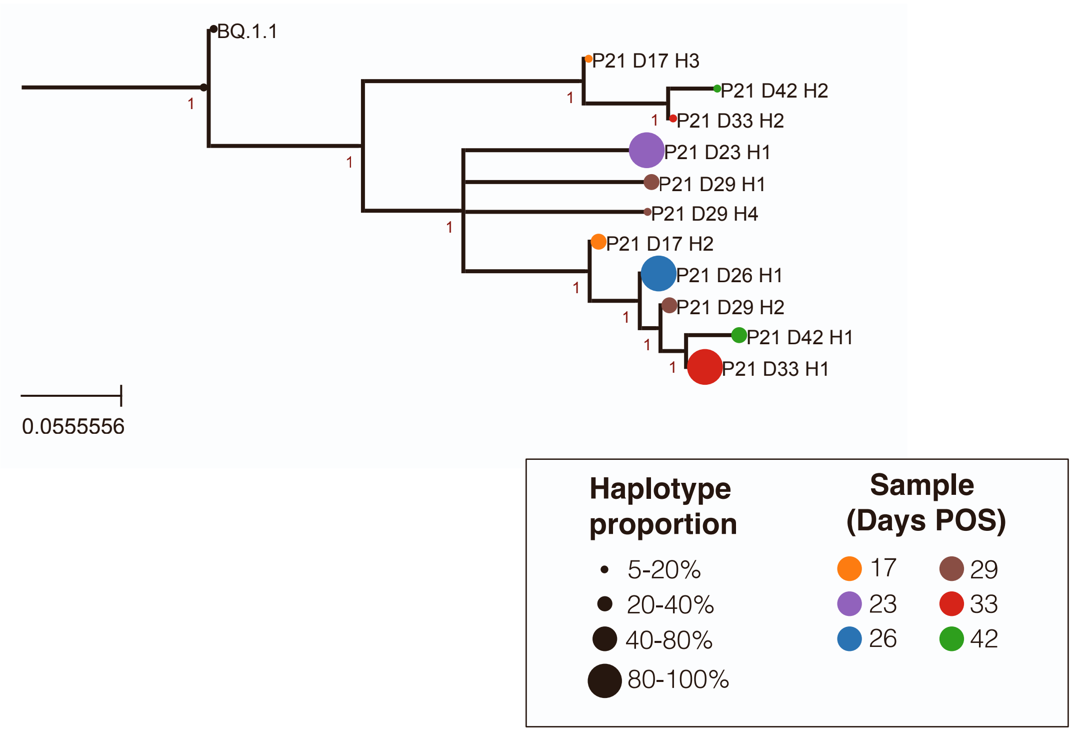

Figure S4U (cont.)

Patient 21

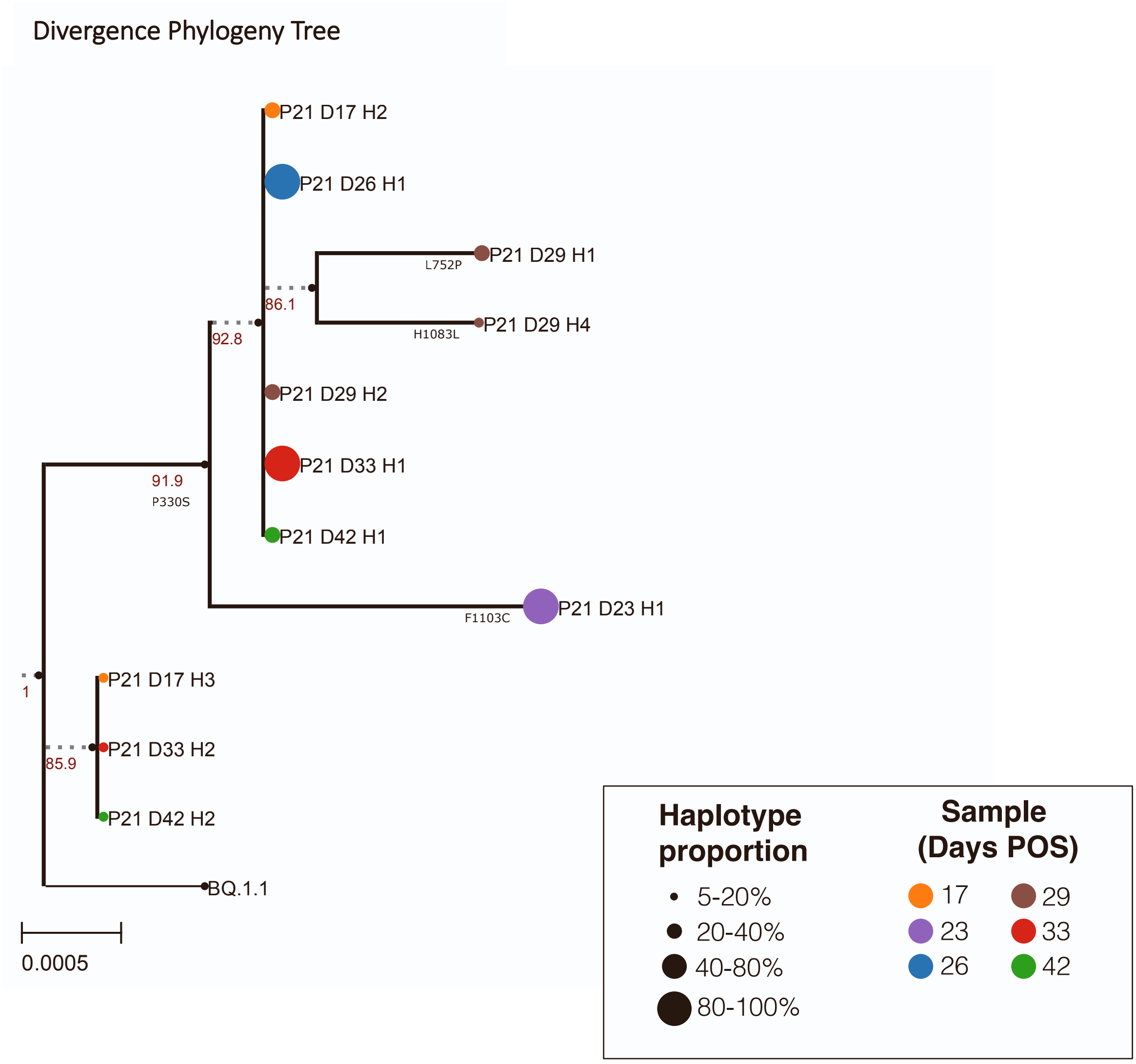

Figure S4V

Patient 22

Amino-acid Matrix with NS mutations

	19	24	25	26	27	83	142	144	146	183	185	186	213	253	339	346	367	368	371	373	375	376
	T	L	P	P	A	V	G	Y	H	Q	N	F	V	D	G	R	V	L	S	S	S	T
GE.1	I	-	-	-	S	A	D	-	Q	E	-	I	E	G	H	T		I	F	P	F	A
P22 D0 H1	I	-	-	-	S	A	D	-	Q	E	-	I	E	G	H	T	F	I	F	P	F	A
P22 D3 H1	I	-	-	-	S	A	D	-	Q	E	-	I	E	G	H	T	F	I	F	P	F	A
P22 D3 H2	I	-	-	-	S	A	D	-	Q	E	-	I	E	G	H	T	F	I	F	P	F	A
P22 D10 H1	I	-	-	-	S	A	D	-	Q	E	-	I	E	G	H	T	F	I	F	P	F	A
P22 D10 H2	I	-	-	-	S	A	D	-	Q	E	-	I	E	G	H	T		I	F	P	F	A
P22 D10 H3	I	-	-	-	S	A	D	-	Q	E	-	I	E	G	H	T		I	F	P	F	A
P22 D10 H4	I	-	-	-	S	A	D	-	Q	E	-	I	E	G	H	T	F	I	F	P	F	A
P22 D21 H1	I	-	-	-	S	A	D	-	Q	E	-	I	E	G	H	T		I	F	P	F	A
P22 D21 H2	I	-	-	-	S	A	D	-	Q	E	-	I	E	G	H	T	F	I	F	P	F	A
	19	24	25	26	27	83	142	144	146	183	185	186	213	253	339	346	367	368	371	373	375	376

	405	408	417	440	445	446	460	477	478	484	486	490	498	501	505	513	521	614	655	679	681	764	796	954	969
	D	R	K	N	V	G	N	S	T	E	F	F	Q	N	Y	L	P	D	H	N	P	N	D	Q	N
GE.1	N	S	N	K	P	S	K	N	R	A	P	S	R	Y	H		S	G	Y	K	H	K	Y	H	K
P22 D0 H1	N	S	N	K	P	S	K	N	R	A	P	S	R	Y	H		S	G	Y	K	H	K	Y	H	K
P22 D3 H1	N	S	N	K	P	S	K	N	R	A	P	S	R	Y	H	F	S	G	Y	K	H	K	Y	H	K
P22 D3 H2	N	S	N	K	P	S	K	N	R	A	P	S	R	Y	H		S	G	Y	K	H	K	Y	H	K
P22 D10 H1	N	S	N	K	P	S	K	N	R	A	P	S	R	Y	H	F	S	G	Y	K	H	K	Y	H	K
P22 D10 H2	N	S	N	K	P	S	K	N	R	A	P	S	R	Y	H		S	G	Y	K	H	K	Y	H	K
P22 D10 H3	N	S	N	K	P	S	K	N	R	A	P	S	R	Y	H	F	S	G	Y	K	H	K	Y	H	K
P22 D10 H4	N	S	N	K	P	S	K	N	R	A	P	S	R	Y	H		S	G	Y	K	H	K	Y	H	K
P22 D21 H1	N	S	N	K	P	S	K	N	R	A	P	S	R	Y	H		S	G	Y	K	H	K	Y	H	K
P22 D21 H2	N	S	N	K	P	S	K	N	R	A	P	S	R	Y	H	F	S	G	Y	K	H	K	Y	H	K
	405	408	417	440	445	446	460	477	478	484	486	490	498	501	505	513	521	614	655	679	681	764	796	954	969

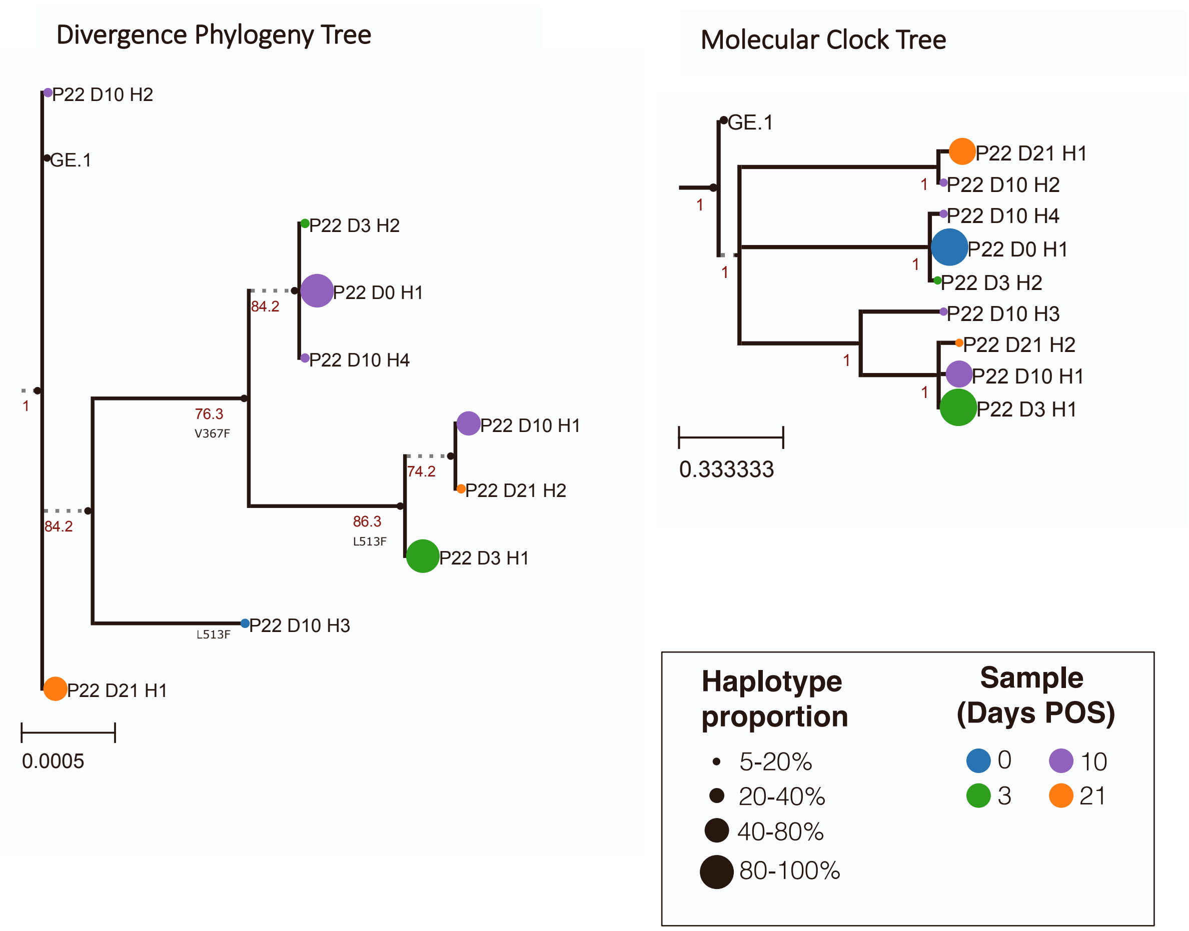

Figure S4W

Patient 23

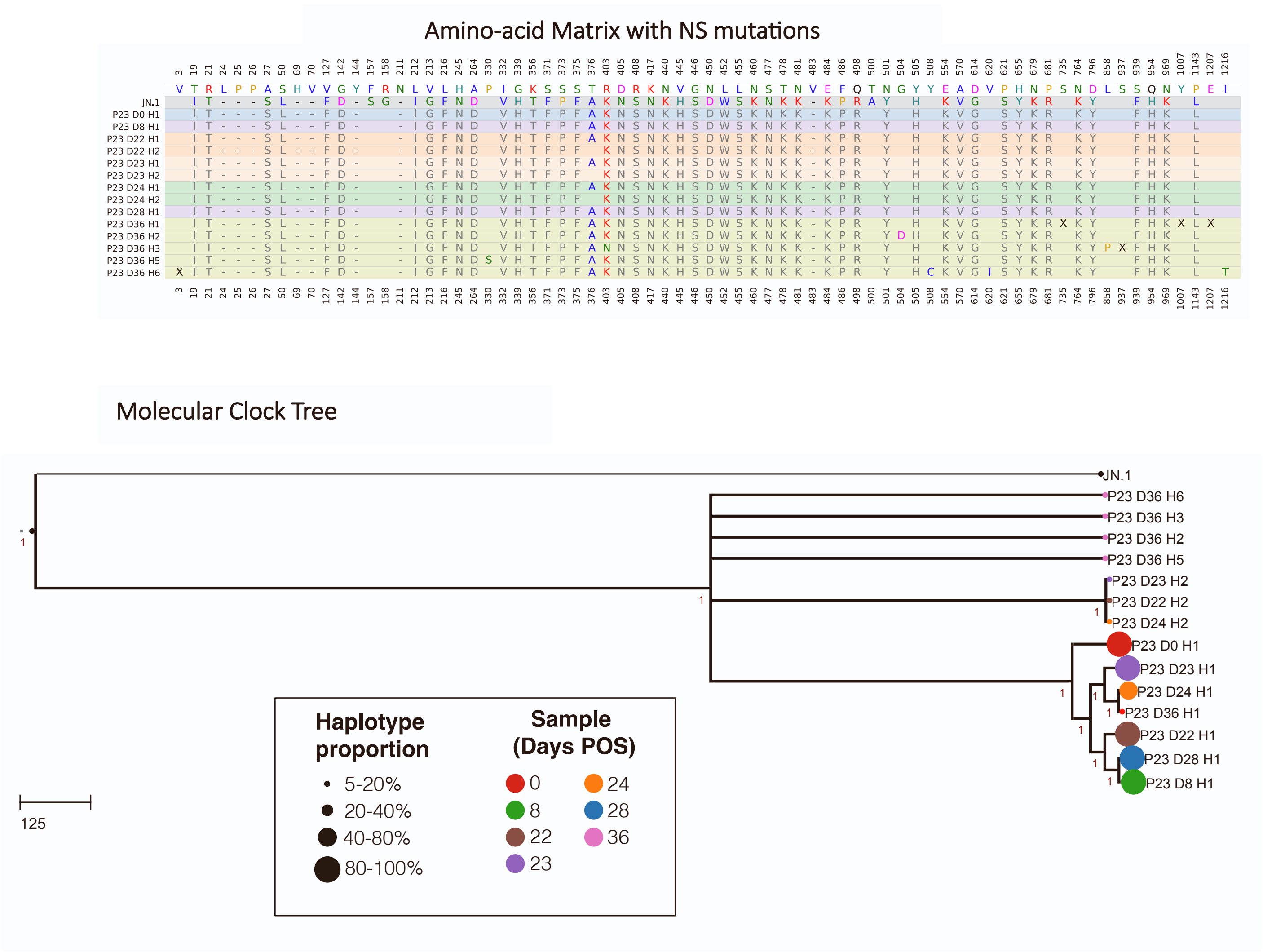

Figure S4W (cont.)

Patient 23

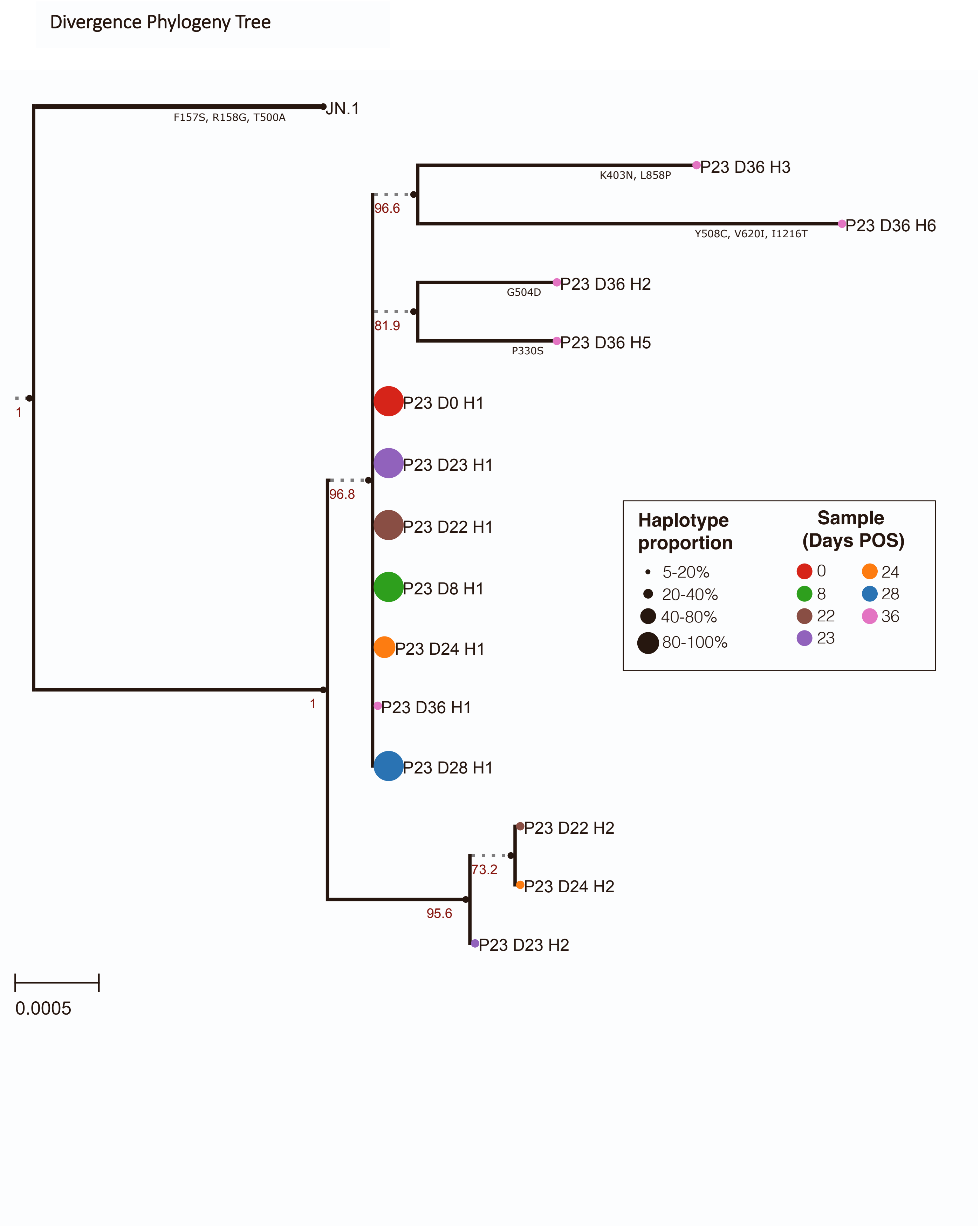

Figure S5

PATIENTS 1-23

Phylogeny Trees based on simple nucleotide divergence model (GTR)

Figure S5. (A-W) Phylogeny Trees based on simple nucleotide divergence model (GTR) for longitudinal samples collected from Patients 1-23. Related to Figure 2 and Figure S4.

Figure S5A

Patient 1

Phylogeny Tree- GTR model

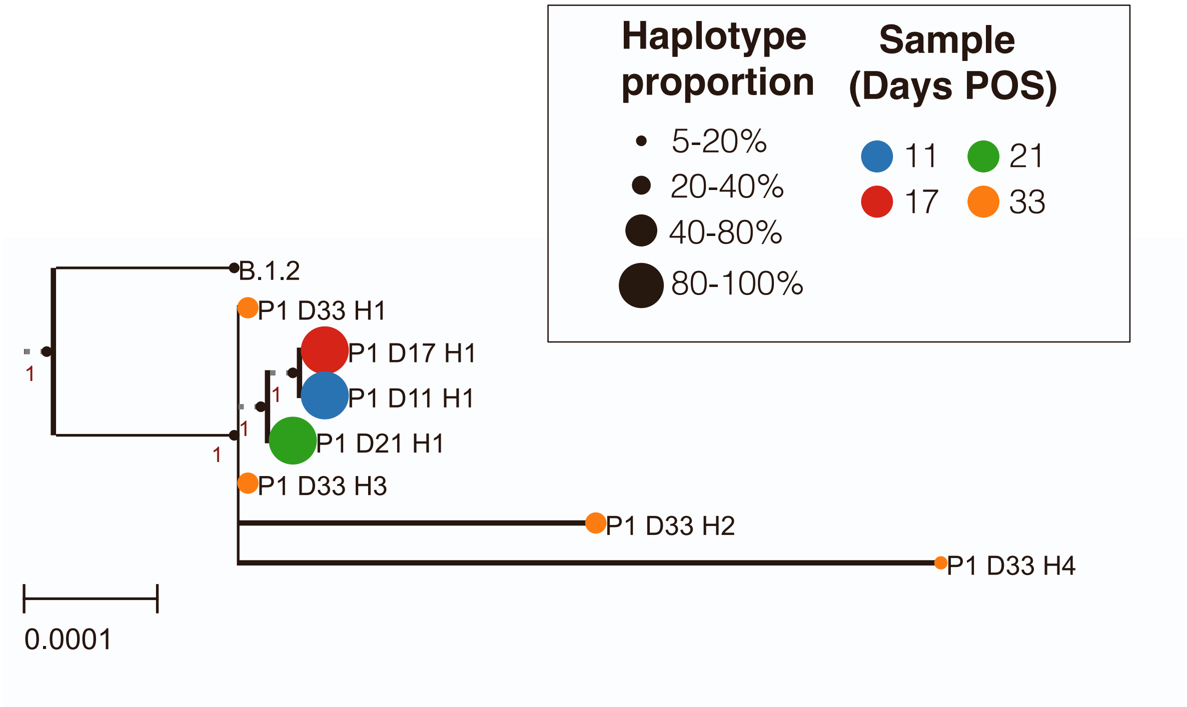

Figure S5B

Patient 2

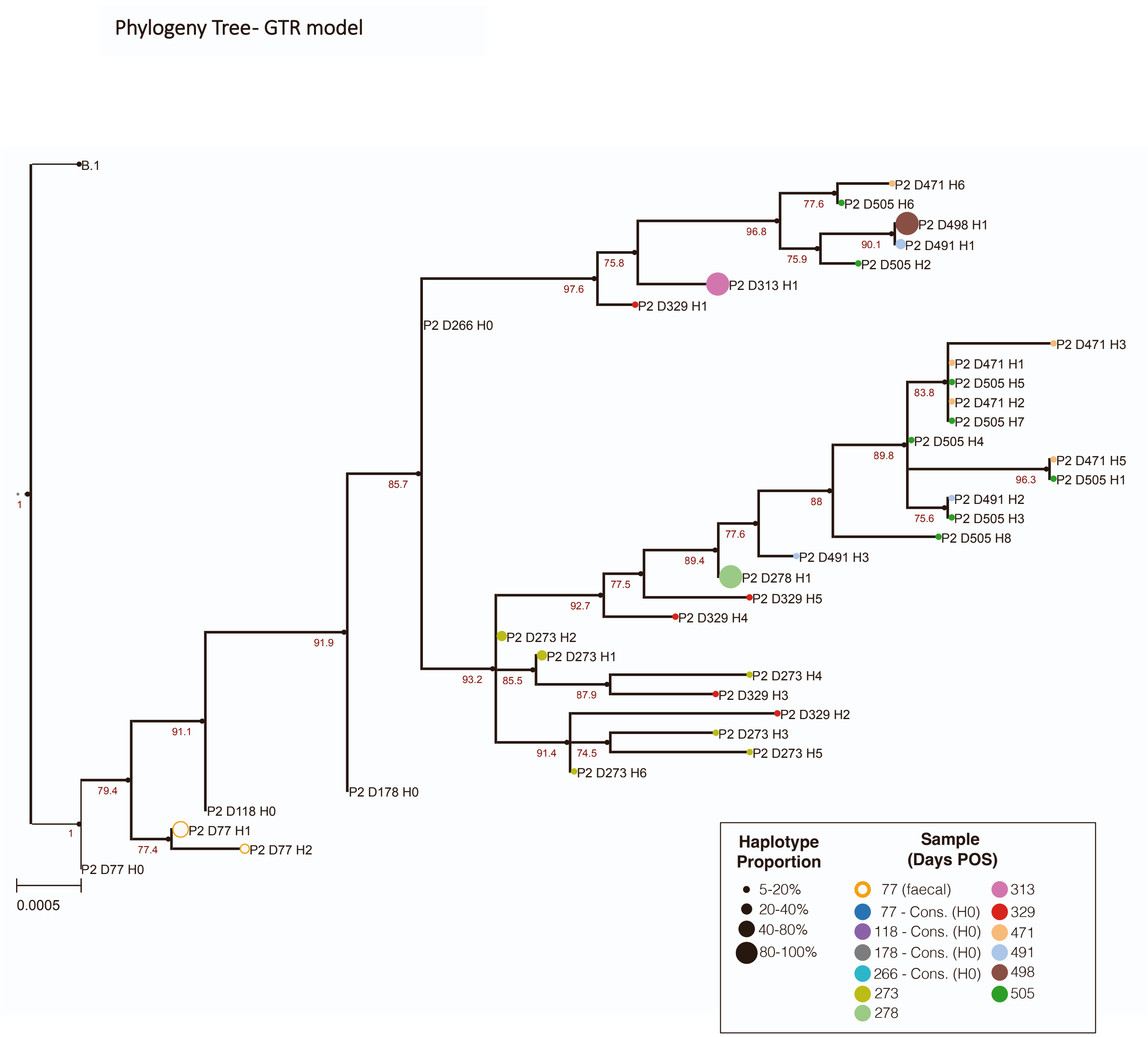

Figure S5C

Patient 3

Phylogeny Tree- GTR model

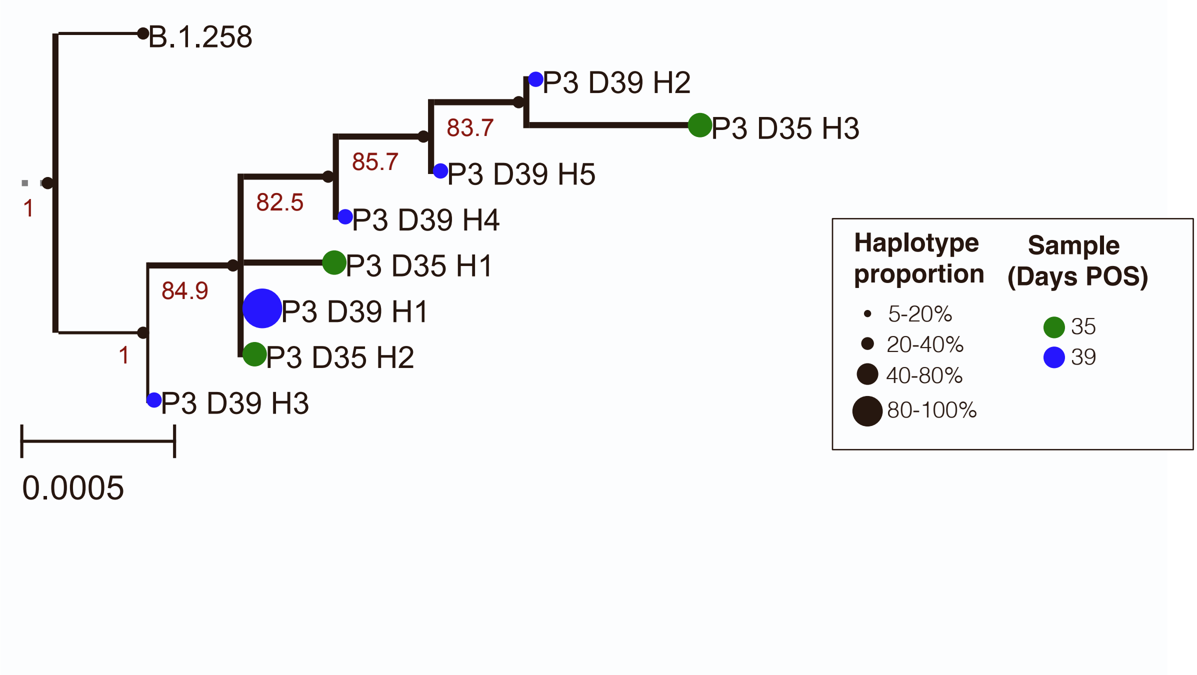

Figure S5D

Patient 4 - 1st infection

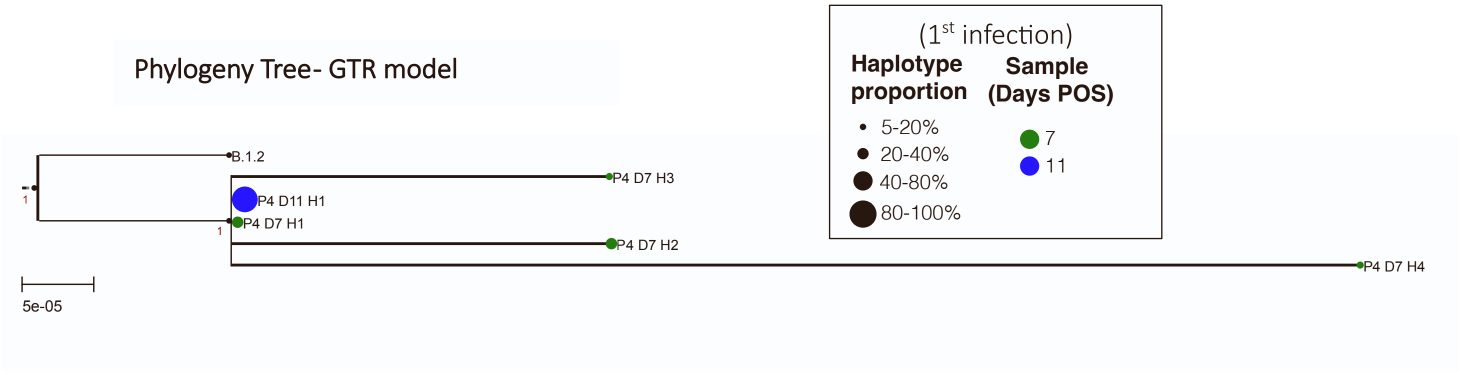

Patient 4 – 2nd infection

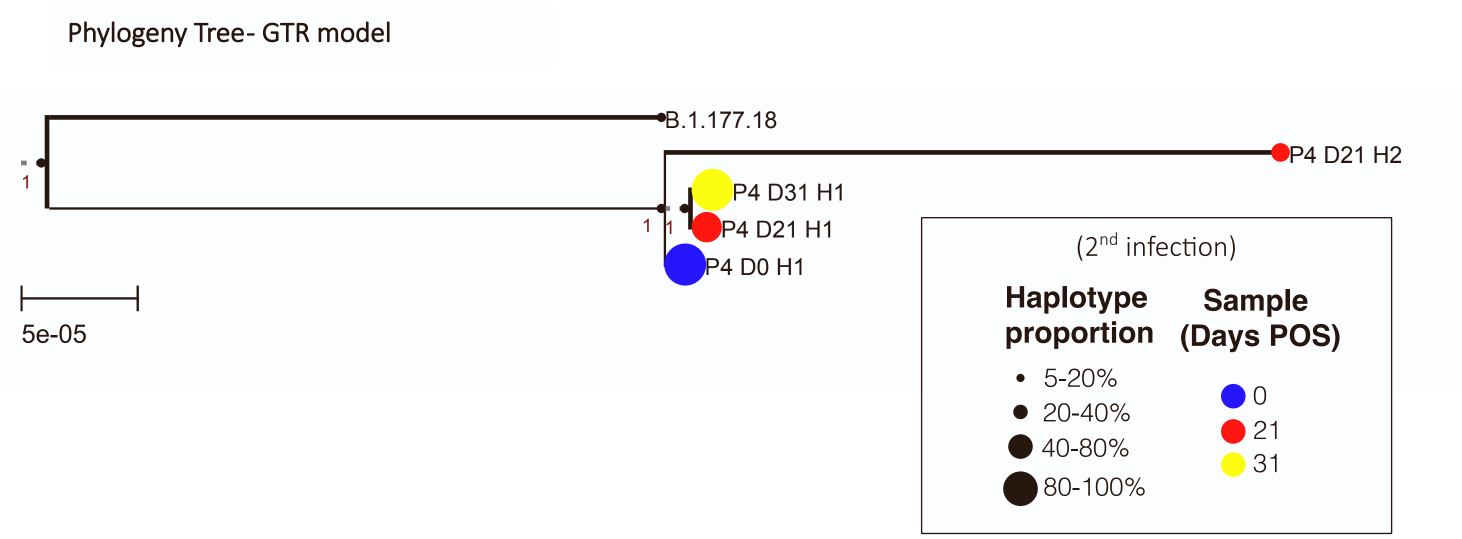

Figure S5E

Patient 5

Phylogeny Tree- GTR model

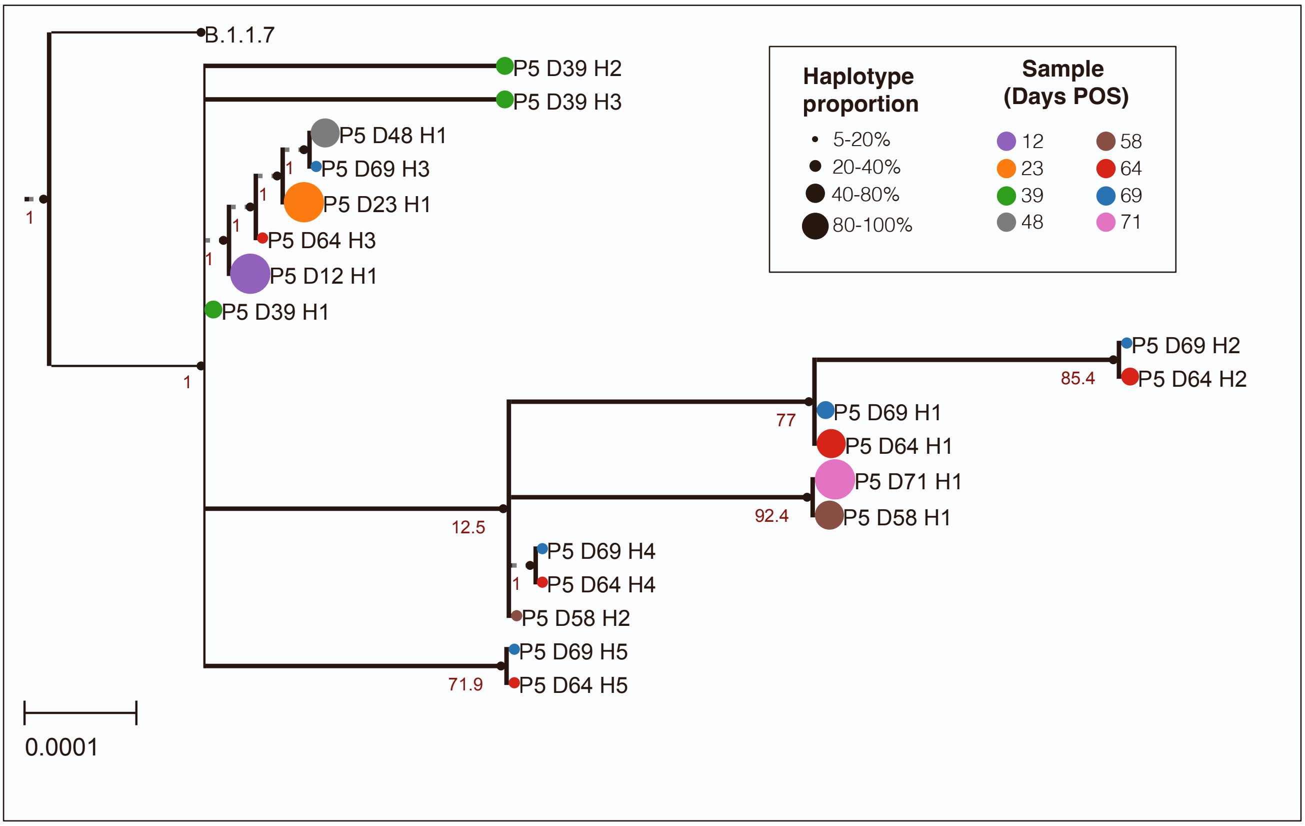

Figure S5F

Patient 6

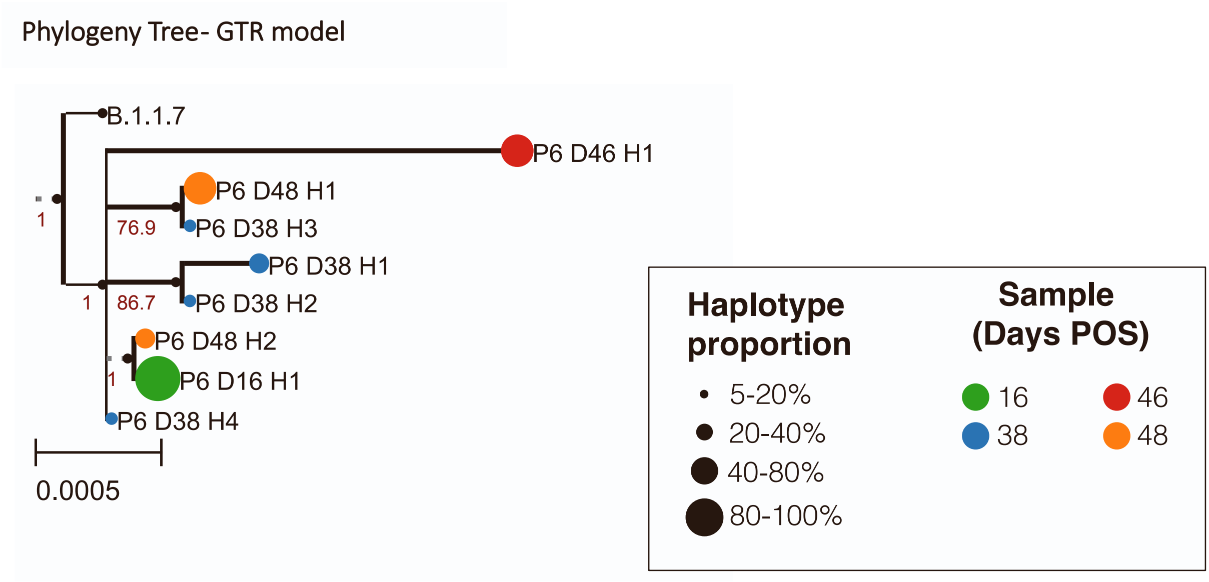

Figure S5G

Patient 7

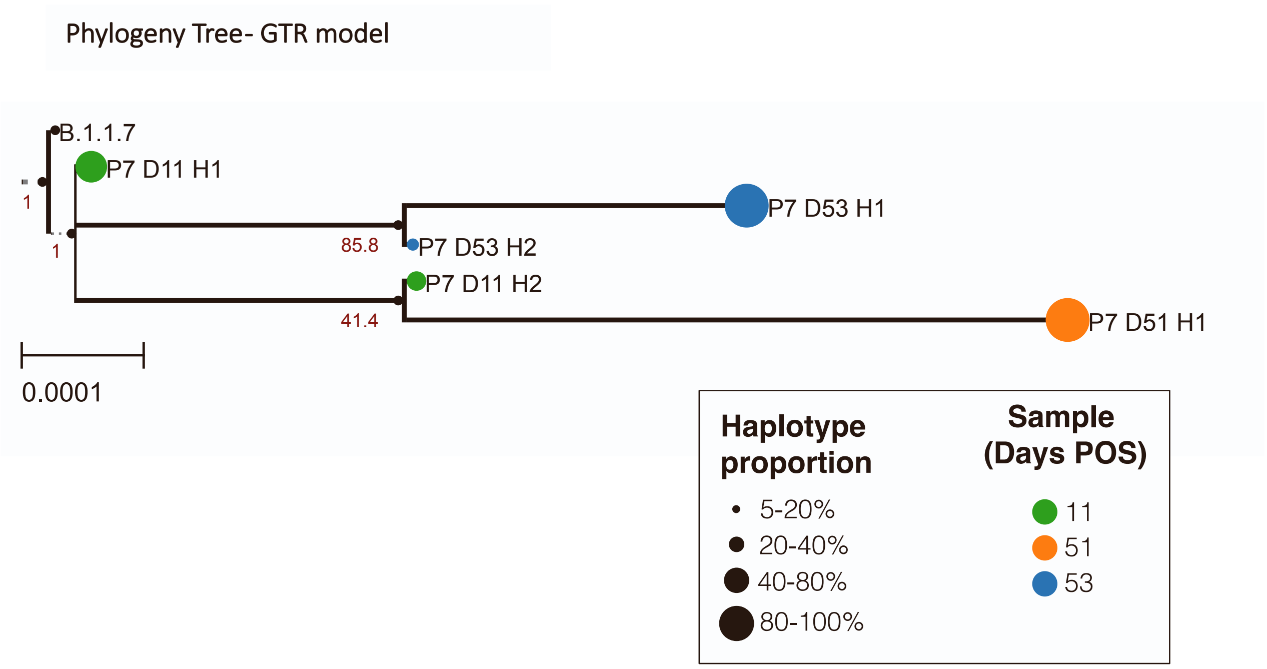

Figure S5H

Patient 8

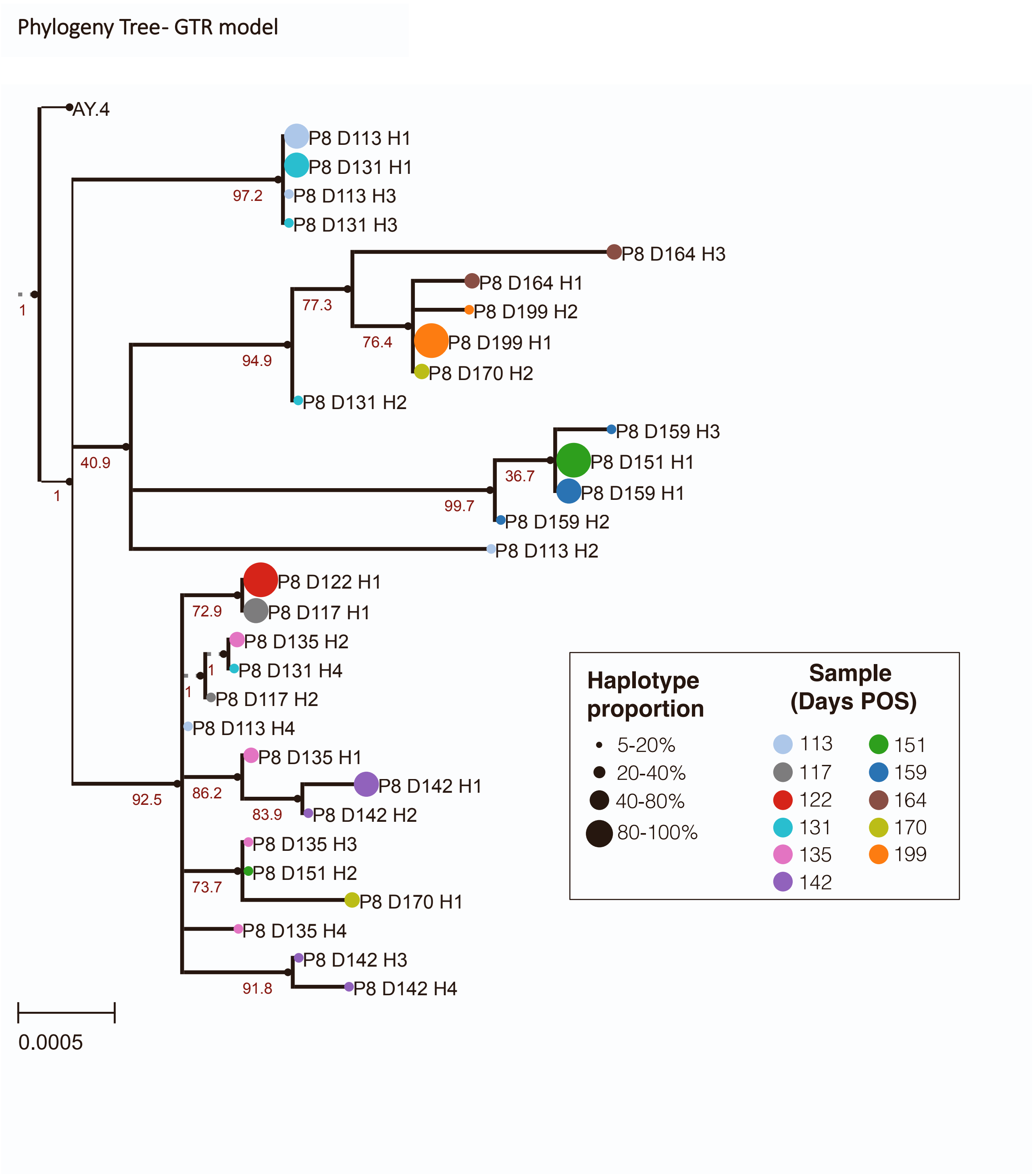

Figure S5I

Patient 9

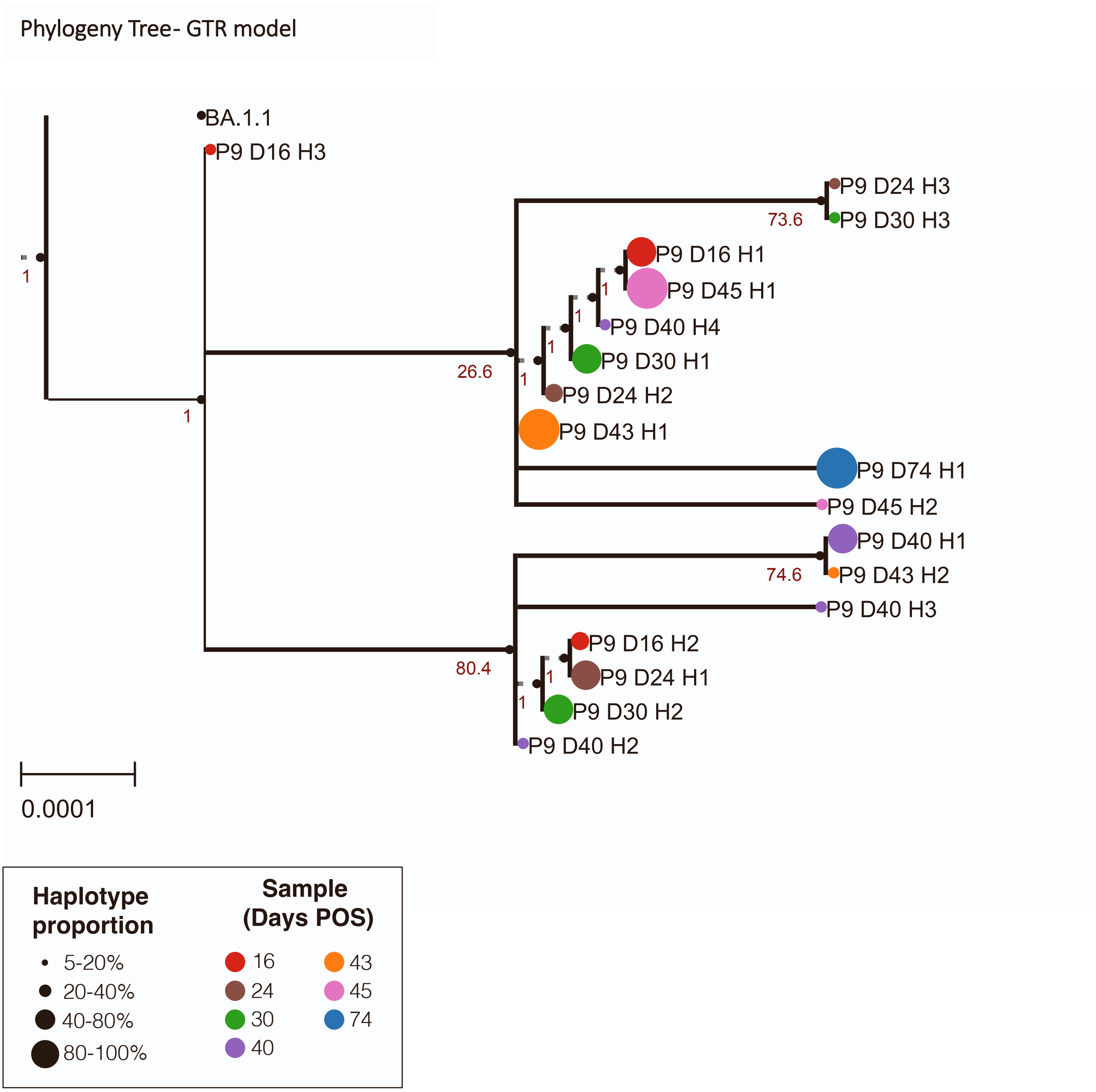

Figure S5J

Patient 10

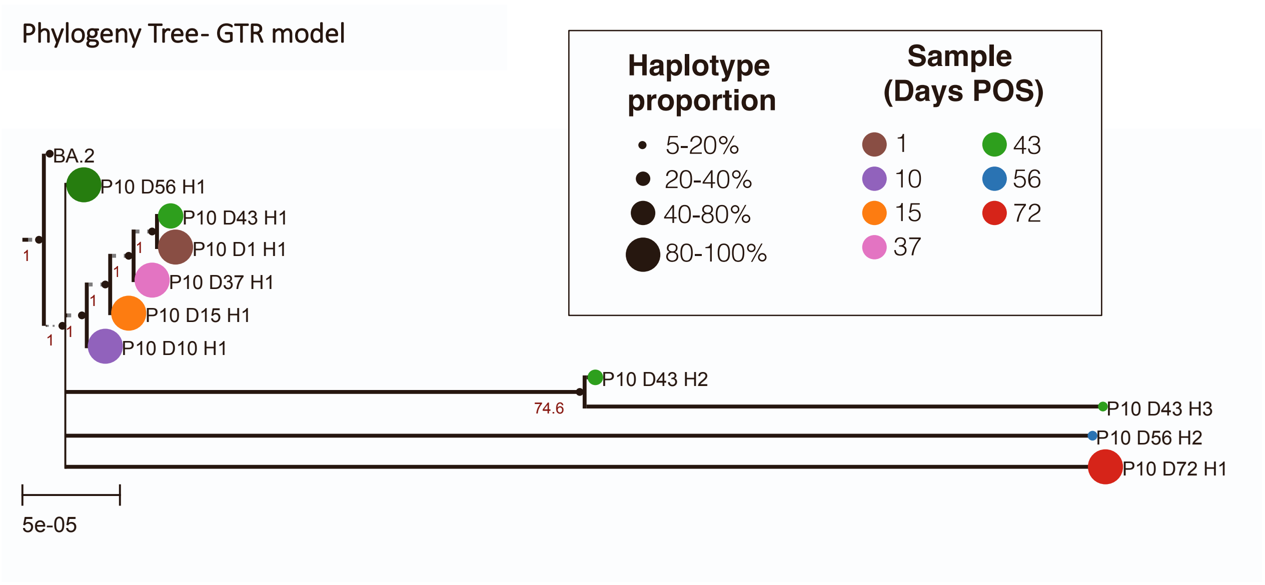

Figure S5K

Patient 11

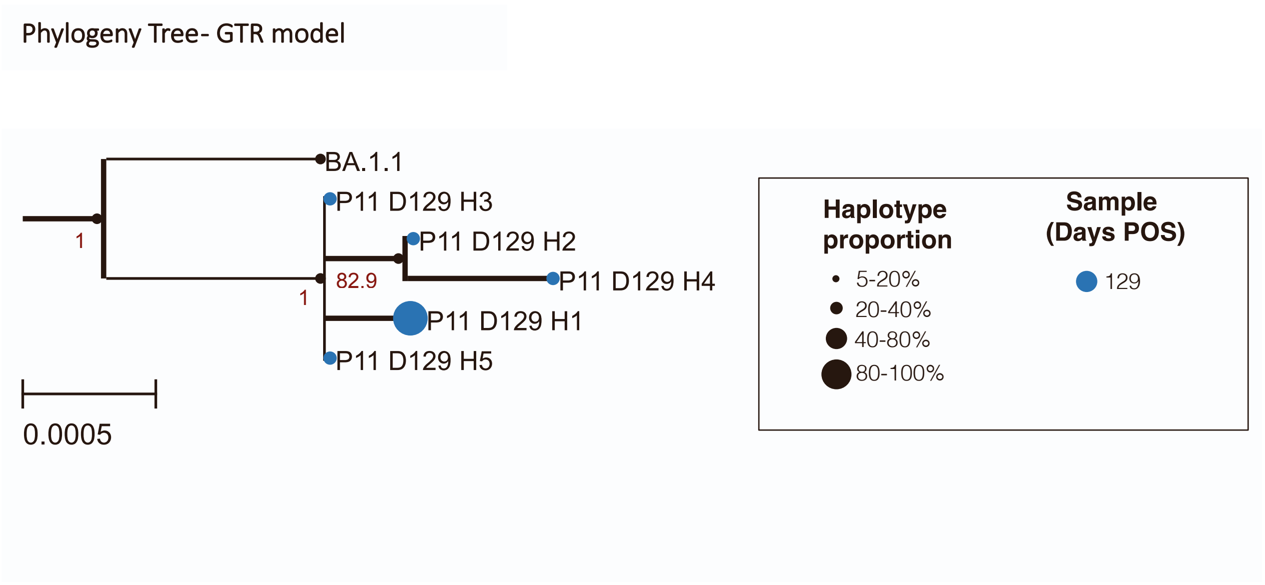

Figure S5L

Patient 12

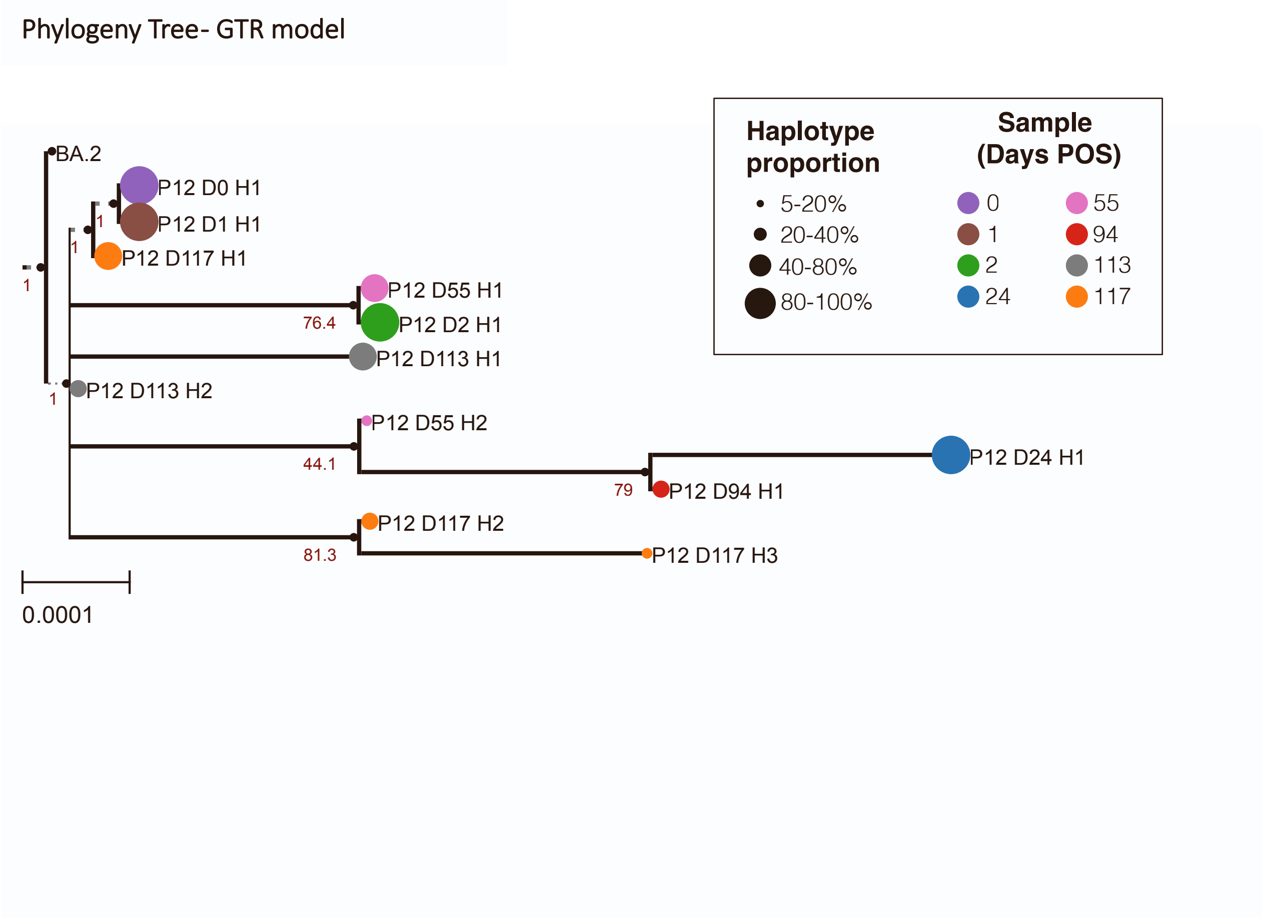

Figure S5N

Patient 14

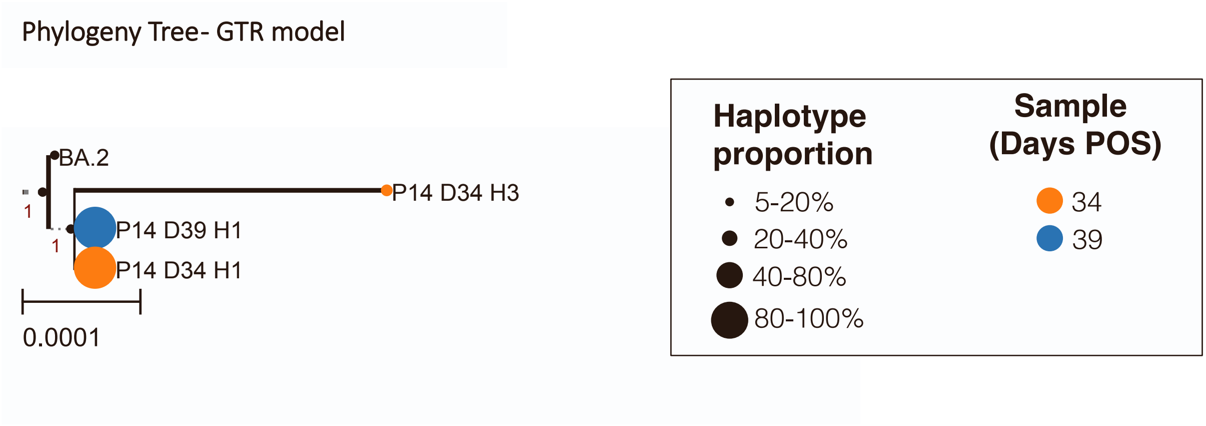

Figure S5O

Patient 15

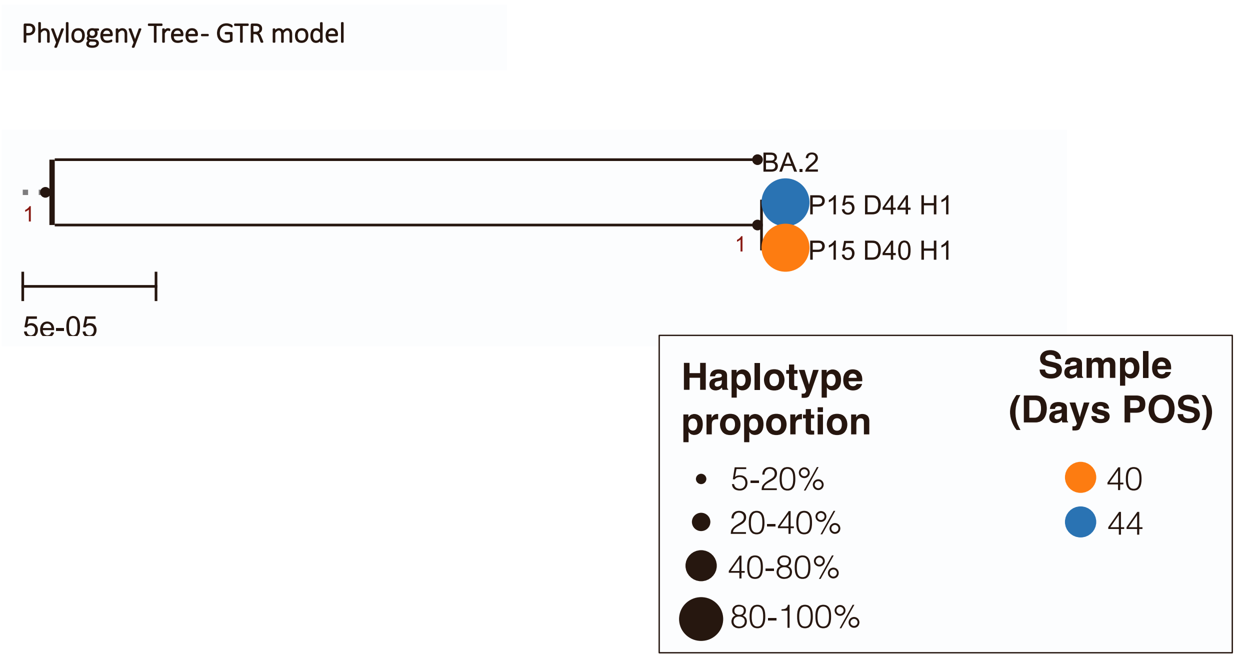

Figure S5P

Patient 16

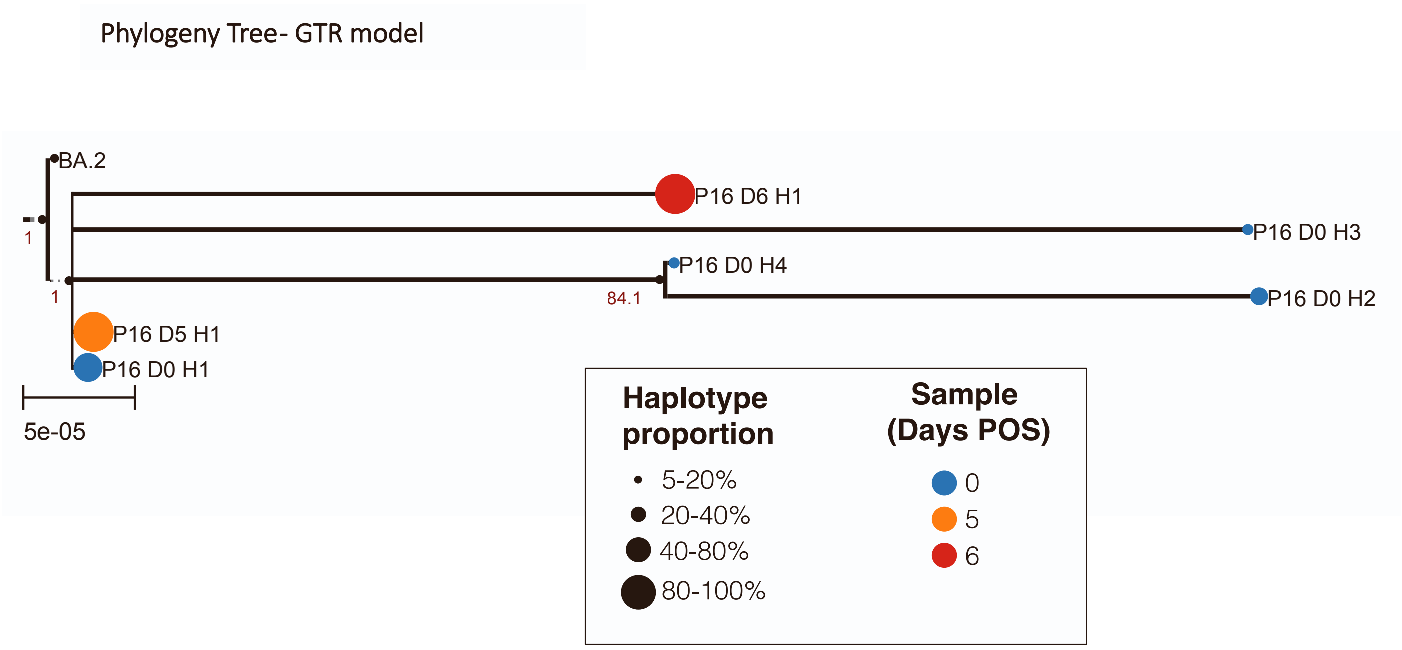

Figure S5Q

Patient 17

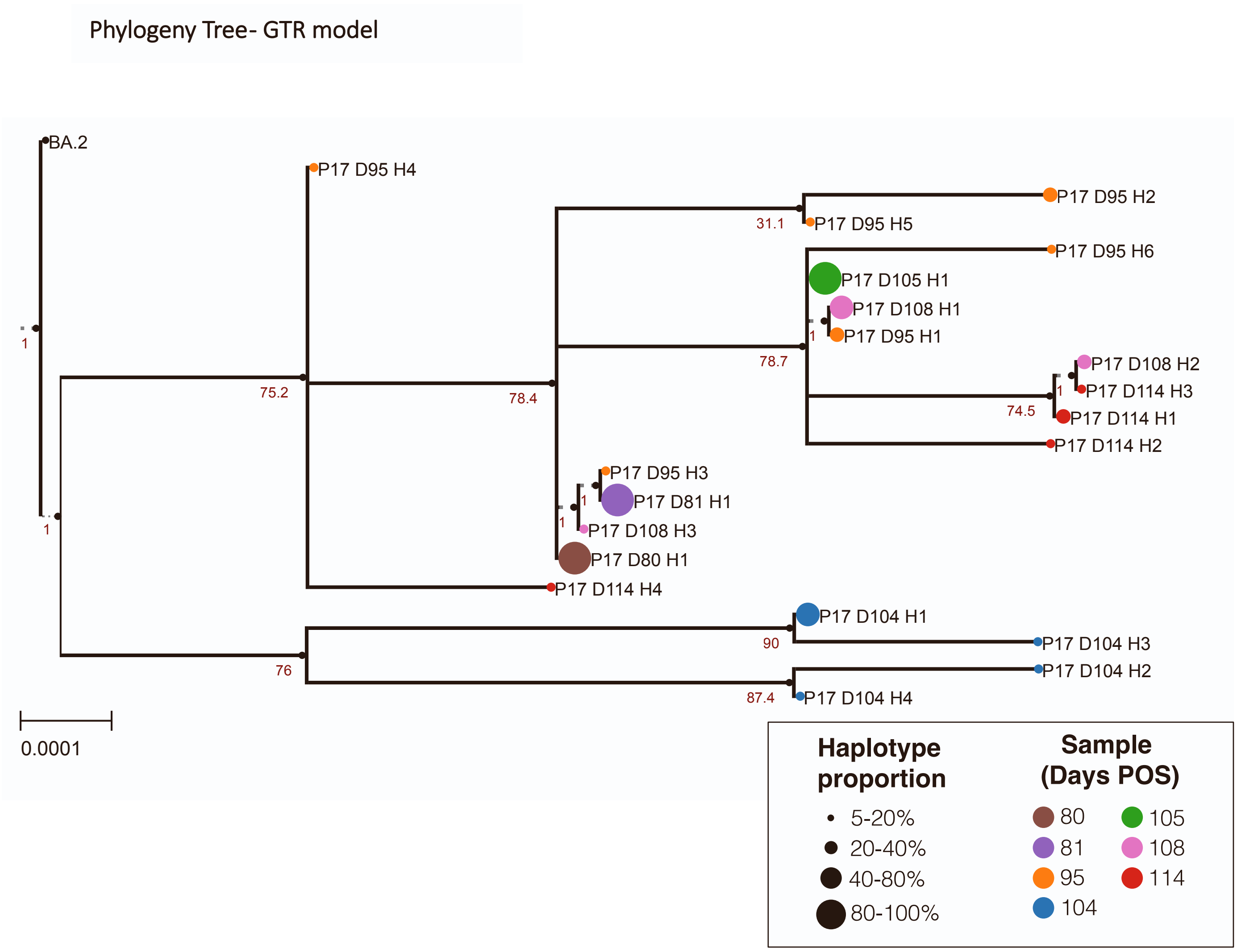

Figure S5R

Patient 18

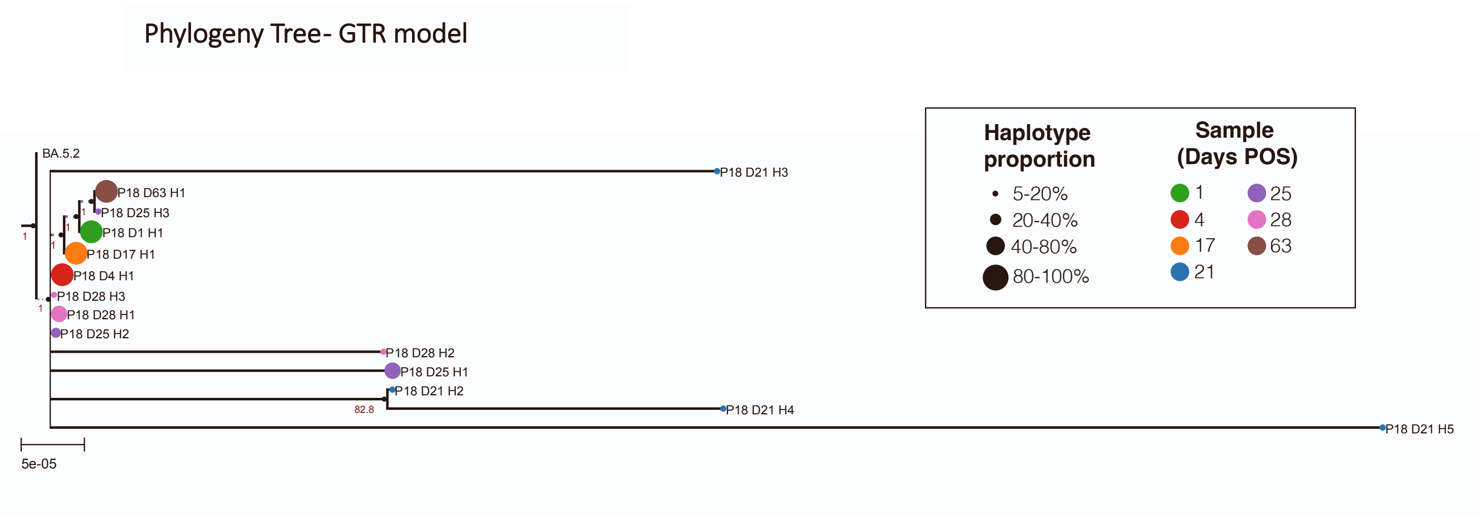

Figure S5S

Patient 19

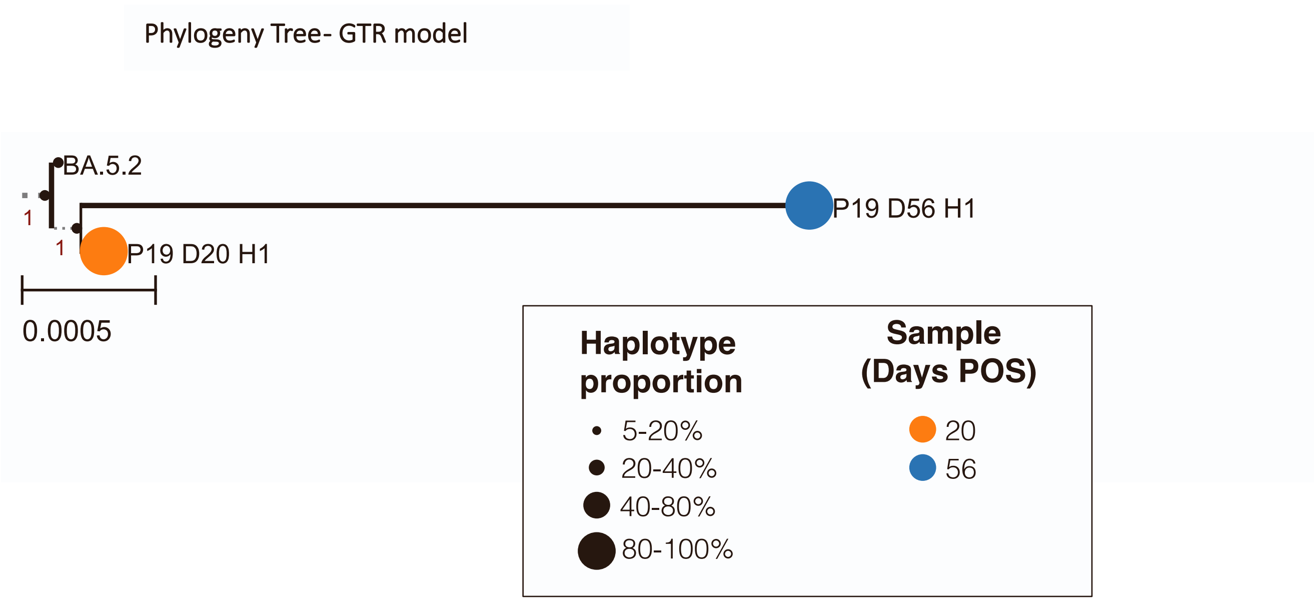

Figure S5T

Patient 20

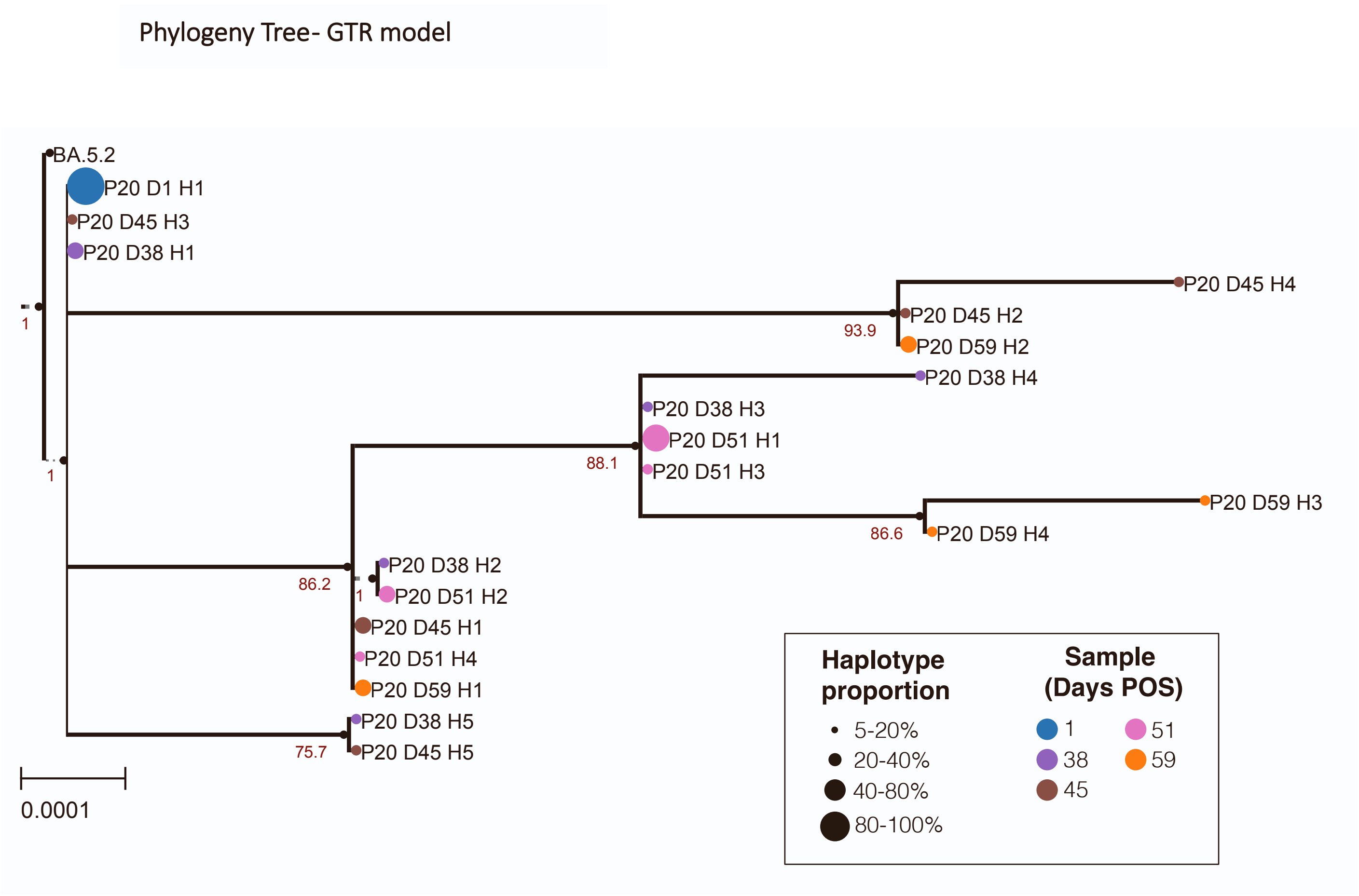

Figure S5U

Patient 21

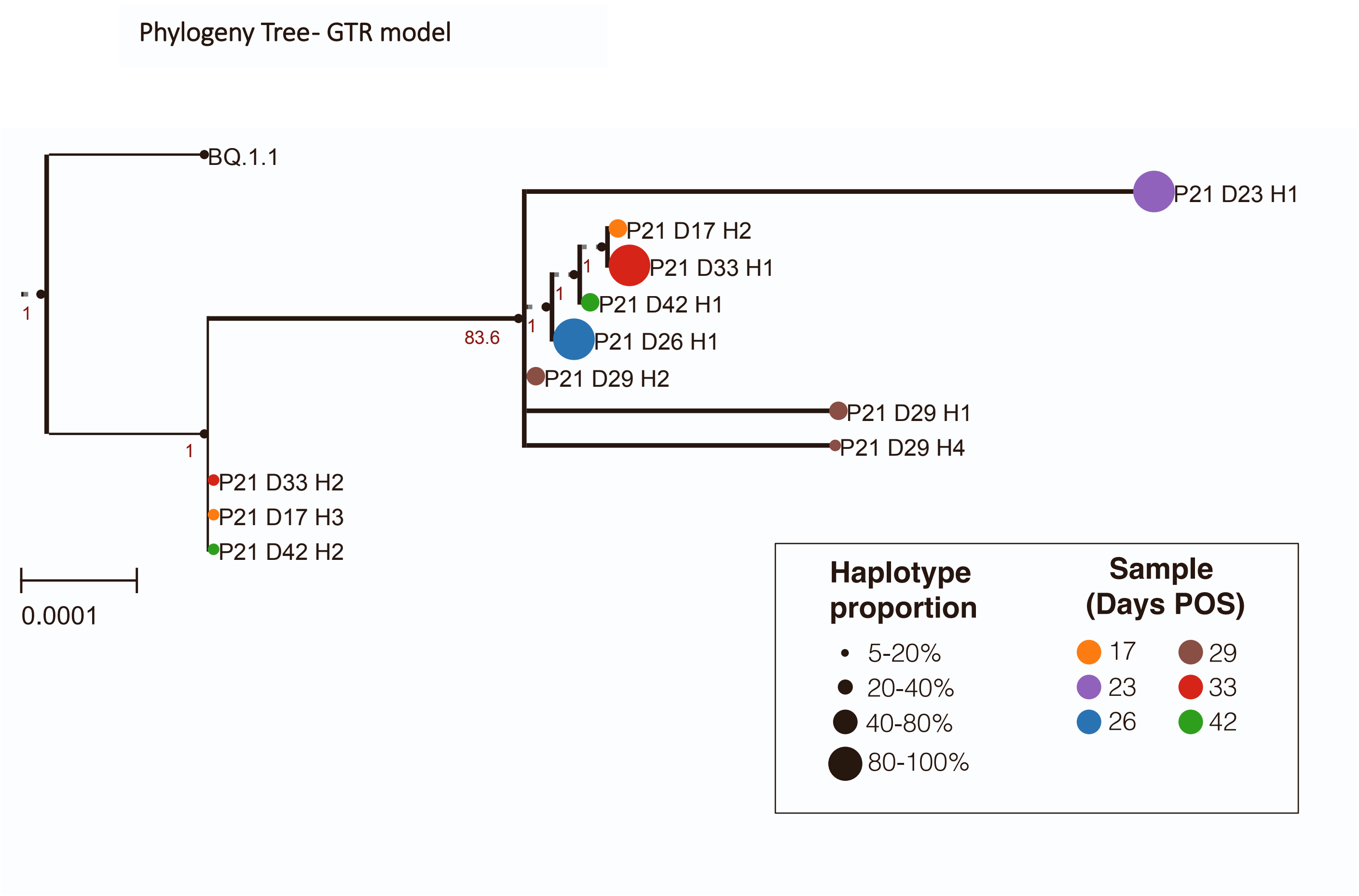

Figure S5V

Patient 22

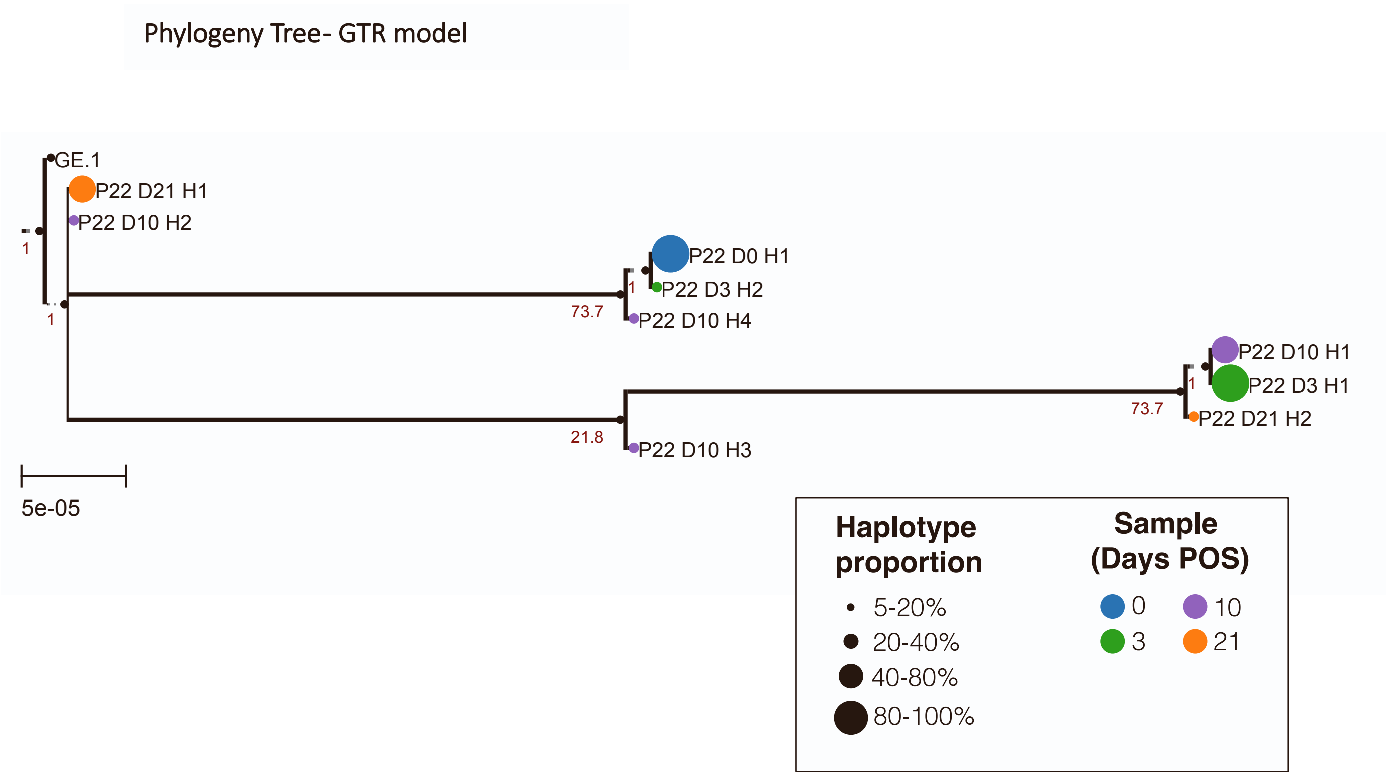

Figure S5W

Patient 23

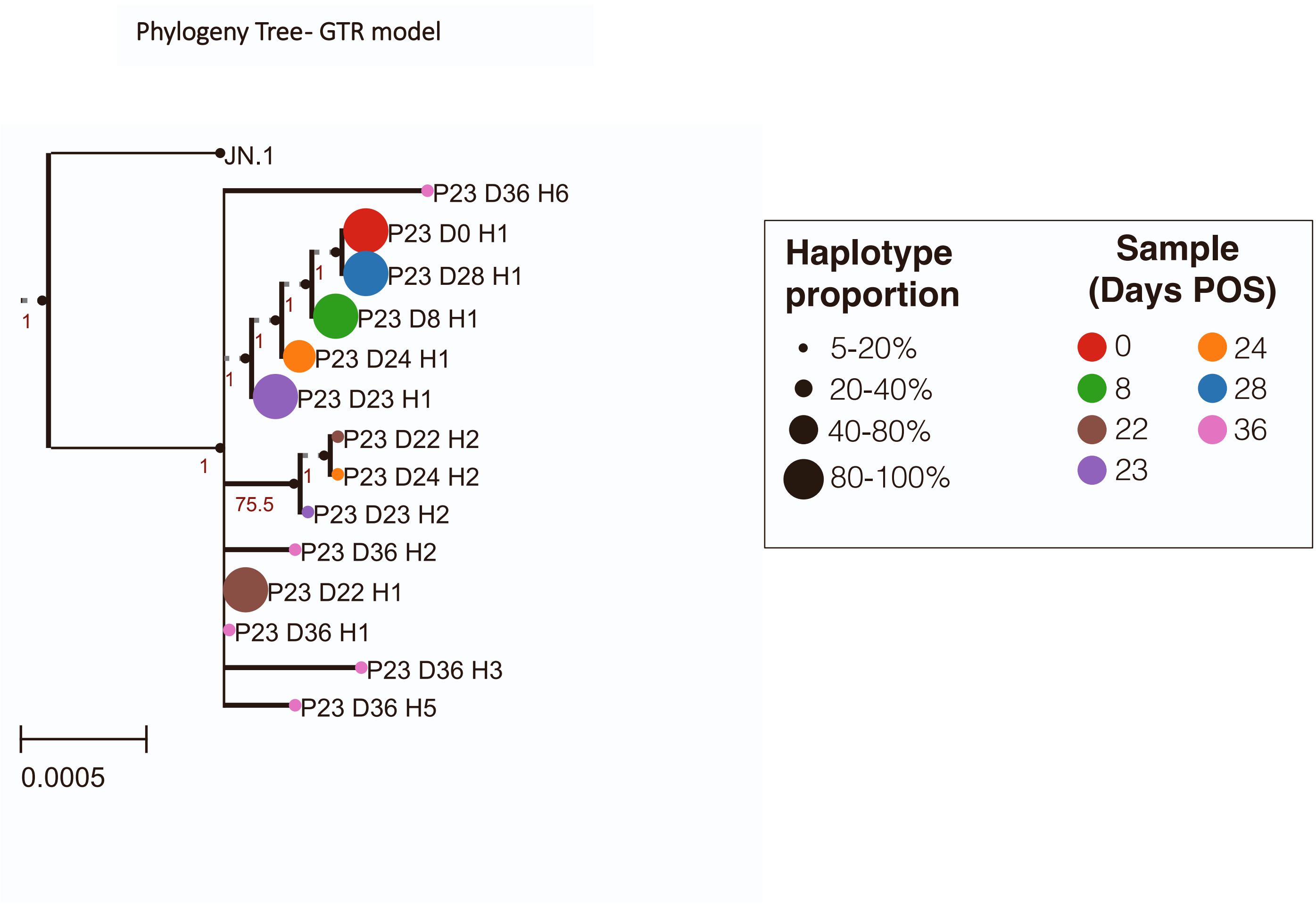

Figure S6
Detailed analyses of spike changes

A

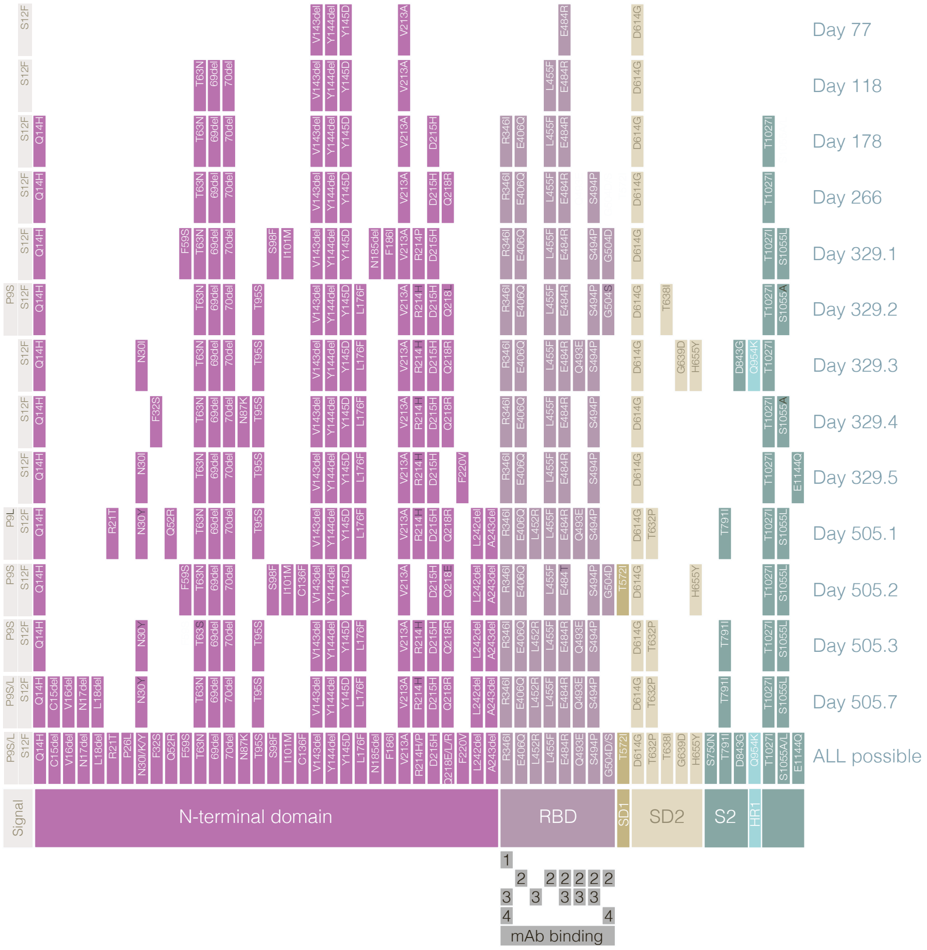

B

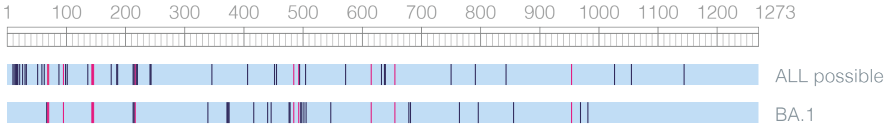

Figure S6. **Detailed analyses of RBD mutations over time and sequential escape from mAbs.** Related to Figure 5
(**A**) Detailed breakdown of the individual spike amino acid mutations shown in Figure 5A. Each amino acid mutation is listed. Spike haplotypes for days 329 and 505 are listed in order of frequency.
(**B**) Graphical comparison of the location of all observed mutations in P2 spike proteins with those found in the Omicron BA.1 spike. Red bars indicate identical positions of mutations.

Figure S7
Raw RLU data from Figure 5c and 5d

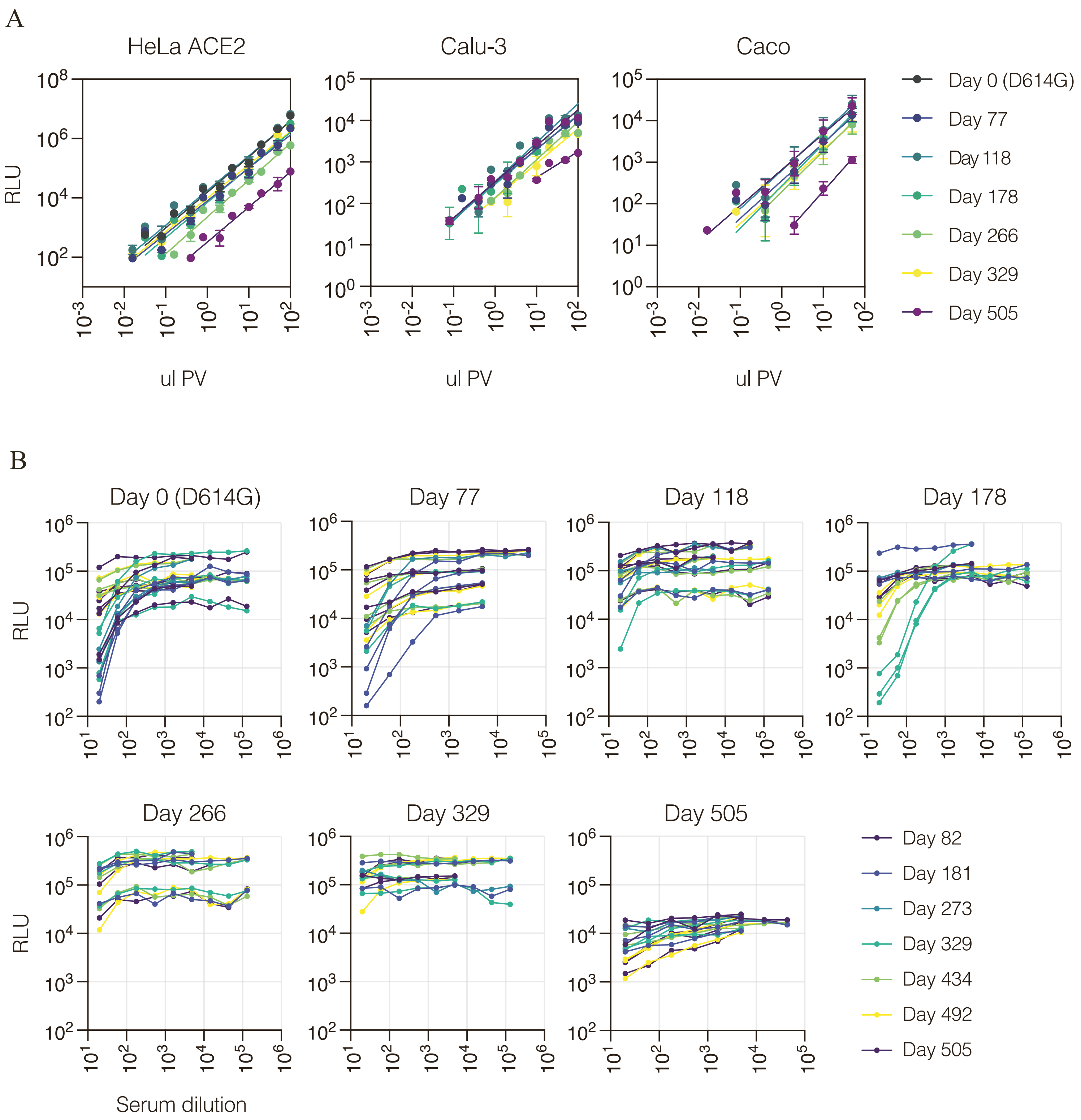

Figure S7. Raw RLU data related to Figures 5C and 5D
(**A**) Mean RLU/ul of each pseudovirus titrated on three different cell lines. Means and standard deviation are derived from three independent experiments.
(**B**) Raw RLU data from Figure 5D. Each replicate is graphed individually.

Figure S8

Neutralisation curves from Figure 5d and 5e

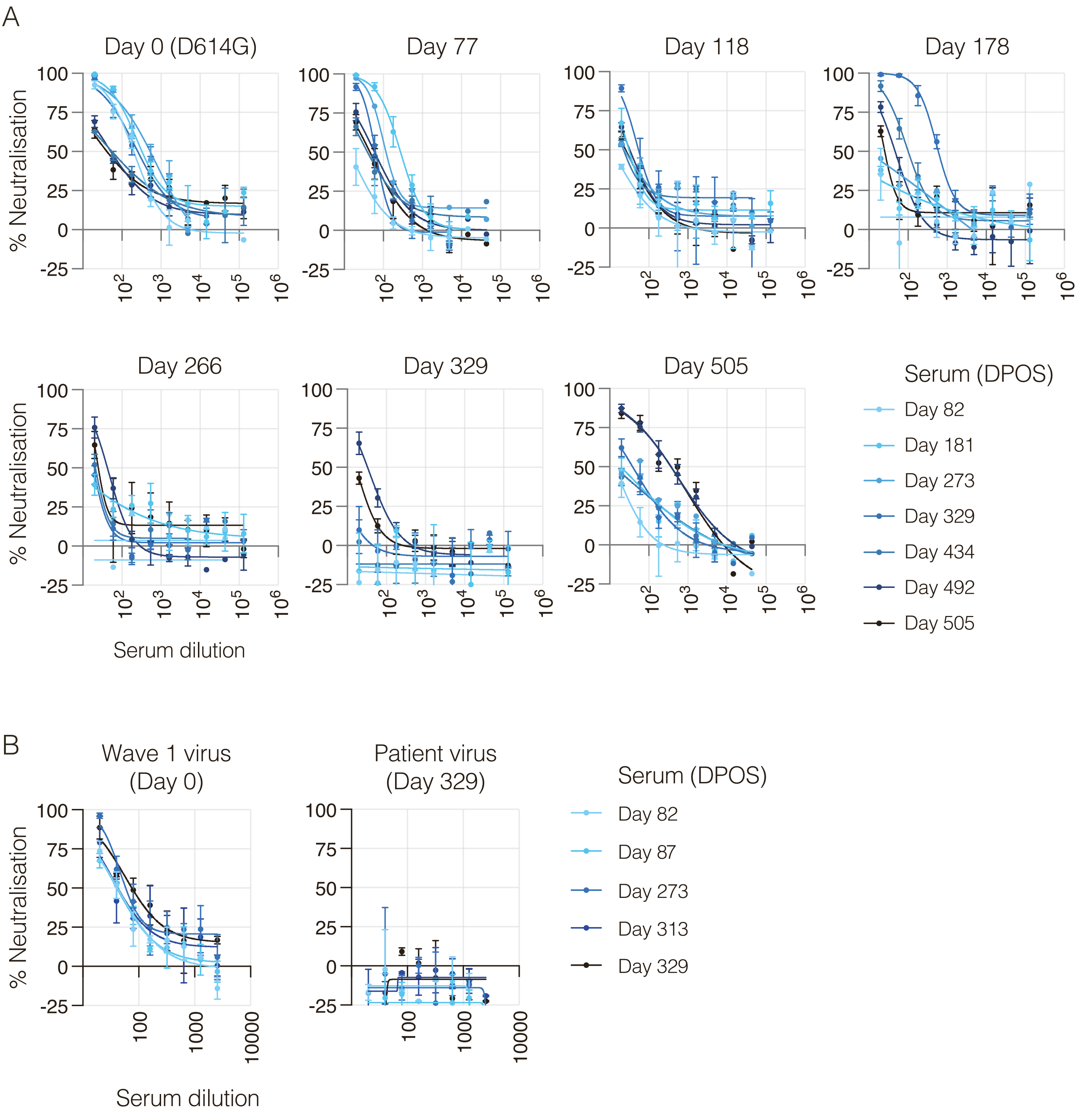

Figure S8. **Neutralisation curves related to Figures 5D and 5E**

(**A**) Individual neutralisation curves from Figure 5D.

(**B**) Individual neutralisation curves from Figure 5E.

Figure S9

Sera from other chronic infections

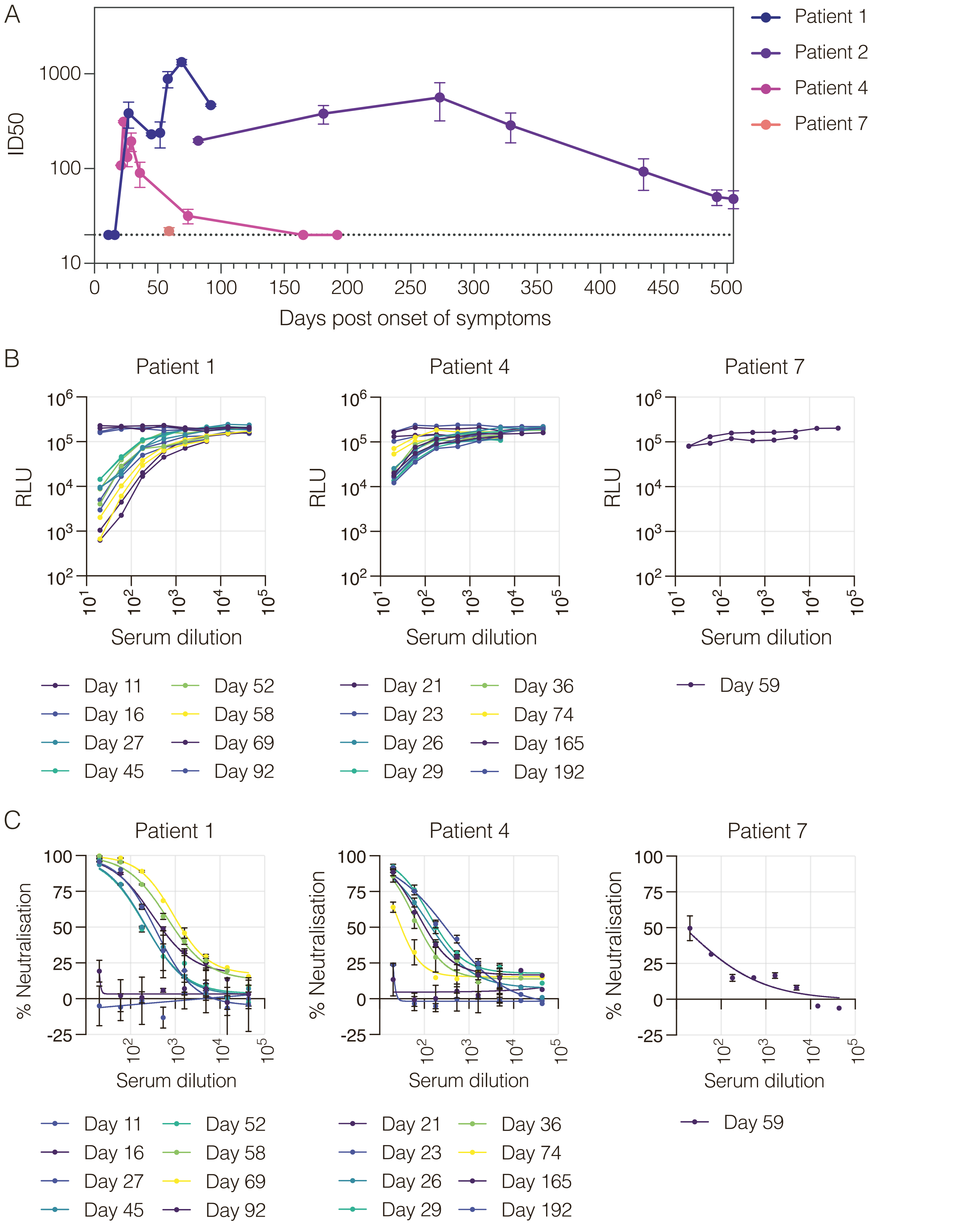

Figure S9. Sera from other chronic infections. Related to Figure 5D

(**A**) As for Figure 5D, longitudinal serum samples from patients 1, 2, 4 and 7 were tested in neutralisation assays against lentiviral vectors pseudotyped with D614G spike. Each point shows reciprocal mean neutralising titres (ID50) for a given serum sample, derived from two independent experiments. Error bars are standard deviation.

(**B**) Individual raw RLU values from (A).

(**C**) Individual neutralisation curves from (A).

Figure S10
Raw data and neutralisation curves from Figure 6a

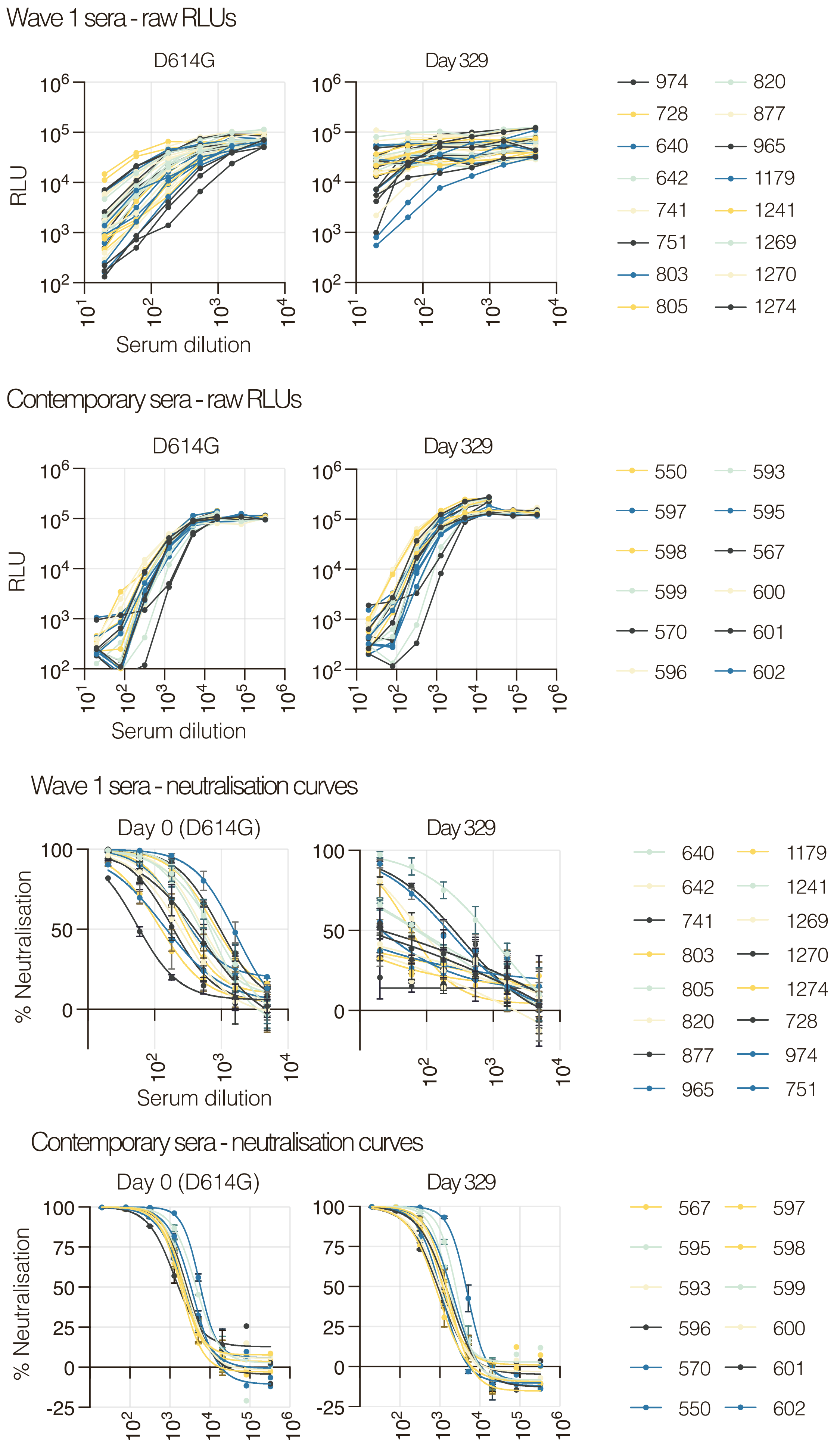

Figure S10. Raw RLU values and neutralisation curves related to Figure 6A. For the RLUs, each replicate is graphed individually; for the neutralisation curves, the means from two independent experiments are shown, with error bars representing standard deviation.

Figure S11

Detailed analyses of antibody escape

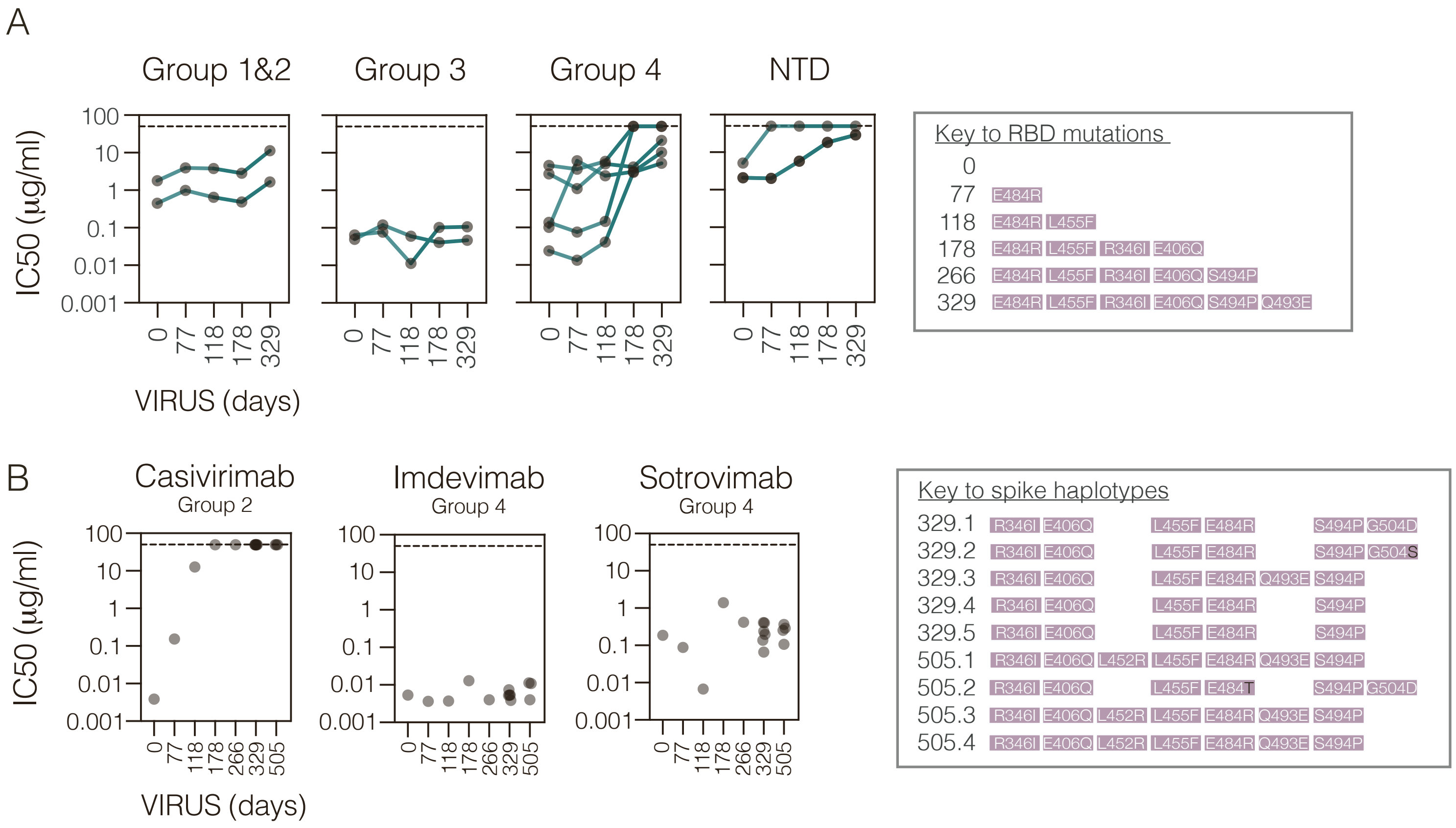

Figure S11. Related to Figure 6.

A) Neutralisation of spike from assumed infecting virus (day 0; ancestral D614G) and days 77, 118, 178 and 329, grouped according to mAb binding specificity. Each point is the mean IC50 value for a given mAb and spike combination, derived from two independent experiments. The key shows amino acid mutations in the RBD for each longitudinal spike.

(**B**) In depth neutralisation assays of longitudinal spike proteins from days 0, 77, 117, 178, 329 and 505, including multiple spike haplotypes for the day 329 and 505 time points, by commercial monoclonal antibodies. Each point is mean IC50 value derived from at least two independent experiments.

Table S1 - Clinical details of the cases of long-term persistent infection included in this study. Related to Figure 1.

Anon. ID	Age	Sex	Immunocompromise	CD20 depletion	Vaccin. (N)	Date Symptoms Onset	Date First Positive	Date Last Positive	Length (days)	Samples (N)	Lineage	Severity	Outcome	Treatment
P1	71	M	Kidney transplant	No	0	2020.04.06	2020.04.06	2020.07.03	89	4	B.1.2	Critical	Clear	Remdesivir (Date NA/TBC)
P2	23	M	Inherited immunodeficiency; unspecified	No	0	2020.04.17	2020.04.19	2021.09.04	506	9	B.1	Critical	Died	Remdesivir (Date NA/TBC)
P3	29	M	Bruton's hypogammaglobulinemia	No	0	2020.09.22	2020.10.05	2020.12.17	87	2	B.1.258	Severe	Clear	Ronapreve, Remdesivir (Date NA/TBC)
P4	57	M	Renal transplant, Diffuse large B-cell lymphoma	Yes	0	2020.03.28; 2020.12.16	2020.03.28; 2020.12.16	2020.05.05; 2021.02.05	39; 417	5	B.1.2; B.1.177.18	Moderate	Clear	Ronapreve, Remdesivir (Date NA/TBC)
P5	61	M	Advanced HIV	No	0	2021.01.03	2021.01.14	2021.03.15	72	8	B.1.1.7	Critical	Died	Remdesivir (Date NA/TBC)
P6	35	M	Advanced HIV	No	0	2021.01.09	2021.01.23	2021.04.22	104	4	B.1.1.7	Mild	Clear	Nil
P7	79	F	Rheumatoid arthritis,	Yes	0	2021.01.10	2021.01.20	2021.03.17	67	3	B.1.1.7	Critical	Died	Nil
P8	47	F	Advanced HIV	No	0	2021.12.01	2022.03.24	2022.06.17	199	11	AY.4	Mild	Clear	Remdesivir (Day 114), Regeneron (Date NA/TBC), Sotrovimab (Day 128)
P9	37	M	Hodgkin's lymphoma	Yes	3	2022.01.06	2022.01.22	2022.05.03	118	7	BA.1.1	Mild	Clear	Nil (TBC)
P10	67	F	Diffuse large B-cell lymphoma, CAR-T	Yes	3	2022.01.26	2022.01.27	2022.05.03	98	7	BA.2	Severe	Clear	Remdesivir (Day 37), Sotrovimab (Day 37), Paxlovid (Day 58)
P11	52	F	Thymoma	No	2	2022.02.09	2022.06.10	2022.06.17	129	1	BA.1.1	Moderate	Clear	Paxlovid (Day 128), Sotrovimab (Day 128)
P12	48	M	Non-Hodgkin's lymphoma, CAR-T	Yes	2	2022.03.20	2022.03.20	2022.10.07	202	8	BA.2	Mild	Clear	Sotrovimab (Day 2)
P13	52	M	Chronic lymphoid leukaemia	Yes	4	2022.03.21	2022.03.22	2022.11.14	239	1	BA.2	Moderate	Clear	Paxlovid (Day 5), Sotrovimab (Date NA/TBC)
P14	27	F	Lupus	Yes	3	2022.03.27	2022.04.30	2022.05.21	56	2	BA.2	Severe	Clear	Sotrovimab (Day 41)
P15	36	M	Burkitt's lymphoma	Yes	3	2022.04.08	2022.05.18	2022.06.02	56	2	BA.2	Mild	Clear	Nil (TBC)
P16	64	M	Diffuse large B-cell lymphoma	Yes	3	unknown	2022.04.19	2022.08.04	111	3	BA.2	Mild	Died	Remdesivir (Date TBC), Remdesivir (Day 17)
P17	62	M	Diffuse large B-cell lymphoma	Yes	4	2022.08.04	2022.07.10	2022.09.09	142	7	BA.2	Critical	Clear	Remdesivir (Days 39; 63), Sotrovimab (Days 54; 84), Paxlovid (Date TBC), Paxlovid + Remdesivir (Day 113)
P18	62	F	Cutaneous T-cell lymphoma	No	2	2022.07.12	2022.07.13	2022.09.08	59	7	BA.5.2	Mild	Died	Paxlovid pre-emptive (Date NA/TBC)
P19	57	F	Simultaneous pancreas & kidney transplant	Yes	3	2022.07.13	2022.08.02	2022.09.07	57	2	BA.5.2	Critical	Died	Sotrovimab (Day 20), Remdesivir (Date TBC)
P20	51	M	Kidney transplant	No	0	2022.09.27	2022.09.27	2022.12.01	66	5	BA.5.2	Mild	Unknown	Sotrovimab (Day 3), PO BD (???) (Day 3), Molnupiravir (Date NA/TBC)
P21	77	F	MALT lymphoma	Yes	4	2022.10.29	2022.11.15	2023.02.07	102	6	BQ.1.1	Severe	Clear	Paxlovid (Day 39), Sotrovimab (Day 40), Remdesivir (Day 40)
P22	64	M	Diffuse large B-cell lymphoma	Yes	3	2023.10.09	2023.10.09	2023.11.18	41	4	GE.1	Moderate	Clear	Remdesivir (Day 12), Paxlovid (Day 28)
P23	70	M	Myeloma	No	8	2023.11.23	2023.11.23	2024.01.12	51	7	JN.1	Critical	Died	Paxlovid (Day 27)

Table S3

SAMPLE	Q30+ full-spike reads	Haplotype 1		Haplotype 2		Haplotype 3		Haplotype 4		Haplotype 5		Haplotype 6		Haplotype 7	
		Reads	Percent (%)	Reads	Percent (%)	Reads	Percent (%)	Reads	Percent (%)	Reads	Percent (%)	Reads	Percent (%)	Reads	Percent (%)
1	11336	8236	72.65	2908	25.65	83	0.73	49	0.43						
	5000	3657	73.14	1273	25.46	36	0.72	18	0.36						
	2500	1853	74.12	642	25.68	0	0.00	0	0.00						
	1000	741	74.10	257	25.70	0	0.00	0	0.00						
	500	371	74.20	128	25.60	0	0.00	0	0.00						
	200	147	73.50	52	26.00	0	0.00	0	0.00						
2	5074	3987	78.58	1067	21.03										
	2500	1972	78.88	520	20.80										
	1000	767	76.70	231	23.10										
	500	384	76.80	113	22.60										
	200	158	79.00	50	25.00										
3	3892	3085	79.27	586	15.06	98	2.52	18	0.46	15	0.39	10	0.26	10	0.26
	2500	1965	78.60	385	15.40	70	2.80	11	0.44	0	0.00	0	0.00	0	0.00
	1000	773	77.30	166	16.60	27	2.70	0	0.00	0	0.00	0	0.00	0	0.00
	500	405	81.00	90	18.00	0	0.00	0	0.00	0	0.00	0	0.00	0	0.00
	200	156	78.00	41	20.50	0	0.00	0	0.00	0	0.00	0	0.00	0	0.00
4	2074	593	28.59	538	25.94	301	0.145	298	0.144	78	3.76	73	3.52	11	0.53
	1000	320	32.00	268	26.80	162	0.162	152	0.152	39	3.90	26	2.60	0	0.00
	500	151	30.20	131	26.20	83	0.166	81	0.162	21	4.20	20	4.00	0	0.00
	200	70	35.00	52	26.00	34	0.17	29	0.145	0	0.00	0	0.00	0	0.00
5	3465	1763	50.88	661	19.08	591	17.06	191	5.51	94	2.71	45	1.30		
	2500	1305	52.20	482	19.28	437	17.48	143	5.72	70	2.80	32	1.28		
	100	521	521.00	190	190.00	181	181.00	60	60.00	23	23.00	14	14.00		
	500	264	52.80	97	19.40	79	15.80	31	6.20	17	3.40	0	0.00		
	200	101	50.50	46	23.00	45	22.50	0	0.00	0	0.00	0	0.00		

Table S3. **Impact of down-sampling on the reproducibility of spike haplotype determination in clinical samples**. HaploVar v1.0 workflow was used on five clinical samples to obtain high-quality Q30-positive full-spike reads, which were then serially diluted down to 200 Q30-positive reads. The reproducibility of determining spike haplotype frequencies was subsequently assessed for all samples and dilutions. Related to Figure 1 and STAR Methods.

Table S4

SAMPLE	REPLICATE 1				REPLICATE 2			
	Q30 Reads	Haplotype Rank	Reads	Haplotype Proportion	Q30 Reads	Haplotype Rank	Reads	Haplotype Proportion
1	844	1	459	54.38	2030	1	1233	60.74
1	844	2	52	6.16	2030	3	112	5.52
1	844	3	52	6.16	2030	2	123	6.06
1	844	4	34	4.03	2030	6	46	2.27
1	844	5	33	3.91	2030	4	79	3.89
1	844	6	18	2.13	2030	5	48	2.36
1	844	7	13	1.54	2030	7	25	1.23
1	844	8	11	1.30	2030	8	0	0.00
2	1972	1	512	25.96	1827	1	591	32.35
2	1972	2	406	20.59	1827	2	536	29.34
2	1972	3	325	16.48	1827	4	294	16.09
2	1972	4	271	13.74	1827	3	309	16.91
2	1972	5	124	6.29	1827	5	78	4.27
2	1972	6	104	5.27	1827	6	71	3.89
2	1972	7	10	0.51	1827	8	10	0.55
2	1972	no	0	0.00	1827	7	12	0.66
3	388	1	200	51.55	480	1	369	76.88
3	388	2	117	30.15	480	2	72	15.00
3	388	3	60	15.46	480	3	17	3.54
4	3892	1	3085	79.27	8215	1	6419	78.14
4	3892	2	586	15.06	8215	2	1383	16.84
4	3892	3	98	2.52	8215	3	269	3.27
4	3892	4	18	0.46	8215	5	15	0.18
5	11336	1	3158	27.86	5185	1	8236	158.84
5	11336	2	1804	15.91	5185	2	2908	56.08
5	11336	3	183	1.61	5185	3	0	0.00
5	11336	4	21	0.19	5185	4	0	0.00
6	3393	1	1763	51.96	1710	3	340	19.88
6	3393	2	661	19.48	1710	6	32	1.87
6	3393	3	591	17.42	1710	1	591	34.56
6	3393	4	191	5.63	1710	2	510	29.82
6	3393	5	94	2.77	1710	5	133	7.78
6	3393	6	0	0.00	1710	4	16	0.94
7	747	1	335	44.85	2189	1	1160	52.99
7	747	2	59	7.90	2189	2	202	9.23
7	747	3	44	5.89	2189	3	186	8.50
7	747	4	43	5.76	2189	4	173	7.90
7	747	5	33	4.42	2189	5	98	4.48
7	747	6	32	4.28	2189	9	23	1.05
8	5074	1	3828	75.44	4605	1	3987	86.58
8	5074	2	766	15.10	4605	2	1067	23.17
9	909	1	486	53.47	1259	1	1658	131.69
9	909	2	375	41.25	1259	2	494	39.24
9	909	3	37	4.07	1259	3	62	4.92

Table S4. Replicate identification and quantification of full-spike haplotypes in clinical samples (n=9). Related to Figure 1, Figure S2 and STAR Methods.

Table S5 - Molecular clock (s/s/y) evolutionary rates estimated for the infecting variant spike of each patient or selected VOC spikes. Where the R^2 is greater than 0.75 the difference in rate compared to contemporaneous rate is given. Related to Figure 3.

		Molecular Clock (s/s/y)	R^2	(Residual mean)^2	Fold Change related to contemporaneous VOC
Global whole genome estimates		5.8×10^{-4} to 9.9×10^{-4} (†)			
Lineage & Spike specific					
B.1 spike		5.08×10^{-4}	0.19	5.60×10^{-8}	
B.1.1.7 spike		5.71×10^{-4}	0.13	1.29×10^{-7}	
Anonymised ID / Lineage					
P1	B.1.2	2.33×10^{-3}	0.47	3.34×10^{-8}	
P2	B.1	4.13×10^{-3}	0.87	4.48×10^{-7}	8.13 x
P3	B.1.258	1.50×10^{-3}	0.54	8.93×10^{-8}	
P4	B.1.2	6.40×10^{-3}	0.29	1.13×10^{-7}	
	B.1.177.18	\\$3.00 \times 10^{-3}$	0.81	1.41×10^{-8}	2.95x
P5	B.1.1.7	1.16×10^{-3}	0.20	6.92×10^{-8}	
P6	B.1.1.7	1.70×10^{-3}	0.20	2.60×10^{-7}	
P7	B.1.1.7	1.16×10^{-3}	0.42	6.88×10^{-8}	
P8	AY.4	1.32×10^{-3}	0.25	4.05×10^{-7}	
P9	BA.1.1	2.70×10^{-3}	0.26	1.22×10^{-7}	
P10	BA.2	1.15×10^{-3}	0.28	4.22×10^{-8}	
P11	BA.1.1	3.54×10^{-3}	0.92	8.87×10^{-8}	2,31x
P12	BA.2	4.49×10^{-4}	0.13	5.70×10^{-8}	
P13	BA.2	ND	NA	NA	
P14	BA.2	1.88×10^{-4}	0.11	2.31×10^{-8}	
P15	BA.2	1.00×10^{-3}	1.0	5.43×10^{-11}	0.65x
P16	BA.2	5.38×10^{-4}	0.17	4.36×10^{-8}	
P17	BA.2	1.20×10^{-3}	0.40	5.57×10^{-8}	
P18	BA.5.2	1.99×10^{-4}	0.19	4.28×10^{-8}	
P19	BA.5.2	2.05×10^{-3}	0.35	3.02×10^{-6}	
P20	BA.5.2	7.47×10^{-3}	0.34	2.24×10^{-7}	
P21	BQ.1.1	1.50×10^{-3}	0.40	1.88×10^{-8}	
P22	GE.1	1.26×10^{-3}	0.19	3.01×10^{-7}	
P23	JN.1	5.91×10^{-3}	0.76	5.30×10^{-8}	3.86x

(†) Chaguza et al. 2023; Duchene et al. 2020
